# Microwave-assisted synthesis of 2-substituted 4,5,6,7-tetrahydro-1,3-thiazepines from 4-aminobutanol

**DOI:** 10.3762/bjoc.16.5

**Published:** 2020-01-06

**Authors:** María C Mollo, Natalia B Kilimciler, Juan A Bisceglia, Liliana R Orelli

**Affiliations:** 1 Universidad de Buenos Aires. CONICET. Departamento de Química Orgánica, Facultad de Farmacia y Bioquímica. Junín 956, (1113) Buenos Aires, Argentina

**Keywords:** cyclodehydration, medium-size heterocycles, microwaves, PPSE, tetrahydrothiazepines

## Abstract

A general procedure for the synthesis of 2-substituted tetrahydro-1,3-thiazepines by MW-assisted cyclization of 4-thioamidobutanols is presented. The acyclic precursors were prepared in high overall yields by an expeditious three-step diacylation/thionation/deprotection sequence from 4-aminobutanol. Microwave-assisted ring closure of 4-thioamido alcohols promoted by trimethylsilyl polyphosphate (PPSE) in solvent-free conditions allowed for the synthesis of several hitherto unreported seven-membered iminothioethers bearing 2-aryl, alkenyl, aralkyl and alkyl substituents. The cyclodehydration reaction is likely to involve an S_N_2-type displacement and affords good to excellent yields of the desired heterocycles in very short reaction times.

## Introduction

Thiazolines (4,5-dihydrothiazoles) are widely studied heterocycles due to their multiple applications as antibiotics [1], antiproliferative agents [2], anti-inflammatories [3] and antithrombotics [4]. They are also found in many bioactive natural products [5–8], some of which display anti-HIV-1 [9] and antimitotic activities [10], among others. Thiazolines also find use in organic synthesis as building blocks in drug discovery, and as ligands in metal-catalyzed reactions [11–16]. Their six-membered homologues (5,6-dihydro-4*H*-1,3-thiazines) are also compounds of broad interest [17], due to their bioactivities [18–19] and as valuable synthetic intermediates [20–22].

On the other hand, the seven-membered iminothioethers (4,5,6,7-tetrahydro-1,3-thiazepines) have remained almost unexplored. This virtual void is probably due to the known difficulty of the annulation of seven-membered heterocyclic rings. In fact, ring closure reactions leading to medium-sized heterocycles are hampered by unfavourable entropic and enthalpic factors, and are usually characterized by harsh reaction conditions, long reaction times, low yields and/or competitive intermolecular reactions [23–28].

In 1963, 2-(2,6-dichlorophenyl)-4,5,6,7-tetrahydro-1,3-thiazepine was prepared by a double displacement reaction between 2,6-dichlorothiobenzamide and 1,4-butylene dibromide [29]. In 1970, Mente prepared a dehydro analogue, namely 2-(4-chlorophenyl)-4,7-dihydro-1,3-thiazepine, via thermolysis of *N-*(4-chlorothiobenzoyl)-2-vinylaziridine [30]. No yields were reported for these reactions in either case. The synthesis of 2-phenyl-4,5,6,7-tetrahydro-1,3-thiazepine by cyclodehydration of *N-*(4-hydroxybutyl)thiobenzamide was attempted with different condensing agents: PPA (100 °C, 15 h), Burgess (25 °C, 48 h) and PEG-Burgess (80 °C, 2 h) reagents, and under Mitsunobu conditions. In the first case, the ^1^H NMR spectrum of the resulting product contained no signals in the aromatic region [31]. The Burgess reagent led to 58% of the desired product, while the modified PEG-Burgess reagent yielded a mixture of the thiazepine (17%) and the isomeric *N-*thiobenzoylpyrrolidine (40%). The use of Mitsunobu conditions led exclusively to the pyrrolidine derivative (76%) [32]. Therefore, the few literature examples reported until now are either complicated by side reactions or involve long reaction times and/or modest yields.

Polyphosphoric acid (PPA) esters PPE (ethyl polyphosphate) and PPSE (trimethylsilyl polyphosphate) are powerful and versatile dehydrating agents that find many applications in organic synthesis [33–42]. Their preparation is operationally simple and requires readily available and inexpensive starting materials. In addition, they are stable, non-toxic and environmentally safe. These reagents play a dual role, as they enhance the electrophilicity of nitriles, carbonyls and carbinols, and can also react irreversibly with water. Unlike PPA, a Brønsted acid incompatible with some acid-sensitive functionalities, PPE and PPSE are aprotic and mild reagents, with wide functional group tolerance. The use of PPA has some operational disadvantages: it is an extremely viscous liquid that requires high temperatures to allow stirring of the reaction mixture, in which organic compounds do not generally dissolve, while PPA esters are soluble in many organic solvents [43].

Ring closure reactions leading to different heterocyclic cores are relevant among the synthetic applications of PPA esters. The use of MW irradiation as the energy source in these reactions [44–47] is of particular interest due to the many advantages associated with MAOS: very short reaction times, minimization of side products and consequent increment of the yields. To our knowledge, however, PPA esters had not been tested yet for the synthesis of cyclic iminothioethers.

As part of an ongoing program on the applicability of PPE and PPSE to the synthesis of nitrogen-containing heterocycles [48–54], we developed a general method for the preparation of five- and six-membered cyclic iminoethers (2-oxazolines and 5,6-dihydro-4*H*-1,3-oxazines, respectively), by MW-assisted ring closure of amido alcohols promoted by PPA esters [54]. PPE and PPSE were then tested for the synthesis of the more challenging seven-membered heterocycles, namely 4,5,6,7-tetrahydro-1,3-oxazepines. Instead of the target compounds, the reactions led to the corresponding *N-*acylpyrrolidines, as disclosed by Hoogenboom et al. [55]. These results were intriguing, since the synthesis of some seven- and even eight-membered 1,3-diheterocycles had already been achieved by our group using the PPA ester/MW system [48,50,52–53].

Considering the lack of efficient methods for 4,5,6,7-tetrahydro-1,3-thiazepines, we decided to explore their synthesis by microwave-assisted ring closure of *N-*(4-hydroxybutyl)thioamides promoted by PPA esters. The preparation of these acyclic precursors is not straightforward, as it involves a selective thionation step.

We present herein a novel method for the synthesis of seven-membered cyclic iminothioethers from commercially available 4-aminobutanol. The sequence allows the preparation of 4,5,6,7-tetrahydro-1,3-thiazepines bearing 2-aryl, aralkyl, alkenyl and alkyl substituents.

## Results and Discussion

### Synthesis of the acyclic precursors

As previously mentioned, some difficulties were anticipated regarding the synthesis of 4-thioamidobutanols. In fact, the direct thionation of the corresponding 4-amido alcohols cannot be used, as the OH is almost as reactive as the amide carbonyl toward Lawesson's reagent, the most commonly used thionating agent [56]. Two syntheses of ω-thioamidobutanols were recently reported by Nguyen et al. The first one afforded *N-*thiobenzoylaminobutanol in 62% yield [57], and the second one is not applicable to *N-*thioaroyl derivatives [58]. Thus, a diacylation–thionation–saponification sequence [59] was implemented (Table 1), and the individual steps optimized using 4-aminobutanol and benzoyl chloride as the starting materials.

**Table 1:** Synthesis of compounds **3**.

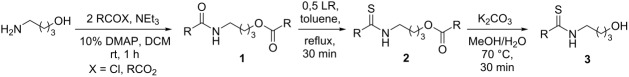					

Compd.	R	**1**	**2**	**3**	Overall yield [%]^a^

**a**	C_6_H_5_	95^b^	95	95	85
**b**	4-ClC_6_H_4_	90^b^	93	87	73
**c**	4-CH_3_C_6_H_4_	93^b^	95	82	72
**d**	4-OCH_3_C_6_H_4_	95^b^	93	87	77
**e**	4-NO_2_C_6_H_4_	90^b^	quant.	91	82
**f**	2,4-Cl_2_C_6_H_3_	94^b^	87	98	80
**g**	2-FC_6_H_4_	96^b^	90	90	78
**h**	2-CH_3_C_6_H_4_	90^b^	92	83	69
**i**	C_6_H_5_CHCH	95^b^	95	80	72
**j**	C_6_H_5_CH_2_	86^b^	80	80	55
**k**	C_5_H_10_	70^b^	quant.	93	65
**l**	isopropyl	93^c,d^	85	71^e^	56
**m**	*tert-*butyl	77^c,d^	87	89^e^	59

^a^Yields correspond to pure compounds. ^b^The reaction was performed with the acid chloride. ^c^The reaction was performed with the anhydride. ^d^The reaction was carried out at reflux for 48 h. ^e^The reaction was carried out using 10% NaOH/methanol at reflux for 4 h.

The diacylation step using pyridine as the base required 24 h for completion and gave a disappointingly low yield due to partial hydrolysis of the ester moiety, while triethylamine performed comparatively better (80%, 24 h). Finally, addition of 10 mol % of DMAP led to a higher yield (95%) in 1 h at room temperature. Using the optimized protocol, compounds **1a–k** were synthesized in high to excellent yields (Table 1). The more sterically hindered derivatives **1l**,**m** required significantly longer reaction times.

Thionation of the resulting amidoester **1a** with Lawesson's reagent (LR) was then examined. Using the reagent in excess, (LR/substrate 2.5:1, toluene, reflux) the reaction was chemoselective toward the amide group, and compound **2a** was obtained in high yield (93%), although the unreacted LR complicated the isolation and purification of the product. Lowering the molar ratio of the starting materials to strictly equivalent 0.5:1 maintained the yield (95%) and considerably simplified the procedure, suppressing collateral products arising from decomposition of the reagent. Under these conditions, compounds **2a–m** were synthesized in excellent yields (Table 1).

Cleavage of the thioamide-ester **2a** with K_2_CO_3_ in methanol/water (30 min, 70 °C) afforded thioamido alcohol **3a** (95%). Thioaroyl derivatives **3b–k** were prepared in high to quantitative yields under these experimental conditions, except for the more sterically hindered 2-methylphenyl derivative **2h**, which required a longer reaction time (1 h) to achieve complete conversion to the desired product. Saponification of the less reactive isobutyryl and pivaloyl derivatives **2l**,**m** required the use of a stronger base, longer reaction times and higher temperature (10% NaOH, reflux, 4 h).

The overall output of the optimized sequence (Table 1, last column) ranged from 69 to 85% for thioaroyl derivatives, and compares favourably with the literature data [57] for **3a** (85 vs 62%, respectively). Compounds **3j**,**l**,**m** afforded slightly lower overall yields.

### Synthesis of tetrahydro-1,3-thiazepines

With the required precursors in hand, we examined next the MW-assisted heterocyclization of *N-*thiobenzoylaminobutanol promoted by PPA esters. Using PPE/CHCl_3_, irradiation of precursor **3a** in a closed vessel MW reactor (1 min, 90 °C) afforded tetrahydrothiazepine **4a** in 61% yield. The same reaction was then tested with PPSE/CH_2_Cl_2_ as the dehydrating agent, leading to poorer results (30% yield). Based on our previous experience on related heterocycles, the cyclization was performed with PPSE under solvent-free conditions (8 min, 90 °C), delivering 73% of compound **4a** as the only product.

**Scheme 1:**
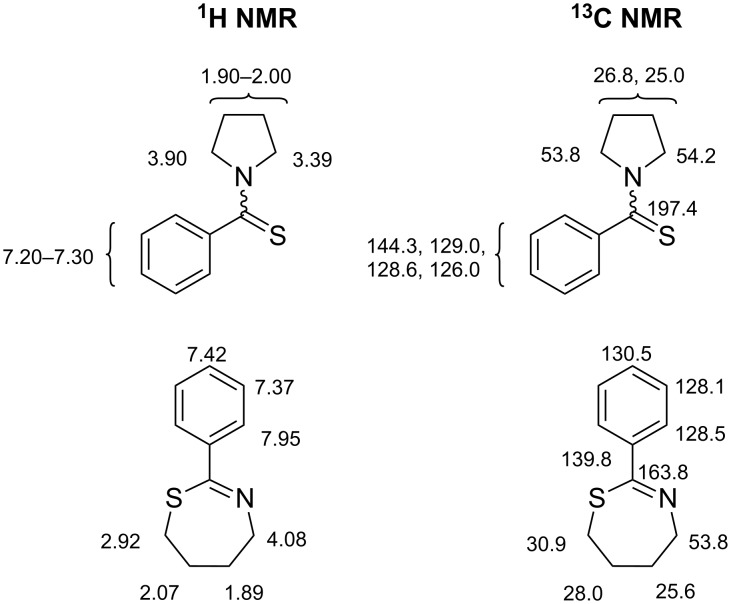
Chemical shift data for *N-*thiobenzoylpiperidine and compound **4a**.

As already mentioned, Wipf et al*.* had previously reported that cyclization of *N-*thiobenzoylaminobutanol with PEG-linked Burgess reagent and under Mitsunobu conditions led to significant amounts of *N-*thiobenzoylpyrrolidine [32]. In fact, this kinetically favoured competitive reaction also occurs in the closely related cyclization of *N-*benzoylaminobutanol with PPSE, which leads exclusively to *N-*acylpyrrolidines. In order to ascertain the structure of compound **4a**, we compared its NMR spectra with those of *N-*thiobenzoylpyrrolidine (Scheme 1) [60], confirming further the identity of the tetrahydrothiazepine by analysis of its 2D NMR spectra (see Supporting Information File 1).

Using the optimized experimental conditions, several novel 2-aryl-1,4,5,6-tetrahydro-1,3-thiazepines were synthesized in good to high yields (Table 2). Due to the different reactivity of each substrate, irradiation times and temperatures were individually adjusted by monitoring the disappearance of substrate (TLC). Derivatives **4f–h**, bearing *ortho*-substituted phenyl groups afforded slightly lower yields. Our results compare favourably with the previously reported data (58% yield of **4a**, reaction time 48 h) as the method combines the efficiency of PPSE with the advantages of MAOS: very short reaction times and suppression of collateral products. In view of the encouraging results obtained hitherto, we examined next the applicability of the method to the synthesis of derivatives bearing non-aromatic 2-substituents, of which no examples were found in the literature. Previous studies on related cyclic iminoethers [54] had shown that 2-alkyl derivatives are comparatively more prone to hydrolysis, require harsher reaction conditions and afford significantly lower yields. Despite this, and in order to extend the scope of the method, we attempted the cyclization of some representative examples of this type of substitution. The necessary precursors were prepared by the sequence depicted in Table 1. Regarding the cyclodehydration reaction, in the same experimental conditions than before (neat PPSE, 1–8 min, 90 °C), thioamidoalcohols **3i–k** afforded the corresponding tetrahydro-1,3-thiazepines **4i–k** in good to high yields as the only products (Table 2). Ring closure of the more sterically hindered isobutyryl and pivaloyl thioamidoalcohols **3l**,**m** was also successful, requiring slightly higher temperatures.

**Table 2:** Synthesis of 4,5,6,7-tetrahydrothiazepines **4**.

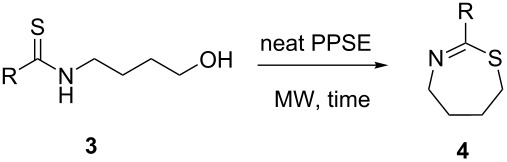				

Compd. **4**	R	Time (min)	Temp. (°C)	Yield (%)^a^

**a**	C_6_H_5_	8	90	73
**b**	4-ClC_6_H_4_	1	90	82
**c**	4-CH_3_C_6_H_4_	1	90	72
**d**	4-OCH_3_C_6_H_4_	1	90	80
**e**	4-NO_2_C_6_H_4_	2	90	65
**f**	2,4-Cl_2_C_6_H_3_	2	90	70
**g**	2-FC_6_H_4_	3	90	64
**h**	2-CH_3_C_6_H_4_	4	90	65
**i**	C_6_H_5_CHCH	2	90	65
**j**	C_6_H_5_CH_2_	2	90	75
**k**	C_5_H_10_	2	90	80
**l**	isopropyl	8	100	95
**m**	*tert-*butyl	8	100	40

^a^Yields correspond to pure compounds.

### Mechanistic considerations

**Scheme 2:**
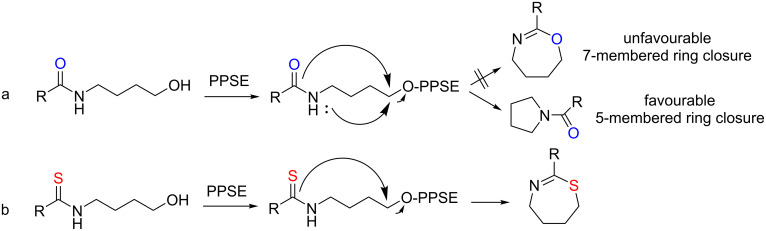
PPSE promoted ring closure reactions of amido- and thioamido alcohols.

The cyclization of 4-amidobutanols promoted by PPA esters leads exclusively to *N-*acylpyrrolidines instead of the corresponding tetrahydro-1,3-oxazepines. This unexpected reaction path is reasonable considering the relative ease of formation of five-membered rings as compared to the isomeric seven-membered heterocycles together with the relatively poor nucleophilicity of the carboxamide oxygen. An analogous effect was sometimes observed even in the sulfur analogues, as previously mentioned [32]. The differences between oxygen and sulfur regarding electronegativity and polarizability may explain the differential behaviour of the PPSE promoted ring closure of tetramethylenic amido and thioamido alcohols. In both cases, PPSE would activate the OH group for nucleophilic attack, and the plausible reaction mechanism would involve an intramolecular S_N_2-type displacement. However, the lower reactivity of the carboxamide oxygen (as an *O-*nucleophile), together with the comparatively high activation energy associated to the formation of a seven-membered heterocycle would favour the competitive ring closure leading to the five-membered ring (Scheme 2, reaction a), which would involve attack of the carboxamide nitrogen to the ω-carbon. On the other hand, the higher nucleophilicity of the sulfur in the thioamide would overcome the difficulty of the formation of the seven-membered ring, yielding exclusively the tetrahydrothiazepine (Scheme 2, reaction b).

## Conclusion

We have developed a general method for the synthesis of 2-aryltetrahydro-1,3-thiazepines, a heterocyclic core with very few examples in the literature. The method involves as the key step the microwave-assisted ring closure of thioamido alcohols promoted by PPSE, presumably via an intramolecular S_N_2-type displacement of the PPSE-activated OH group. The precursors were easily prepared in a three-step sequence from 4-aminobutanol in high overall yields. The whole synthetic procedure is expeditious and operationally simple, involving inexpensive and readily available starting materials and reagents. The cyclization step affords the desired heterocycles in good to high yields as the only products in a few minutes and is metal, additive and catalyst free. The scope of the method includes the synthesis of unprecedented examples of 2-alkyl, aralkyl and alkenyl derivatives.

## Supporting Information

File 1 Experimental procedures and characterization of new compounds.
