# Supplementary material for: Microwave-assisted synthesis of 2-substituted 4,5,6,7-tetrahydro-1,3-thiazepines from 4-aminobutanol
Source: Beilstein J Org Chem. 2020 Jan 6;16:32–8. doi: 10.3762/bjoc.16.5 (PMC6964664; doi:10.3762/bjoc.16.5)
Supplement: File 1 — Experimental procedures and characterization of new compounds. [file Beilstein_J_Org_Chem-16-32-s001.pdf]

BEILSTEIN JOURNAL OF ORGANIC CHEMISTRY

Supporting Information

for

Microwave-assisted synthesis of 2-substituted 4,5,6,7-tetrahydro-1,3-thiazepines from 4-aminobutanol

María C. Mollo, Natalia B. Kilimciler, Juan A. Bisceglia and Liliana R. Orelli

Beilstein J. Org. Chem. **2020,** *16,* 32–38. doi:10.3762/bjoc.16.5

Experimental procedures and characterization of new compounds

License and Terms: This is a supporting information file under the terms of the Creative Commons Attribution License (https://creativecommons.org/licenses/by/4.0). Please note that the reuse, redistribution and reproduction in particular requires that the authors and source are credited.
The license is subject to the *Beilstein Journal of Organic Chemistry* terms and conditions: (https://www.beilstein-journals.org/bjoc)

Table of contents

1. General information S2
2. Representative procedures for synthesis S2
3. Characterization data for compounds **1–4** S4
4. Copies of ^{1}H and ^{13}C NMR spectra of compounds **1–4** S18
5. References S71

1. General Information

Chromatography was carried out using Merck Kieselgel 60 (230–400 mesh). Thin layer chromatography was performed on Silica gel and was visualized by UV. Melting points were determined with a Büchi capillary apparatus and are uncorrected. ^{1}H and ^{13}C NMR spectra were recorded on a Bruker Bio Spin Avance III 600 MHz spectrometer or a Bruker Avance II 500 MHz spectrometer, using deuteriochloroform as the solvent. In ^{1}H NMR spectra, chemical shifts (ppm) are referenced to residual $CHCl_3$ (7.27 ppm in $CDCl_3$). In ^{13}C NMR spectra, chemical shifts (ppm) were referenced to the deuterated solvent (77.0 ppm in $CDCl_3$). D_2O was employed to confirm exchangeable protons (ex). Splitting multiplicities are reported as singlet (s), broad signal (bs), doublet (d), double doublet (dd), triplet (t), quartet (q), heptet (h) and multiplet (m). HRMS (ESI) were performed with a Bruker MicroTOF-Q II spectrometer. Reagents, solvents and starting materials were purchased from standard sources and purified according to literature procedures.

2. Representative procedures for synthesis

a. General procedure for the synthesis of *N*,*O*-diacyl-1,4-aminobutanols (1)

A solution of the acyl chloride or anhydride (5 mmol) in anhydrous dichloromethane (5 mL) was added dropwise to a mixture of 1,4-aminobutanol (2.5 mmol), DMAP (0.10 mmol) and 0.7 mL of triethylamine. The reaction mixture was stirred at room temperature until the disappearance of the acid chloride by TLC was observed. For compounds **1l,m** the reaction was carried out at reflux for 48 h. After the reaction was completed, dichloromethane was evaporated in vacuo. The crude product was purified by column chromatography (silicagel, hexane/ethyl acetate 3: 2→1:1).

b. General procedure for the synthesis of *N*-thioacyl-*O*-acyl-1,4-aminobutanols (2)

To a solution of amidoester **1** (2 mmol) in toluene (20 mL) was added LR (0.75 mmol). The mixture was heated at reflux for 30 min. After the reaction is complete, the toluene is evaporated in vacuo. The resulting residue is purified by column chromatography (silicagel, dichloromethane).

c. General procedure for the synthesis of *N*-(4-hydroxybutyl)thioamides (3)

Thioamidoester **2** (1.5 mmol) was placed in a round-bottomed flask and a solution of K_2CO_3 in water/methanol 1: 1 was added. The mixture was stirred at 70 °C for 30 minutes. After completion of the reaction, as indicated by TLC, the solvent is evaporated in vacuo. For compounds **3l,m** the reaction was carried out using 10% NaOH:methanol at reflux for 4h. The mixture obtained is diluted with water (15 mL) and extracted with dichloromethane (3 × 30 mL). The combined organic phases were washed with water, dried over anhydrous Na_2SO_4, filtered and concentrated in vacuo. The crude product was purified by column chromatography (silicagel, hexane: ethyl acetate 1: 1→2: 3)

d. General procedure for the synthesis of 4,5,6,7-tetrahydro-1,3-thiazepines (4)

A mixture of the corresponding compound **3** (1 mmol) and neat PPSE (6 g) was reacted in the microwave reactor (Monowave 300, Anton Paar) at the indicated temperature and time. After reaching room temperature, the resulting oil was treated with ethyl acetate (25 mL) and 10% aqueous NaOH (10 mL). The aqueous phase was extracted with ethyl acetate (2 × 25 mL). The organic phases were pooled, washed with water (5 mL), filtered, dried over Na_2SO_4 and filtered. The solvent was removed in vacuo. The crude products were purified by column chromatography (silicagel, hexane/ethyl acetate 3:2).

3. Characterization data for compounds 1–6

Compounds **1a,**[1] **1b,**[2] **1i,**[3] **3a,**[4] **3j,**[5] **4a,**[6] **4f** [7] were described in the literature.

4-Benzamidobutyl benzoate (1a) [1]

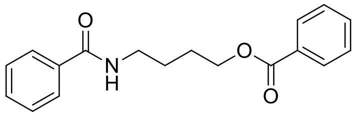

Prepared according to the general procedure, 706.25 mg, 95%. White solid, mp (hexane/$CHCl_3$): 42-44°C.

^{1}H NMR (600 MHz, $CDCl_3$) δ 1.71-1.74 (m, 2H), 1.79-1.84 (m, 2H), 3.47-3.50 (m, 2H), 4.31 (t, *J*=6.5 Hz, 2H), 6.86 (bs ex, 1H), 7.35-7.45 (m, 5H), 7.53 (t, *J*=7.5 Hz, 1H), 7.77 (d, *J*=7.2 Hz, 2H), 8.00 (d, *J*=7.2 Hz, 2H).

^{13}C NMR (151 MHz, $CDCl_3$) δ 26.1, 26.15, 39.5, 64.4, 126.8, 128.2, 128.3, 129.4, 130.0, 131.2, 132.8, 134.5, 166.5, 167.6.

HRMS (ESI): m/z Calcd. for $C_{18}H_{20}NO_3^+$ [M+H]$^+$: 298.1438; found: [M+H]$^+$: 298.1424.

4-(4-Chlorobenzamido)butyl 4-chlorobenzoate (1b) [2]

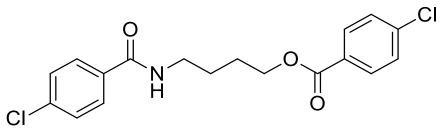

Prepared according to the general procedure, 824.0 mg, 90%. White solid, mp (ethyl acetate): 147-148°C.

^{1}H NMR (500 MHz, $CDCl_3$) δ 1.74-1.80 (m, 2H), 1.84-1.89 (m, 2H), 3.51-3.55 (m, 2H), 4.36 (t, *J*= 6.4 Hz, 2H), 6.26 (bs ex, 1H), 7.39-7.42 (m, 4H), 7.70 (ddd, *J*=8.7, 2.3, 2.0 Hz, 2H), 7.96 (ddd, *J*=8.7, 2.3, 2.0 Hz, 2H).

^{13}C NMR (125 MHz, $CDCl_3$) δ 26.27, 26.30, 39.7, 64.7, 128.3, 128.6, 128.7, 128.8, 130.9, 132.9, 137.7, 139.4, 165.8, 166.5.

HRMS (ESI): m/z Calcd. for $C_{18}H_{18}Cl_2NO_3^+$ [M+H]$^+$: 366.0658; found: [M+H]$^+$: 366.0662.

4-(4-Methylbenzamido)butyl 4-methylbenzoate (1c)

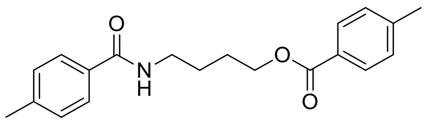

Prepared according to the general procedure, 756.0 mg, 93%. White solid, mp (hexane/ $CHCl_3$): 103-105°C.

^{1}H NMR (500 MHz, $CDCl_3$) δ 1.75-1.81 (m, 2H), 1.84-1.90 (m, 2H), 2.39 (s, 3H), 2.41 (s, 3H), 3.51-3.55 (m, 2H), 4.35 (t, *J*=6.4 Hz, 2H), 6.28 (bs ex, 1H), 7.21-7.24 (m, 4H), 7.67 (d, *J*=8.0 Hz , 2H), 7.91 (d, *J*= 8.0 Hz, 2H).
^{13}C NMR (125 MHz, $CDCl_3$) δ 21.4, 21.6, 26.3, 26.4, 39.6, 64.3, 126.8, 127.5, 129.1, 129.2, 129.5, 131.7, 141.7, 143.6, 166.7, 167.5
HRMS (ESI): m/z calcd. for $C_{20}H_{24}NO_3^+$ [M+H]$^+$: 326.1751; found: [M+H]$^+$: 326.1756.

4-(4-Methoxybenzamido)butyl 4-methoxybenzoate (1d)

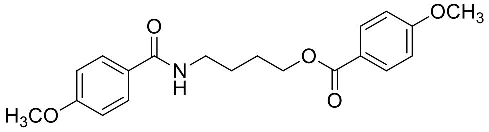

Prepared according to the general procedure, 849.0 mg, 95%. White solid, mp (isopropanol): 132-134°C.

^{1}H NMR(500 MHz, $CDCl_3$) δ 1.74-1.80 (m, 2H), 1.83-1.88 (m, 2H), 3.51-3.54 (m, 2H), 3.84 (s, 3H) ,3.86 (s, 3H), 4.32-4.35 (m, 2H), 6.29 (bs ex, 1H), 6.90-6.92 (m, 4H), 7.74 (ddd, *J*=8.9, 2.8, 2.1 Hz, 2H), 8.00 (ddd, *J*=8.9, 2.8, 2.1 Hz, 2H).

^{13}C NMR (125 MHz, $CDCl_3$) δ 26.38, 26.43, 39.6, 55.36, 55.40, 64.2, 113.6, 113.7, 122.6, 126.8, 128.6, 131.5, 162.1, 163.3, 166.4, 167.1.

HRMS (ESI): m/z calcd. for $C_{20}H_{24}NO_5^+$ $[M+H]^+$: 358.1649; found: $[M+H]^+$: 358.1655.

4-(4-Nitrobenzamido)butyl 4-nitrobenzoate (1e)

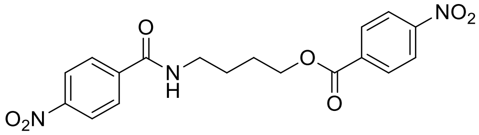

Prepared according to the general procedure, 871,5 mg, 90%. Yellow solid, mp (isopropanol): 130-131°C.

^{1}H NMR (500 MHz, $CDCl_3$) δ 1.80-1.86 (m, 2H), 1.90-1.96 (m, 2H), 3.58-3.62 (m, 2H), 4.46 (t, *J*=6.4 Hz, 2H), 6.33 (bs ex, 1H), 7.94 (d, *J*=8.7 Hz 2H), 8.22 (d, *J*= 8.9 Hz, 2H), 8.29-8.31 (m, 4H).

^{13}C NMR (125 MHz, $CDCl_3$) 26.1, 26.1, 39.9, 65.2, 123.5, 123.8, 128.1, 130.7, 135.5, 140.1, 149.5, 150.5, 164.7, 165.6.

HRMS (ESI): m/z Calcd. for $C_{18}H_{18}N_3O_7^+$ $[M+H]^+$: 388.1139; found: $[M+H]^+$: 388.1134.

4-(2,4-Dichlorobenzamido)butyl 2,4-dichlorobenzoate (1f)

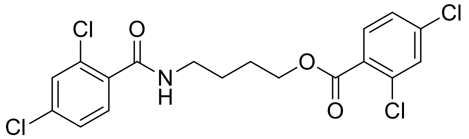

Prepared according to the general procedure, 1022,6 mg, 94%. White solid, mp (ethyl acetate): 100-102°C.

^{1}H NMR (500 MHz, $CDCl_3$) δ 1.77-1.82 (m, 2H), 1.86-1.91 (m, 2H), 3.51-3.55 (m, 2H), 4.38 (t, *J*=6.5 Hz, 2H), 6.36 (bs ex, 1H), 7.27-7.31 (m, 2H), 7.41 (d, *J*=2.0 Hz, 1H), 7.47 (d, *J*=2.0 Hz, 1H), 7.61 (d, *J*= 8.0 Hz, 1H), 7.79 (d, *J*=8.0 Hz, 1H) .

^{13}C NMR (125 MHz, $CDCl_3$) δ 26.1, 26.2, 39.7, 65.2, 127.0, 127.5, 128.4, 130.0, 131.0, 131.2, 131.3, 132.5, 133.4, 134.8, 136.7, 138.3, 164.8, 165.5.

HRMS (ESI): m/z calcd. for $C_{18}H_{16}Cl_4NO_3^+$ $[M+H]^+$: 433.9879; found: $[M+H]^+$: 433.9885 .

4-(2-Fluorobenzamido)butyl 2-fluorobenzoate (1g)

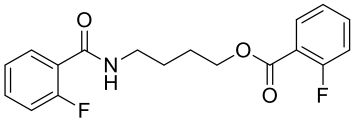

Prepared according to the general procedure, 800 mg, 96%. White solid, mp (ethyl acetate): 60-62°C

^{1}**H NMR** (500 MHz, $CDCl_3$) δ), 1.79-1.84 (m, 2H), 1.85-1.92 (m, 2H), 3.56-3.60 (m, 2H), 4.39-4.41 (m, 2H), 6.82 (bs ex, 1H), 7.10-7.16 (m, 2H), 7.20-7.23 (m, 1H), 7.25-7.28 (m, 1H), 7.45-7.49 (m, 1H), 7.50-7.55 (m,1H), 7.94 (td, *J*=7.5, 1.8 Hz, 1H), 8.10 (td, *J*=8.0, 1.8 Hz, 1H).

^{13}**C NMR** (125 MHz, $CDCl_3$) δ 26.1, 26.3, 39.6, 64.9, 115.9 (d, *J*= 25.4 Hz), 117.0 (d, *J*= 22.7 Hz), 118.7 (d, *J*= 9.1 Hz), 121.0 (d, *J*= 10.9 Hz),123.9 (d, *J*= 3.6 Hz), 124.8 (d, *J*= 2.7 Hz), 132.0 (d, *J*= 3.6 Hz), 132.1 (d, *J*= 2.7 Hz),133.2 (d, *J*= 9.1 Hz), 134.4 (d, *J*= 9.1 Hz), 160.5 (d, *J*= 247.1 Hz), 162.2 (d, *J*= 259.8 Hz), 163.3 (d, *J*= 2.7 Hz),164.5 (d, *J*= 3.6 Hz).

HRMS (ESI): m/z Calcd. for $C_{18}H_{18}F_2NO_3^+$ $[M+H]^+$: 334.1249; found: $[M+H]^+$: 334.1253.

4-(2-Methylbenzamido)butyl 2-methylbenzoate (1h)

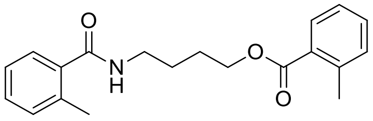

Prepared according to the general procedure, 732.2 mg, 90%. White solid, mp (hexane/ $CHCl_3$): 100-102°C.

^{1}**H NMR** (600 MHz, $CDCl_3$) δ 1.76-1.81 (m, 2H), 1.86-1.90 (m, 2H), 2.44 (s, 3H), 2.60 (s, 3H), 3.50-3.54 (m, 2H), 4.35 (t, *J*=6.4 Hz, 2H), 5.89 (bs ex, 1H), 7.18-7.25 (m, 4H), 7.31 (t, *J*=7.5 Hz, 1H), 7.34 (d, *J*=7.5 Hz, 1H), 7.40 (d, *J*= 7.5 Hz, 1H), 7.90 (d, *J*=8.0 Hz, 1H) .

^{13}**C NMR** (151 MHz, $CDCl_3$) δ 19.7, 21.7, 26.3, 26.5, 39.4, 64.2, 125.7, 126.5, 129.6, 129.8, 130.5, 131.0, 131.7, 132.0, 135.9, 136.5, 140.1, 167.6, 170.2.

HRMS (ESI): m/z calcd. for $C_{20}H_{24}NO_3^+$ $[M+H]^+$: 326.1751 ; found: $[M+H]^+$: 326.1745.

4-Cinnamamidobutyl cinnamate (1i) [3]

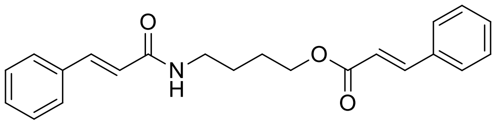

Prepared according to the general procedure, 830.0 mg, 95%. White solid, mp (hexane/ethyl acetate): 103-106°C.

^{1}**H NMR** (600 MHz, $CDCl_3$) δ 1.69-1.74 (m, 2H), 1.78-1.83 (m, 2H) , 3.45-3.49 (m, 2H), 4.25 (t, *J*=6.5 Hz, 2H), 5.90 (bs ex, 1H), 6.42 (d, *J*=15.6 Hz, 1H), 6.44 (d, *J*=16 Hz, 1H), 7.34-7.36 (m, 3H), 7.37-7.40 (m, 3H), 7.48-7.50 (m, 2H), 7.50-7.54 (m, 2H), 7.64 (d, *J*=15.6 Hz, 1H), 7.70 (d, *J*=16 Hz, 1H).

^{13}**C NMR** (151 MHz, $CDCl_3$) δ 26.3, 26.3, 39.3, 64.1, 118.0, 120.6, 127.7, 128.1, 128.8, 128.9, 129.6, 130.3, 134.3, 134.8, 141.0, 144.9, 165.9, 167.0.

HRMS (ESI): m/z calcd. for $C_{22}H_{24}NO_3^+$ $[M+H]^+$: 350.1751; found: $[M+H]^+$: 350.1755.

4-(2-Phenylacetamido)butyl 2-phenylacetate (1j)

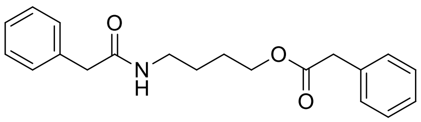

Prepared according to the general procedure, 700.0 mg, 86%. White solid, mp (hexane/ethyl acetate): 47-49°C.

^{1}H NMR (600 MHz, $CDCl_3$) δ 1.43−1.48 (m, 2H), 1.55-1.60 (m, 2H), 3.21 (c, *J* = 7.0 Hz, 2H), 3.58 (s, 2H), 3.61 (s, 2H), 4.07 (t, *J* = 6.5 Hz, 2H), 5.40 (bs ex, 1H), 7.26-7.28 (m, 5H), 7.31-7.34 (m, 3H), 7.37–7.39 (m, 2H).
^{13}C NMR (151 MHz, $CDCl_3$) δ 25.9, 26.0, 39.1, 41.4, 43.8, 64.3, 127.1, 127.4, 128.5, 129.0, 129.2, 129.4, 134.0, 134.9, 170.9, 171.5.

HRMS (ESI): m/z calcd. for $C_{20}H_{24}NO_3^+$ $[M+H]^+$: 326.1751; found: $[M+H]^+$: 326.1744.

4-Hexanamidobutyl hexanoate (1k)

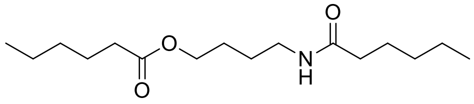

Prepared according to the general procedure, 499.5 mg, 70%. Colorless oil.
^{1}H NMR (600 MHz, $CDCl_3$) δ 0.85–0.88 (m, 6H), 1.25-1.33 (m, 8H), 1.52-1.66 (m, 8H), 2.14 (t, *J* = 7.7 Hz, 2H), 2.25 (t, *J* = 7.6 Hz, 2H), 3.24-3.27 (m, 2H), 4.05 (t, *J* = 6.5 Hz, 2H), 5.78 (bs ex, 1H).
^{13}C NMR (151 MHz, $CDCl_3$) δ 13.80, 13.83, 22.2, 22.3, 24.6, 25.4, 26.1, 26.2, 31.2, 31.4, 34.2, 36.7, 38.9, 63.7, 173.3, 173.9.

HRMS (ESI): m/z calcd. for $C_{16}H_{32}NO_3^+$ $[M+H]^+$: 286.2377; found: $[M+H]^+$: 286.2380.

4-Isobutyramidobutyl isobutyrate (1l)

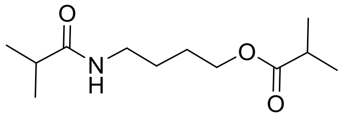

Prepared according to the general procedure, 533 mg, 93%. Yellow oil.
^{1}H NMR (600 MHz, $CDCl_3$) δ 1.14-1.16 (m, 12H), 1.54-1.59 (m, 2H) 1.63-1.68 (m, 2H), 2.33 (h, *J* = 6.5 Hz, 1H), 2.53 (h, *J* = 6.5 Hz,1H), 3.26-3.29 (m, 2H), 4.07 (t, *J* = 6.5 Hz, 2H), 5.62 (bs ex, 1H).
^{13}C NMR (151 MHz, $CDCl_3$) δ 18.9, 19.6, 26.1, 26.2, 34.0, 35.6, 38.9, 63.8, 177.0, 177.2.

HRMS (ESI): m/z calcd. for $C_{12}H_{24}NO_3^+$ $[M+H]^+$: 230.1751; found: $[M+H]^+$: 230.1744.

4-Pivalamidobutyl pivalate (1m)

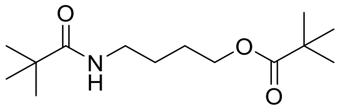

Prepared according to the general procedure, 533 mg, 77%. Yellow solid, mp (ethyl acetate): 38-40°C.
^{1}H NMR (600 MHz, $CDCl_3$) δ 1.19 (s, 18 H), 1.54-1.59 (m, 2H) 1.63-1.68 (m, 2H), 3.26-3.29 (m, 2H), 4.07 (t, *J* = 6.5 Hz, 2H), 5.70 (bs ex, 1H).
^{13}C NMR (151 MHz, $CDCl_3$) δ 26.1, 26.3, 27.2, 27.6, 38.6, 38.7, 39.1, 63.9, 178.4, 178.6.

HRMS (ESI): m/z calcd. for $C_{14}H_{28}NO_3$+ $[M+H]^+$: 258.2064; found: $[M+H]^+$: 258.2081.

4-Phenylthioamidobutyl benzoate (2a)

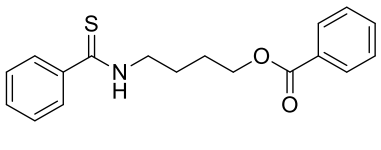

Prepared according to the general procedure, 595.4 mg, 95%. Yellow oil.

^{1}H NMR (600 MHz, $CDCl_3$) δ 1.90-1.97 (m, 4H), 3.92-3.95 (m, 2H), 4.40 (t, *J*=5.9 Hz, 2H), 7.36-7.39 (m, 2H), 7.43-7.46 (m, 3H), 7.57 (t, *J*=7.4 Hz, 1H), 7.73-7.74 (m, 3H), 8.05 (d, *J*=7.3 Hz, 2H).

^{13}C NMR (151 MHz, $CDCl_3$) δ 24.8, 26.4, 46.3, 64.3, 126.6, 128.4, 128.5, 129.5, 130.1, 131.0, 133.0, 141.9, 166.6, 199.5.

HRMS (ESI): m/z Calcd. for $C_{18}H_{20}NO_2S^+$ [M+H]+: 314.1209; found: $[M+H]^+$: 314.1215.

4-(4-Chlorophenylthioamido)butyl 4-chlorobenzoate (2b)

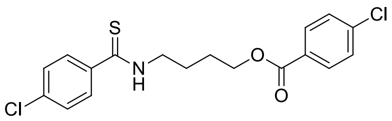

Prepared according to the general procedure, 711,0 mg, 93%. White solid, mp (ethyl acetate): 141-143°C.

^{1}H NMR (500 MHz, $CDCl_3$) δ 1.90-1.95 (m, 4H), 3.91-3.95 (m, 2H), 4.40 (t, *J*= 6.0 Hz, 2H), 7.36 (ddd, *J*=8.6, 2.6, 2.0 Hz, 2H), 7.42 (ddd, *J*=8.6, 2.3, 2.0 Hz, 2H), 7.66 (bs ex, 1H), 7.69 (ddd, *J*=8.6, 2.6, 2.0 Hz, 2H), 7.98 (ddd, *J*=8.6, 2.3, 2.0 Hz, 2H).

^{13}C NMR (125 MHz, $CDCl_3$) δ 24.8, 26.4, 46.4, 64.5, 127.9, 128.5, 128.7, 128.8, 131.0, 137.3, 139.6, 140.1, 165.8, 198.0.

HRMS (ESI): m/z Calcd. for $C_{18}H_{18}Cl_2NO_2S^+$ [M+H]+: 382.0430; found: $[M+H]^+$: 382.0425.

4-(4-Methylphenylthioamido)butyl 4-methylbenzoate (2c)

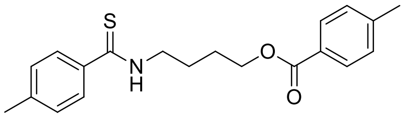

Prepared according to the general procedure, 648.8 mg, 95%. Yellow solid (Hexane/ $CHCl_3$), mp 108-110°C.
^{1}H NMR (500 MHz, $CDCl_3$) δ 1.88-1.97 (m, 4H), 2.37 (s, 3H), 2.42 (s, 3H), 3.91-3.95 (m, 2H), 4.38 (t, *J*= 6.0 Hz, 2H), 7.17-7.18 (m, 2H), 7.23-7.25 (m, 2H), 7.66 (d, *J*= 8.2 Hz, 2H) , 7.70 (bs ex, 1H), 7.93 (d, *J*= 8.2, 2H).
^{13}C NMR (125 MHz, $CDCl_3$) δ 21.3, 21.7, 24.9, 26.5, 46.3, 64.2, 126.6, 127.4, 129.11, 129.12, 129.6, 139.1, 141.6, 143.7, 166.7, 199.2.
HRMS (ESI): m/z Calcd. for $C_{20}H_{24}NO_2S^+$ $[M+H]^+$: 342.1522; found: $[M+H]^+$: 342.1530.

4-(4-Methoxyphenylthioamido)butyl 4-methoxybenzoate (2d)

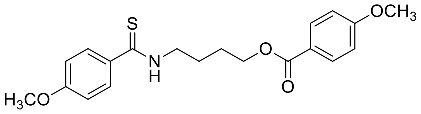

Prepared according to the general procedure, 694.7 mg, 93%. Yellow solid (ethyl acetate), mp 119-121°C.
^{1}H NMR (500 MHz, $CDCl_3$) δ 1.87-1.96 (m, 4H), 3.84 (s, 3H), 3.86 (s, 3H), 3.91-3.95 (m, 2H), 4.36 (t, *J*= 6.1 Hz, 2H), 6.87 (d, *J*= 8.9 Hz, 2H), 6.92 (d, *J*=8.9 Hz, 2H), 7.67 (bs ex, 1H), 7.76 (d, *J*= 8.9 Hz, 2H), 7.99 (d, *J*= 8.9, 2H).
^{13}C NMR (125 MHz, $CDCl_3$) δ 24.9, 26.5, 46.3, 55.42, 55.45, 64.0, 113.6, 113.6, 122.5, 128.4, 131.6, 134.1, 162.1, 163.4, 166.4, 198.2.
HRMS (ESI): m/z Calcd. for $C_{20}H_{24}NO_4S^+$ $[M+H]^+$: 374.1421; found: $[M+H]^+$: 374.1425.

4-(4-Nitrophenylthioamido)butyl 4-nitrobenzoate (2e)

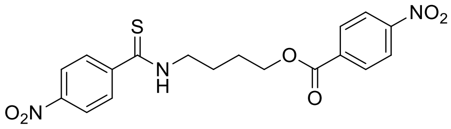

Prepared according to the general procedure, 807,0 mg, 100%. Yellow solid, mp (isopropanol): 133-135°C

^{1}H NMR (600 MHz, $CDCl_3$) δ 1.97-2.01 (m, 4H), 3.95-3.97 (m, 2H), 4.47 (t, *J*=6.0 Hz, 2H), 7.83 (bs ex, 1H), 7.86 (d, *J*=8.6 Hz 2H), 8.21-8.23 (m, 4H), 8.29 (d, *J*= 8.7 Hz, 2H).
^{13}C NMR (151 MHz, $CDCl_3$) 24.6, 26.2, 46.4, 65.1, 123.6, 123.7, 127.6, 130.7, 135.4, 146.9, 148.9, 150.6, 164.7, 197.0.
HRMS (ESI): m/z Calcd. for $C_{18}H_{18}N_3O_6S^+$ [M+H]+: 404.0911; found: $[M+H]^+$: 404.0905.

4-(2,4-Dichlorophenylthioamido)butyl 2,4-dichlorobenzoate (2f)

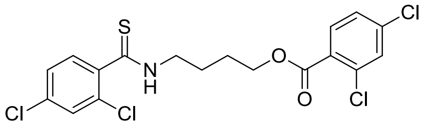

Prepared according to the general procedure, 785.0 mg, 87%. Yellow solid, mp (ethyl acetate): 134-136°C

^{1}H NMR (600 MHz, $CDCl_3$) δ 1.90-1.98 (m, 4H), 3.89-3.92 (m, 2H), 4.41 (t, *J*=5.7 Hz, 2H), 7.26 (dd, *J*=8.4, 2.0 Hz, 1H), 7.31 (dd, *J*=8.4, 2.0 Hz, 1H), 7.37 (d, *J*=2.0 Hz, 1H), 7.47 (d, *J*=2.0 Hz, 1H), 7.51 (d, *J*=8.4 Hz, 1H), 7.58 (bs ex, 1H), 7.80 (d, *J*=8.4 Hz, 1H).
^{13}C NMR (151 MHz, $CDCl_3$) δ 24.6, 26.2, 45.9, 65.1, 127.1, 127.4, 128.3, 129.1, 129.6, 131.0, 131.1, 132.5, 134.7, 135.7, 138.4, 140.3, 164.9, 196.1
HRMS (ESI): m/z Calcd. for $C_{18}H_{16}Cl_4NO_2S^+$ $[M+H]^+$: 449.9650; found: $[M+H]^+$: 449.9656.

4-(2-Fluorophenylthioamido)butyl 2-fluorobenzoate (2g)

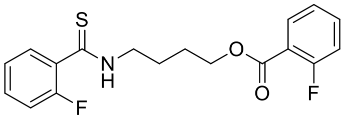

Prepared according to the general procedure, 629.0 mg, 90%. Yellow oil.

^{1}H NMR (500 MHz, $CDCl_3$) δ 1.91-1.98 (m, 4H), 3.94-3.97 (m, 2H), 4.41 (d, *J*= 6 Hz, 2H), 7.03-7.08 (m, 1H), 7.18-7.23 (m, 1H), 7.37-7.40 (m, 1H), 7.50-7.5m (m, 1H), 7.94 (td, *J*=7.5, 1.8 Hz, 1H), 7.96 (bs ex, 1H), 8.10 (td, *J*=8.0, 1.9 Hz, 1H).

^{13}C NMR (125 MHz, $CDCl_3$) δ 24.7, 26.1, 46.4, 64.7, 115.8 (d, *J*= 23.6 Hz), 116.9 (d, *J*= 22.5 Hz), 118.7 (d, *J*= 10.0 Hz) , 124.0 (d, *J*= 3.6 Hz),124.5 (d, *J*= 3.6 Hz), 128.1 (d, *J*= 10.7 Hz), 132.1, 132.2 (d, *J*= 9.0 Hz),133.3 (d, *J*= 1.6 Hz), 134.5 (d, *J*= 9.0 Hz), 157.6 (d, *J*= 248.4 Hz), 161.9 (d, *J*= 259.6 Hz), 164.5 (d, *J*= 3.6 Hz), 193.6 (d, *J*= 1.7 Hz).

HRMS (ESI): m/z Calcd. for $C_{18}H_{18}F_2NO_2S^+$ [M+H]+: 350.1021; found: $[M+H]^+$: 350.1014.

4-(2-Methylphenylthioamido)butyl 2-methylbenzoate (2h)

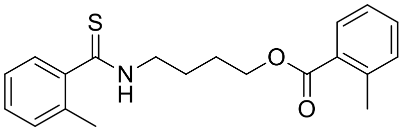

Prepared according to the general procedure, 628.0 mg, 92%. White solid (Hexane/ $CHCl_3$), mp 91-93°C.
^{1}H NMR (500 MHz, $CDCl_3$) δ 1.90-1.93 (m, 4H), 2.37 (s, 3H), 2.59 (s, 3H), 3.88-3.92 (m, 2H), 4.35-4.37 (m, 2H), 7.17-7.20 (m, 2H), 7.23-7.27 (m, 4H), 7.41 (td, *J*= 7.4, 1,2 Hz, 2H) , 7.46 (bs ex, 1H), 7.90 (d, *J*= 7.4, 2H).
^{13}C NMR (125 MHz, $CDCl_3$) δ 19.3, 21.7, 24.9, 26.4, 45.4, 64.0, 125.7, 125.9, 126.5, 128.9, 129.5, 130.5, 130.7, 131.7, 132.0, 132.8, 140.1, 143.9, 167.5, 201.9.
HRMS (ESI): m/z Calcd. for $C_{20}H_{24}NO_2S^+$ [M+H]+: 342.1522; found: $[M+H]^+$: 342.1525.

4-((*E*)-3-Phenylprop-2-enethioamido)butyl cinnamate (2i)

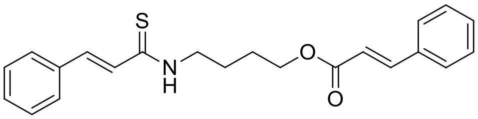

Prepared according to the general procedure, 694.4 mg, 95%. White solid, mp (hexane/ethyl acetate): 122-125°C.

This compound was obtained as inseparable mixture of E/Z diasteroisomers.[8] Only the major isomer is reported.

^{1}H NMR (600 MHz, $CDCl_3$) δ 1.81-1.90 (m, 4H), 3.89-3.92 (m, 2H), 4.29 (t, *J*=6.0 Hz, 2H), 6.47 (d, *J*=16.0, 1H), 6.86 (d, *J*=15.3 Hz, 1H), 7.35-7.37 (m, 3H), 7.39-7.41 (m, 3H), 7.52-7.57 (m, 4H), 7.57 (bs ex, 1H), 7.71 (d, *J*=16 Hz, 1H), 7.82 (d, *J*=15.3 Hz, 1H).

^{13}C NMR (151 MHz, $CDCl_3$) δ 24.8, 26.5, 45.6, 63.9, 117.8, 127.6, 128.0, 128.1, 128.85, 128.89, 129.8, 130.4, 134.3, 134.9, 141.6, 145.1, 167.1, 194.9.

HRMS (ESI): m/z Calcd. for $C_{22}H_{24}NO_2S^+$ [M+H]+: 366.1522; found: $[M+H]^+$: 366.1518.

4-(2-Phenylethanethioamido)butyl 2-phenylacetate (2j)

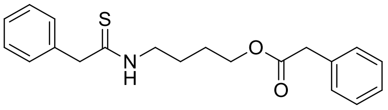

Prepared according to the general procedure, 546.0 mg, 80%. Yellow oil.
^{1}H NMR (600 MHz, $CDCl_3$) δ 1.55–1.57 (m, 4H), 3.60-3.62 (m, 4H)*, 4.06 (t, *J* = 6.0 Hz, 2H), 4.13 (s, 2H), 6.99 (bs ex, 1H), 7.25-7.27 (m, 5H), 7.30-7.40 (m, 5H).
^{13}C NMR (151 MHz, $CDCl_3$) δ 24.3, 25.9, 41.4, 45.5, 53.2, 64.1, 127.1, 127.9, 128.6, 129.2, 129.3, 129.5, 134.0, 134.8, 171.5, 202.1.

HRMS (ESI): m/z Calcd. for $C_{20}H_{24}NO_2S^+$ $[M+H]^+$: 342.1522; found: $[M+H]^+$: 342.1527.
*overlapping signals

4-Hexanethioamidobutyl hexanoate (2k)

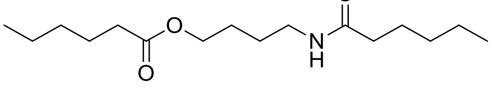

Prepared according to the general procedure, 603.0 mg, 100%. Yellow oil.

^{1}H NMR (600 MHz, $CDCl_3$) δ 0.88–0.91 (m, 6H), 1.27-1.38 (m, 8H), 1.60-1.65 (m, 2H), 1.70-1.79 (m, 6H), 2.30 (t, *J* = 7.6 Hz, 2H), 2.64 (t, *J* = 7.7 Hz, 2H), 3.69-3.73 (m, 2H), 4.11 (t, *J* = 6.0 Hz, 2H), 7.37 (bs ex, 1H).

^{13}C NMR (151 MHz, $CDCl_3$) δ 13.87, 13.90, 22.3, 22.4, 24.5, 24.6, 26.2, 29.1, 31.1, 31.3, 34.3, 45.5, 47.3, 63.5, 174.0, 205.9.

HRMS (ESI): m/z Calcd. for $C_{16}H_{32}NO_2S^+$ [M+H]$^+$: 302.2148; found: [M+H]$^+$: 302.2154.

4-(2-Methylpropanethioamido)butyl isobutyrate (2l)

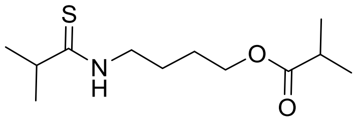

Prepared according to the general procedure, 417.0 mg, 85%. Yellow oil.

^{1}H NMR (600 MHz, $CDCl_3$) δ 1.15 (d, *J* = 7.0 Hz, 6H), 1.25 (d, *J* = 6.6 Hz, 6H), 1.70-1.75 (m, 4H), 2.54 (h, *J* = 7.0 Hz, 1H), 2.80 (h, *J* = 6.6 Hz,1H), 3.70-3.73 (m, 2H), 4.09-4.11 (m, 2H), 7.40 (bs ex, 1H).

^{13}C NMR (151 MHz, $CDCl_3$) δ 18.9, 22.6, 24.5, 26.2, 34.0, 44.5, 45.2, 63.6, 177.2, 211.7.

HRMS (ESI): m/z calcd. for $C_{12}H_{24}NO_2S^+$ [M+H]$^+$: 246.1522; found: [M+H]$^+$: 246.1526.

4-(2,2-Dimethylpropanethioamido)butyl pivalate (2m)

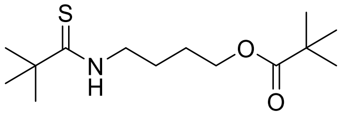

Prepared according to the general procedure, 475 mg, 87%. White solid, mp (Hexane/ $CHCl_3$): 35-47°C.

^{1}H NMR (600 MHz, $CDCl_3$) δ 1.20 (s, 9 H), 1.35 (s, 9 H), 1.69-1.73 (m, 4H), 3.71-3.74 (m, 2H), 4.10 (t, *J* = 6.2 Hz, 2H), 5.70 (bs ex, 1H).

^{13}C NMR (151 MHz, $CDCl_3$) δ 24.5, 26.2, 27.2, 30.1, 38.7, 44.5, 45.8, 63.6, 178.6, 213.46.

HRMS (ESI): m/z calcd. for $C_{14}H_{28}NO_2S^+$ [M+H]$^+$: 274.1835; found: [M+H]$^+$: 274.1841.

***N*-(4-Hydroxybutyl)benzothioamide (3a)**[4]

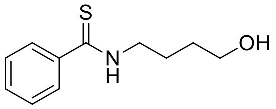

Prepared according to the general procedure, 298.0 mg, 95%. White solid (Hexane/ $CHCl_3$), mp 61-62°C.

^{1}H NMR (600 MHz, $CDCl_3$) δ 1.70-1.74 (m, 2H), 1.82 (bs ex, 1H), 1.86-1.91 (m, 2H), 3.74 (t, *J*= 6.0 Hz, 2H), 3.83-3.87 (m, 2H), 7.36-7.38 (m, 2H), 7.43-7.46 (m, 1H), 7.75 (d, *J*= 7.3 Hz, 2H) , 8.32 (bs ex, 1H).

4-Chloro-*N*-(4-hydroxybutyl)benzothioamide (3b)

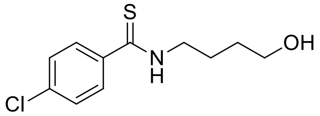

Prepared according to the general procedure, 318.0 mg, 87%. White solid (Hexane/ $CHCl_3$), mp 89-90°C.

^{1}H NMR (500 MHz, $CDCl_3$) δ 1.69-1.74 (m, 2H), 1.86-1.91 (m, 2H), 1.97 (bs ex, 1H), 3.73 (t, *J*= 6.0 Hz, 2H), 3.79-3.83 (m, 2H), 7.33 (ddd, *J*=8.6, 2.6, 2.0 Hz, 2H), 7.70 (ddd, *J*=8.6, 2.6, 2.0 Hz, 2H), 8.50 (bs ex, 1H).
^{13}C NMR (125 MHz, $CDCl_3$) δ 24.7, 29.6, 46.9, 62.2, 128.1, 128.5, 137.1, 139.9, 197.2
HRMS (ESI): m/z Calcd. for $C_{11}H_{15}ClNOS$+ $[M+H]^+$: 244.0557; found: $[M+H]^+$: 244.0561.

***N*-(4-Hydroxybutyl)-4-methylbenzothioamide (3c)**

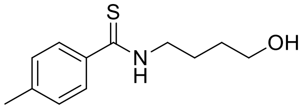

Prepared according to the general procedure, 274.0 mg, 82%. Yellow oil.
^{1}H NMR (500 MHz, $CDCl_3$) δ 1.71-1.76 (m, 2H), 1.90-1.93 (m, 2H), 2.38 (s, 3H), 3.76 (t, *J*= 6.0 Hz, 2H), 3.85-3.88 (m, 2H), 7.18 (d, *J*=8.1 Hz, 2H), 7.68 (d, *J*=8.1 Hz, 2H), 8.20 (bs ex, 1H).
^{13}C NMR (125 MHz, $CDCl_3$) δ 21.3, 24.7, 29.7, 46.6, 62.3, 126.7, 129.1, 139.0, 141.5, 198.7.
HRMS (ESI): m/z Calcd. for $C_{12}H_{18}NOS^+$ $[M+H]^+$: 224.1104; found: $[M+H]^+$: 224.1111.

***N*-(4-Hydroxybutyl)-4-methoxybenzothioamide (3d)**

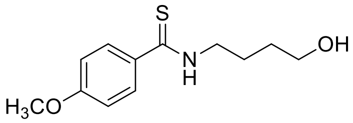

Prepared according to the general procedure, 312.0 mg, 87%. Yellow oil.
^{1}H NMR (500 MHz, $CDCl_3$) δ 1.70-1.75 (m, 2H), 1.85 (bs ex, 1H), 1.86-1.92 (m, 2H), 3.75 (m, *J*= 6.0 Hz, 2H), 3.84-3.87 (m, 5H), 6.87 (ddd, *J*=8.8, 3.1, 2.0 Hz, 2H), 7.78 (ddd, *J*=8.8, 3.1, 2.0 Hz, 2H), 8.19 (bs ex, 1H).
^{13}C NMR (125 MHz, $CDCl_3$) δ 24.7, 29.7, 46.6, 55.4, 62.3, 113.5, 128.5, 134.1, 162.0, 197.7.
HRMS (ESI): m/z Calcd. for $C_{12}H_{18}NO_2S^+$ $[M+H]^+$: 240.1053; found: $[M+H]^+$: 240.1059.
*Overlapping signals

***N*-(4-Hydroxybutyl)-4-nitrobenzothioamide (3e)**

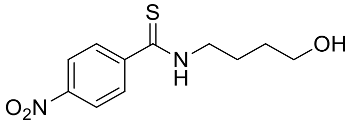

Prepared according to the general procedure, 347.3 mg, 91%. Yellow oil.
^{1}H NMR (500 MHz, $CDCl_3$) δ 1.70 (bs ex, 1H) 1.76-1.81 (m, 2H), 1.93-1.98 (m, 2H), 3.80 (t, *J*= 5.8 Hz, 2H), 3.84-3.87 (m, 2H), 7.91 (ddd, *J*=8.6, 2.5, 1.9 Hz, 2H), 8.22 (ddd, *J*=8.6, 2.5, 1.9 Hz, 2H), 8.76 (bs ex, 1H).
^{13}C NMR (125 MHz, $CDCl_3$) δ 24.7, 29.5, 47.2, 62.4, 123.6, 127.8, 146.9, 148.8, 196.0.
HRMS (ESI): m/z Calcd. for $C_{11}H_{15}N_2O_3S^+$ $[M+H]^+$: 255.0798; found: $[M+H]^+$: 255.0793.

2,4-Dichloro-*N*-(4-hydroxybutyl)benzothioamide (3f)

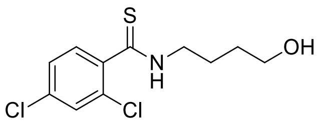

Prepared according to the general procedure, 408.9 mg, 98%. Yellow oil.
^{1}H NMR (600 MHz, $CDCl_3$) δ 1.69-1.74 (m, 3H)*, 1.86-1.90 (m, 2H), 3.72 (t, *J*= 6.0 Hz, 2H), 3.82-3.85 (m, 2H), 7.26 (dd, *J*=8.3, 2 Hz; 1H), 7.38 (d, *J*=8.3 Hz, 1H), 7.49 (d, *J*=2.0 Hz, 1H), 8.11 (bs ex, 1H).

^{13}**C NMR** (151 MHz, $CDCl_3$) δ 24.5, 29.7, 46.3, 62.2, 127.3, 129.2, 129.6, 130.9, 135.5, 140.5, 195.6.
HRMS (ESI): m/z Calcd. for $C_{11}H_{14}Cl_2NOS^+$ $[M+H]^+$: 278.0168; found: $[M+H]^+$: 278.0164.
*overlapping signals

2-Fluoro-*N*-(4-hydroxybutyl)benzothioamide (3g)

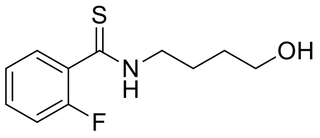

Prepared according to the general procedure, 307 mg, 90%. Pale yellow oil.

^{1}**H NMR** (500 MHz, $CDCl_3$) δ 1.69-1.74 (m, 3H)*, 1.86-1.92 (m, 2H), 3.74 (t, *J*=6.1 Hz, 2H), 3.88-3.91 (m, 2H), 7.05-7.09 (m, 1H), 7.18-7.22 (m, 1H), 7.37-7.42 (m, 1H), 8.08 (td, *J*=8.0, 1.8 Hz, 1H), 8.33 (bs ex, 1H).

^{13}**C NMR** (125 MHz, $CDCl_3$) δ 24.5, 29.6, 46.7, 62.2, 115.8 (d, J=23.5 Hz), 124.5 (d, *J*=3.3 Hz), 128.4 (d, *J*=10.9 Hz), 132.2 (d, *J*=9 Hz), 133.2 (d, *J*=1.6 Hz), 156.6 (d, *J*=248.6 Hz), 193.3.

HRMS (ESI): m/z Calcd. for $C_{11}H_{15}FNOS^+$ $[M+H]^+$: 228.0853; found: $[M+H]^+$: 228.0856.
*overlapping signals

***N*-(4-Hydroxybutyl)-2-methylbenzothioamide (3h)**

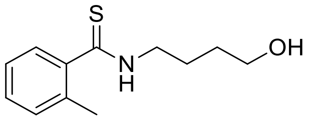

Prepared according to the general procedure, 278 mg, 83%. Yellow oil.
^{1}**H NMR** (500 MHz, $CDCl_3$) δ 1.68-1.74 (m, 2H), 1.84-1.90 (m, 2H), 2.38 (s, 3H), 3.73 (t, *J*= 6.0 Hz, 2H), 3.83-3.87 (m, 2H), 7.18 (d, *J*=7.5, 2H), 7.23-7.27 (m, 3H), 7.76 (bs ex, 1H).
^{13}**C NMR** (125 MHz, $CDCl_3$) δ 19.4, 24.6, 29.7, 45.8, 62.20, 126.0, 126.5, 128.9, 130.7, 132.9, 144.0, 201.5.
HRMS (ESI): m/z Calcd. for $C_{12}H_{18}NOS^+$ $[M+H]^+$: 224,1104; found: $[M+H]^+$: 224,1099.

(*E*)-*N*-(4-Hydroxybutyl)-3-phenylprop-2-enethioamide (3i)

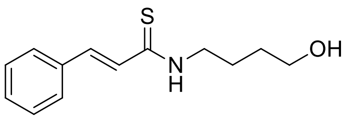

Prepared according to the general procedure, 282 mg, 80%. Yellow solid (Hexane/ $CHCl_3$), mp 80-83°C

This compound was obtained as inseparable mixture of E/Z diasteroisomers.[8] Only the major isomer is reported.

^{1}**H NMR** (600 MHz, $CDCl_3$) δ 1.68-1.72 (m, 2H), 1.83-1.88 (m, 3H)*, 3.74 (t, *J*=6 Hz, 2H), 3.81-3.84 (m, 2H), 6.84 (d, *J*=16 Hz, 1H), 7.34-7.37 (m, 4H), 7.52-7.53 (m, 2H), 7.80 (d, *J*=16 Hz, 1H), 8.09 (bs ex, 1H).
^{13}**C NMR** (151 MHz, $CDCl_3$) δ 24.8, 29.7, 45.8, 62.2, 127.7, 128.0, 128.8, 129.7, 134.9, 141.3, 194.3.

HRMS (ESI): m/z Calcd. for $C_{13}H_{18}NOS^+$ $[M+H]^+$: 236.1104; found: $[M+H]^+$: 236.1110.
*overlapping signals

***N*-(4-Hydroxybutyl)-2-phenylethanethioamide (3j)**[5]

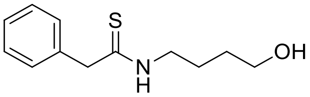

Prepared according to the general procedure, 268 mg, 80%. Yellow oil.

^{1}H NMR (600 MHz, $CDCl_3$) δ 1.47 (bs ex, 1H), 1.51-1.55 (m, 2H), 1.65-1.69 (m, 2H), 3.58 (t, *J*= 6.0 Hz, 2H), 3.64-3.67 (m, 2H), 4.13 (s, 2H), 7.26 (d, *J*=7.1 Hz, 2H), 7.32-7.34 (m, 1H), 7.37-7.39 (m, 2H), 7.56 (bs ex, 1H).

***N*-(4-Hydroxybutyl)hexanethioamide (3k)**

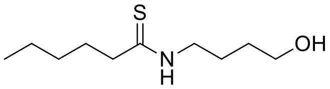

Prepared according to the general procedure, 284.0 mg, 93%. Yellow oil.

^{1}H NMR (600 MHz, $CDCl_3$) δ 0.88 (t, *J*=7.1 Hz, 2H), 1.26-1.34 (m, 4H), 1.63-1.67 (m, 2H), 1.73-1.80 (m, 4H), 2.27 (bs ex, 1H) 2.62 (t, *J*=7.7 Hz, 2H), 2.78-2.79 (m, 2H), 3.65-3.69 (m, 2H), 3.70 (t, *J*=6.0 Hz, 2H), 8.00 (bs ex, 1H).

^{13}C NMR (151 MHz, $CDCl_3$) δ 13.9, 22.3, 24.5, 29.0, 29.5, 31.0, 45.8, 47.1, 62.1, 205.4.

HRMS (ESI): m/z Calcd. for $C_{10}H_{22}NOS^+$ $[M+H]^+$: 204.1417; found: $[M+H]^+$: 204.1421.

***N*-(4-Hydroxybutyl)-2-methylpropanethioamide (3l)**

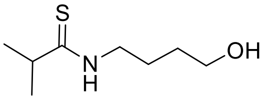

Prepared according to the general procedure, 187mg, 71%. White solid (hexane/$CHCl_3$ crystallized): 44-46°C.

^{1}H NMR (600 MHz, $CDCl_3$) δ 1.22 (d, *J*=6.7 Hz, 6H), 1.62-1.66 (m, 2H), 1.75-1.80 (m, 2H), 2.46 (bs ex, 1H), 2.81 (h, *J*=6.7 Hz, 1H), 3.65-3.70 (m, 4H), 8.04 (bs ex, 1H).

^{13}C NMR (151 MHz, $CDCl_3$) δ 22.4, 24.4, 29.5, 44.3, 45.5, 62.0, 211.1.

HRMS (ESI): m/z Calcd. for $C_8H_{18}NOS^+$ $[M+H]^+$: 176.1104; found: $[M+H]^+$: 176.1106.

***N*-(4-Hydroxybutyl)-2,2-dimethylpropanethioamide (3m)**

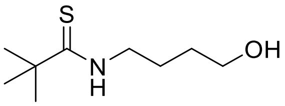

Prepared according to the general procedure, 252 mg, 89%. Yellow oil.

^{1}H NMR (600 MHz, $CDCl_3$) δ 1.34 (s, 9H), 1.63-1.67 (m, 2H), 1.77-1.82 (m, 2H), 2.09 (bs ex, 1H), 3.68-3.72 (m, 4H), 7.88 (bs ex, 1H).

^{13}C NMR (151 MHz, $CDCl_3$) δ 24.3, 29.5, 30.0, 44.4, 46.2, 62.0, 213.1.

HRMS (ESI): m/z Calcd. for $C_9H_{20}NOS^+$ $[M+H]^+$: 190.1260; found: $[M+H]^+$: 190.1251.

2-Phenyl-4,5,6,7-tetrahydro-1,3-thiazepine (4a) [6]

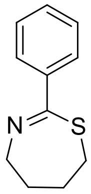

Prepared according to the general procedure, 139.0 mg, 73%. Colorless oil.

^{1}H NMR (600 MHz, $CDCl_3$) δ 1.89-1.92 (m, 2H), 2.07-2.11 (m, 2H), 2.91-2.93 (m, 2H), 4.07-4.09 (m, 2H), 7.37-7.39 (m, 2H), 7.43 (t, *J* = 7.3 Hz, 1H), 7.95 (d, *J* = 7.8 Hz, 2H).

^{13}C NMR (151 MHz, $CDCl_3$) δ 25.6, 28.0, 30.9, 53.8, 128.1, 128.5, 130.5, 139.8, 163.8.

HRMS (ESI): m/z calcd. for $C_{11}H_{14}NS^+$ $[M+H]^+$: 192.0841; found: 192.0845.

2-(4-Chlorophenyl)-4,5,6,7-tetrahydro-1,3-thiazepine (4b)

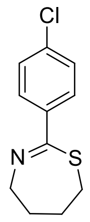

Prepared according to the general procedure, 185 mg, 82%. Yellow oil.

^{1}H NMR (500 MHz, $CDCl_3$) δ 1.87-1.92 (m, 2H), 2.06-2.11 (m, 2H), 2.91-2.93 (m, 2H), 4.05-4.07 (m, 2H), 7.35 (ddd, *J*=8.6, 2.5, 1.8 Hz, 2H), 7.91 (ddd, *J*=8.6, 2.5, 1.8 Hz, 2H).

^{13}C NMR (125 MHz, $CDCl_3$) δ 25.5, 28.0, 31.0, 53.8, 128.3, 129.8, 136.8, 138.0, 163.1.

HRMS (ESI): m/z calcd. for $C_{11}H_{13}ClNS^+$ $[M+H]^+$: 226.0452; found: 226.0448.

2-(4-Methylphenyl)-4,5,6,7-tetrahydro-1,3-thiazepine (4c)

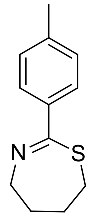

Prepared according to the general procedure, 147.8 mg, 72%. Yellow oil.

^{1}H NMR (300 MHz, $CDCl_3$) δ 1.85-1.93 (m, 2H), 2.04-2.11 (m, 2H), 2.38 (s, 3H), 2.88-2.92 (m, 2H), 4.03-4.07 (m, 2H), 7.18 (d, *J*=8.1 Hz, 2H), 7.85 (ddd, *J*=8.1 Hz, 2H).

^{13}C NMR (75 MHz, $CDCl_3$) δ 21.3, 25.7, 28.0, 30.9, 53.7, 128.5, 128.8, 137.1, 140.8, 163.7.

HRMS (ESI): m/z calcd. for $C_{12}H_{16}NS^+$ $[M+H]^+$: 206.0998; found: 206.0992.

2-(4-Methoxyphenyl)-4,5,6,7-tetrahydro-1,3-thiazepine (4d)

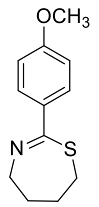

Prepared according to the general procedure, 177.1 mg, 80%. Colorless oil.

^{1}H NMR (500 MHz, $CDCl_3$) δ 1.85-1.89 (m, 2H), 2.03-2.08 (m, 2H), 2.87-2.89 (m, 2H), 3.83 (s, 3H), 4.01-4.03 (m, 2H), 6.88 (ddd, *J*=8.9, 2.9, 2.0 Hz, 2H), 7.91 (ddd, *J*=8.9, 2.9, 2.0 Hz, 2H).

^{13}C NMR (125 MHz, $CDCl_3$) δ 25.8, 28.0, 30.9, 53.6, 55.3, 113.3, 130.1, 132.4, 161.6, 163.2.

HRMS (ESI): m/z calcd. for $C_{12}H_{16}NOS^+$ $[M+H]^+$: 222.0947; found: 222.0952.

2-(4-Nitrophenyl)-4,5,6,7-tetrahydro-1,3-thiazepine (4e)

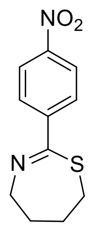

Prepared according to the general procedure, 153.6 mg, 65%. Yellow solid, mp (hexane/$CHCl_3$ crystallized): 89-91°C.

^{1}H NMR (500 MHz, $CDCl_3$) δ 1.90-1.96 (m, 2H), 2.09-2.14 (m, 2H), 2.95-2.97 (m, 2H), 4.11-4.14 (m, 2H), 8.12 (ddd, *J*=9.0, 2.3, 2.0 Hz, 2H), 8.22 (ddd, *J*=9.0, 2.3, 2.0 Hz, 2H).

^{13}C NMR (125 MHz, $CDCl_3$) δ 25.3, 28.1, 31.0, 54.1, 123.3, 129.3, 145.1, 149.0, 162.2.

HRMS (ESI): m/z calcd. for $C_{11}H_{13}N_2O_2S^+$ $[M+H]^+$: 237.0692; found: 237.0700.

2-(2,4-Dichlorophenyl)-4,5,6,7-tetrahydro-1,3-thiazepine (4f)[7]

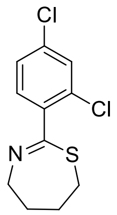

Prepared according to the general procedure, 182.0 mg, 70%. Pale yellow oil.

^{1}H NMR (500 MHz, $CDCl_3$) δ 1.90-1.94 (m, 2H), 2.16-2.20 (m, 2H), 2.94-2.96 (t, 2H), 4.04-4.06 (m, 2H), 7.23 (dd, *J*=8.3, 1.5 Hz; 1H), 7.26 (d, *J*=8.3 Hz, 1H), 7.39 (d, *J*=1.5 Hz, 1H).

^{13}C NMR (125 MHz,$CDCl_3$) δ 25.6, 28.3, 31.8, 53.5, 127.0, 129.7, 130.4, 132.8, 135.2, 138.6, 161.5.

HRMS (ESI): m/z calcd. for $C_{11}H_{12}Cl_2NS^+$ $[M+H]^+$: 260.0062; found: 260.0058.

2-(2-Fluorophenyl)-4,5,6,7-tetrahydro-1,3-thiazepine (4g)

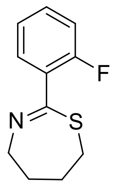

Prepared according to the general procedure, 134.0 mg, 64%. Pale yellow oil.

^{1}H NMR (500 MHz, $CDCl_3$) δ 1.91-1.95 (m, 2H), 2.14-2.18 (m, 2H) , 2.95-2.97 (m, 2H), 4.09-4.11 (m, 2H), 7.07-7.10 (m, 1H), 7.13-7.15 (m, 1H), 7.33-7.37 (m, 1H), 7.52 (td, *J*=7.5, 1.7 Hz, 1H).

^{13}C NMR (125 MHz, $CDCl_3$) δ 25.5, 28.1, 31.4, 53.4, 116.1 (d, J=22.0 Hz), 123.8 (d, *J*=3.7 Hz), 129.0 (d, *J*=10.5 Hz), 130.3 (d, *J*=2.4 Hz), 131.1 (d, *J*=8.5 Hz), 159.8 (d, *J*=251.6 Hz), 160.4.

HRMS (ESI): m/z calcd. for $C_{11}H_{13}FNS^+$ $[M+H]^+$: 210.0747; found: 210.0750.

2-(2-Methylphenyl)-4,5,6,7-tetrahydro-1,3-thiazepine (4h)

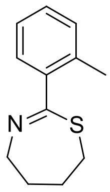

Prepared according to the general procedure, 133.5 mg, 65%. Colorless oil.

^{1}H NMR (500 MHz, $CDCl_3$) δ 1.96-2.01 (m, 2H), 2.18-2.22 (m, 2H), 2.44 (s, 3H), 2.99-3.01 (m, 2H), 4.06-4.08 (m, 2H), 7.17-7.20 (m, 2H), 7.23-7.26 (m, 1H), 7.17-7.20 (m, 2H), 7.35-7.37 (m, 1H).

^{13}C NMR (125 MHz, $CDCl_3$) δ 19.8, 26.2, 28.0, 31.4, 52.7, 125.5, 128.9, 129.0, 130.6, 135.5, 140.5, 164.6.

HRMS (ESI): m/z calcd. for $C_{12}H_{16}NS^+$ $[M+H]^+$: 206.0998; found: 206,1000.

(*E*)-2-Styryl-4,5,6,7-tetrahydro-1,3-thiazepine (4i)

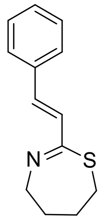

Prepared according to the general procedure, 141.2 mg, 65%. Colorless oil.

^{1}H NMR (600 MHz, $CDCl_3$) δ 1.79-1.83 (m, 2H), 2.02-2.06 (m, 2H), 2.79-2.81 (m, 2H), 4.01-4.03 (m, 2H), 6.88 (d, *J*=16 Hz, 1H), 7.30-7.32 (m, 1H), 7.35-7.37 (m, 2H), 7.50 (d, *J*=7.5 Hz, 2H), 7.54 (d, *J*=16 Hz, 1H).

^{13}C NMR (151 MHz, $CDCl_3$) δ 25.6, 28.5, 30.7, 53.7, 127.5, 128.7, 129.0, 130.5, 135.8, 138.8, 163.5.

HRMS (ESI): m/z calcd. for $C_{13}H_{16}NS^+$ $[M+H]^+$: 218.0998; found: 218.0991.

2-Benzyl-4,5,6,7-tetrahydro-1,3-thiazepine (4j)

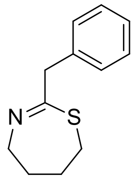

Prepared according to the general procedure, 154 mg, 75%. Colorless oil.

^{1}H NMR (600 MHz, $CDCl_3$) δ 1.79-1.83 (m, 2H), 1.91-1.95 (m, 2H), 2.68-2.70 (m, 2H), 3.72 (s, 2H), 3.85-3.87 (m, 2H), 7.24-7.33 (5H, m).

^{13}C NMR (151 MHz, $CDCl_3$) δ 25.8, 28.0, 30.5, 49.7, 52.4, 126.7, 128.4, 129.1, 136.7, 164.8. **HRMS (ESI):** m/z calcd. for $C_{12}H_{16}NS^+$ $[M+H]^+$: 206.0998; found: 206.1003.

2-Pentyl-4,5,6,7-tetrahydro-1,3-thiazepine (4k)

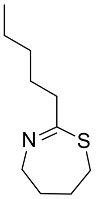

Prepared according to the general procedure, 148.3 mg, 80%. Colorless oil.

^{1}H NMR (600 MHz, $CDCl_3$) δ 0.88-0.90 (m, 3H), 1.28-1.35 (m, 4H), 1.60-1.67 (m, 2H), 1.78-1.83 (m, 2H), 1.98-2.02 (m, 2H), 2.38 (t, *J*=7.7 Hz, 2H), 2.78-2.79 (m, 2H), 3.79-3.81 (m, 2H).

^{13}C NMR (151 MHz, $CDCl_3$) δ 14.0, 22.4, 26.0, 27.3, 28.0, 30.3, 31.3, 43.4, 52.2, 166.5.

HRMS (ESI): m/z calcd. for $C_{10}H_{20}NS^+$ $[M+H]^+$: 186.1311; found: 186.1307.

2-Isopropyl-4,5,6,7-tetrahydro-1,3-thiazepine (4l)

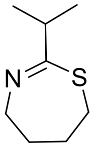

Prepared according to the general procedure, 149.4 mg, 95%. Colorless oil.

^{1}H NMR (600 MHz, $CDCl_3$) δ 1.16 (d, *J*=6.9 Hz, 6H), 1.76-1.80 (m, 2H), 1.95-1.99 (m, 2H), 2.60 (h, *J*=6.9 Hz, 1H), 2.74-2.76 (m, 2H), 3.81-3.83 (m, 2H).

^{13}C NMR (151 MHz, $CDCl_3$) δ 20.5, 25.7, 28.0, 30.2, 41.5, 52.3, 171.6.

HRMS (ESI): m/z calcd. for $C_8H_{16}NS^+$ $[M+H]^+$: 158.0998; found: 158.1051.

2-(*tert*-Butyl)-4,5,6,7-tetrahydro-1,3-thiazepine (4m)

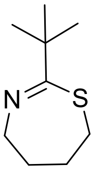

Prepared according to the general procedure, 68.5 mg, 40%. Colorless oil.

^{1}H NMR (600 MHz, $CDCl_3$) δ 1.20 (s, 9H), 1.71-1.75 (m, 2H), 1.91-1.95 (m, 2H), 2.67-2.69 (m, 2H), 3.83-3.85 (m, 2H).

^{13}C NMR (151 MHz, $CDCl_3$) δ 25.3, 28.0, 28.2, 30.3, 43.1, 52.8, 173.9.

HRMS (ESI): m/z calcd. for $C_9H_{18}NS^+$ $[M+H]^+$: 172.1154; found: 172.1147.

4. Copies of ^{1}H and ^{13}C NMR spectra of compounds 1–4

^{1}H NMR (600 MHz, $CDCl_3$) spectrum of compound **1a**

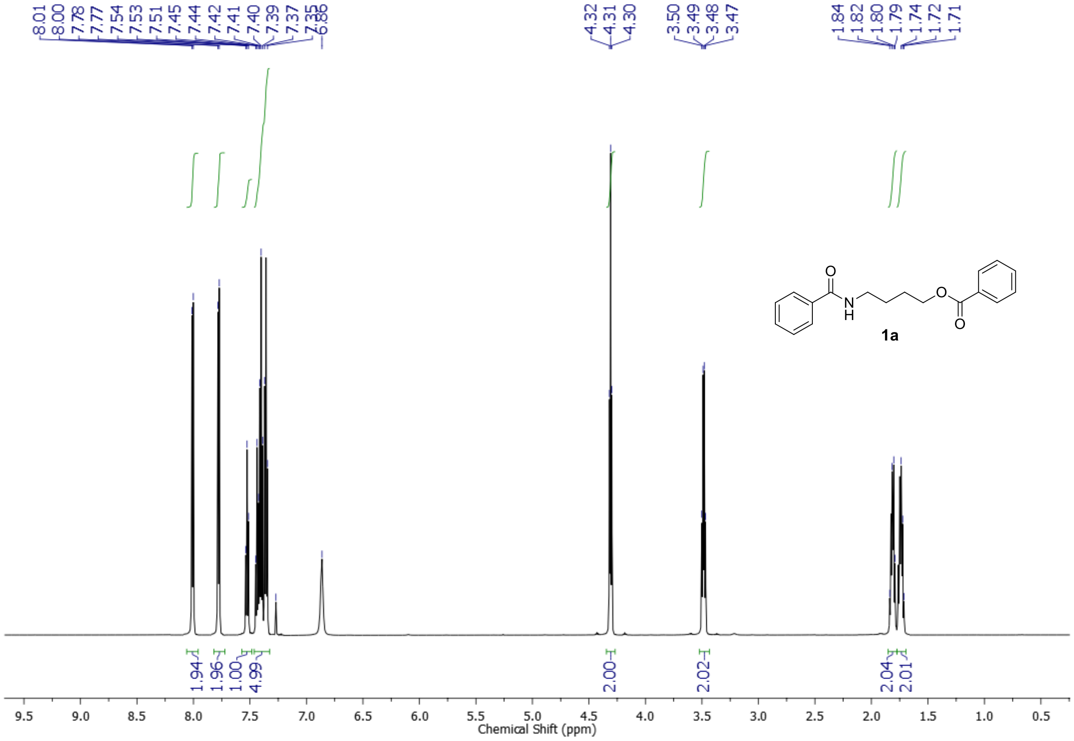

^{13}C NMR (151 MHz, $CDCl_3$) spectrum of compound **1a**

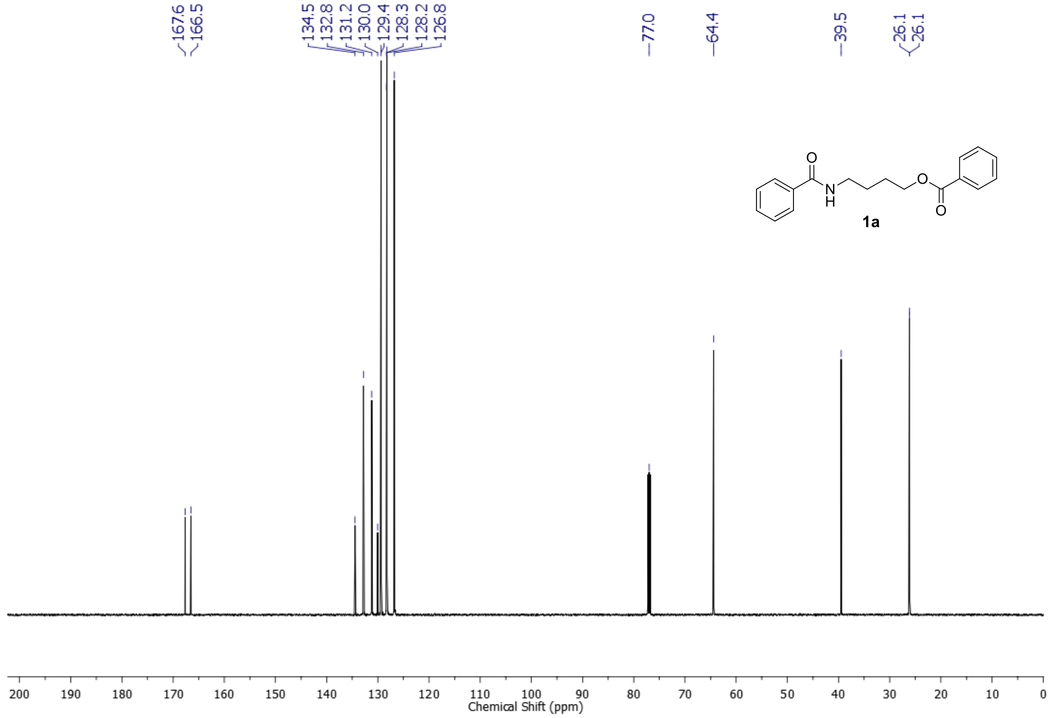

^{1}H NMR (500 MHz, $CDCl_3$) spectrum of compound **1b**

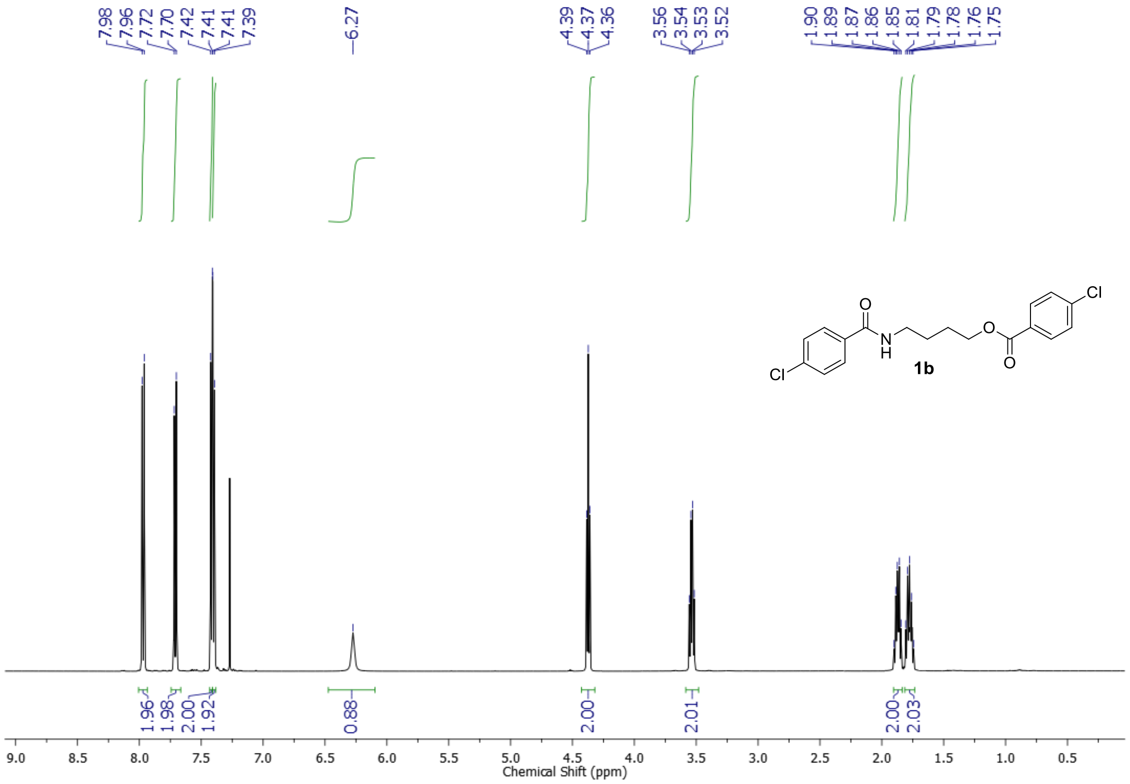

^{13}C NMR (125 MHz, $CDCl_3$) spectrum of compound **1b**

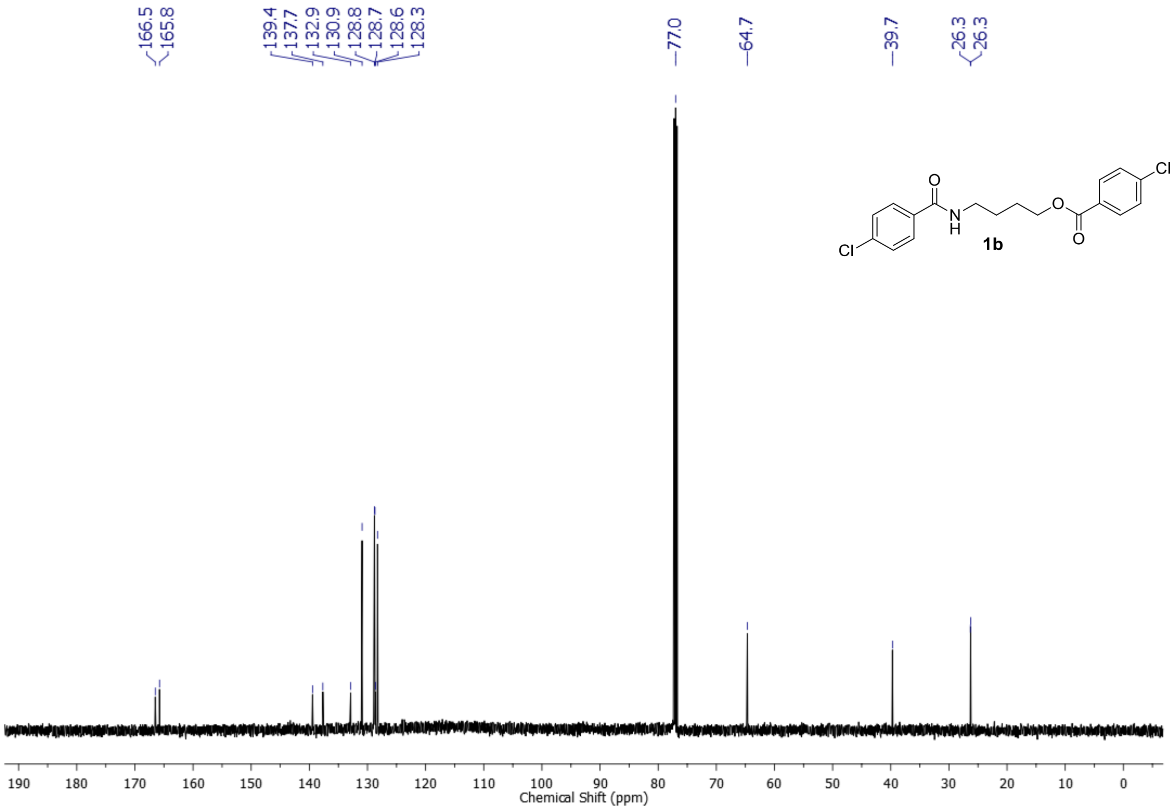

^{1}H NMR (500 MHz, $CDCl_3$) spectrum of compound **1c**

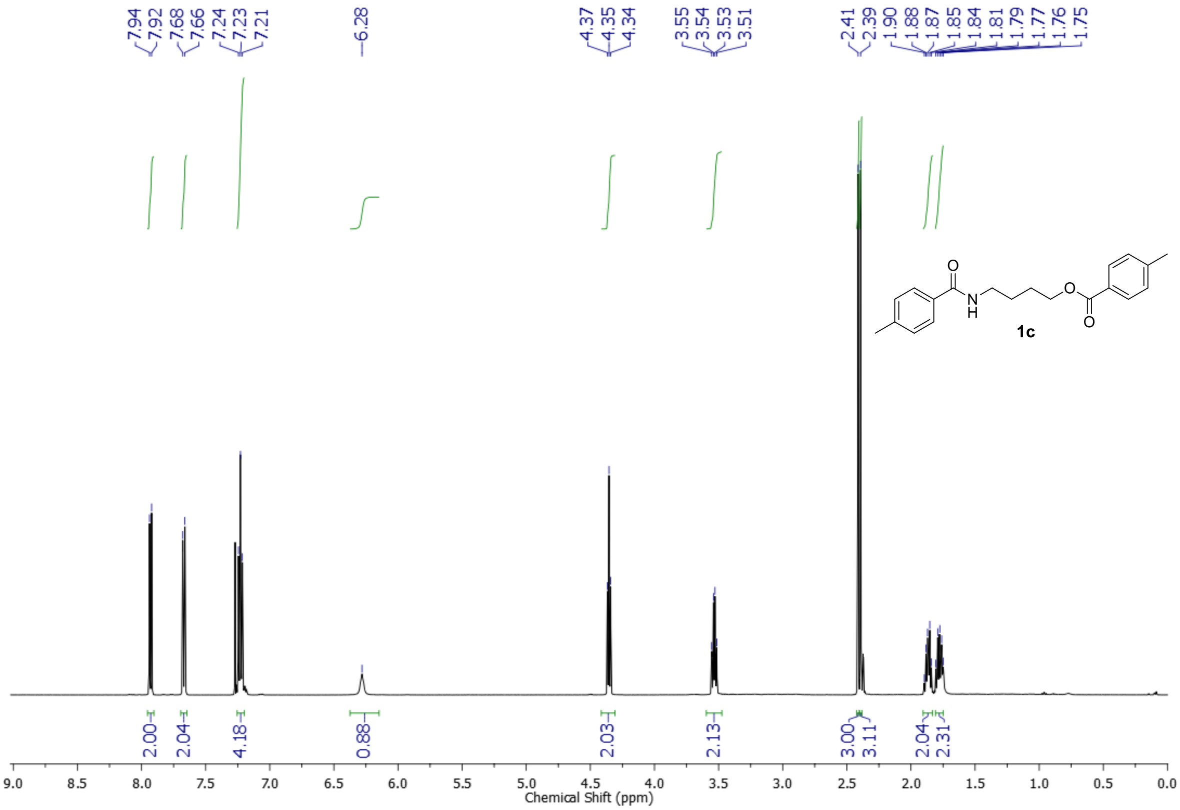

^{13}C NMR (125 MHz, $CDCl_3$) spectrum of compound **1c**

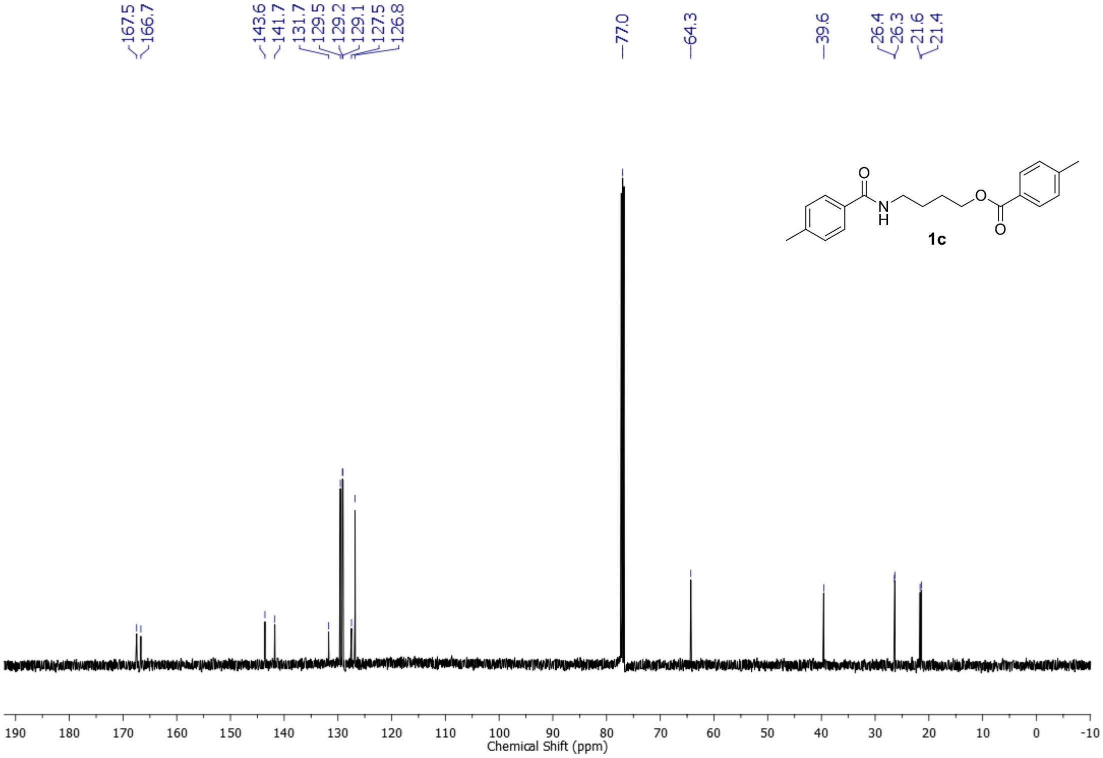

^{1}H NMR (500 MHz, $CDCl_3$) spectrum of compound **1d**

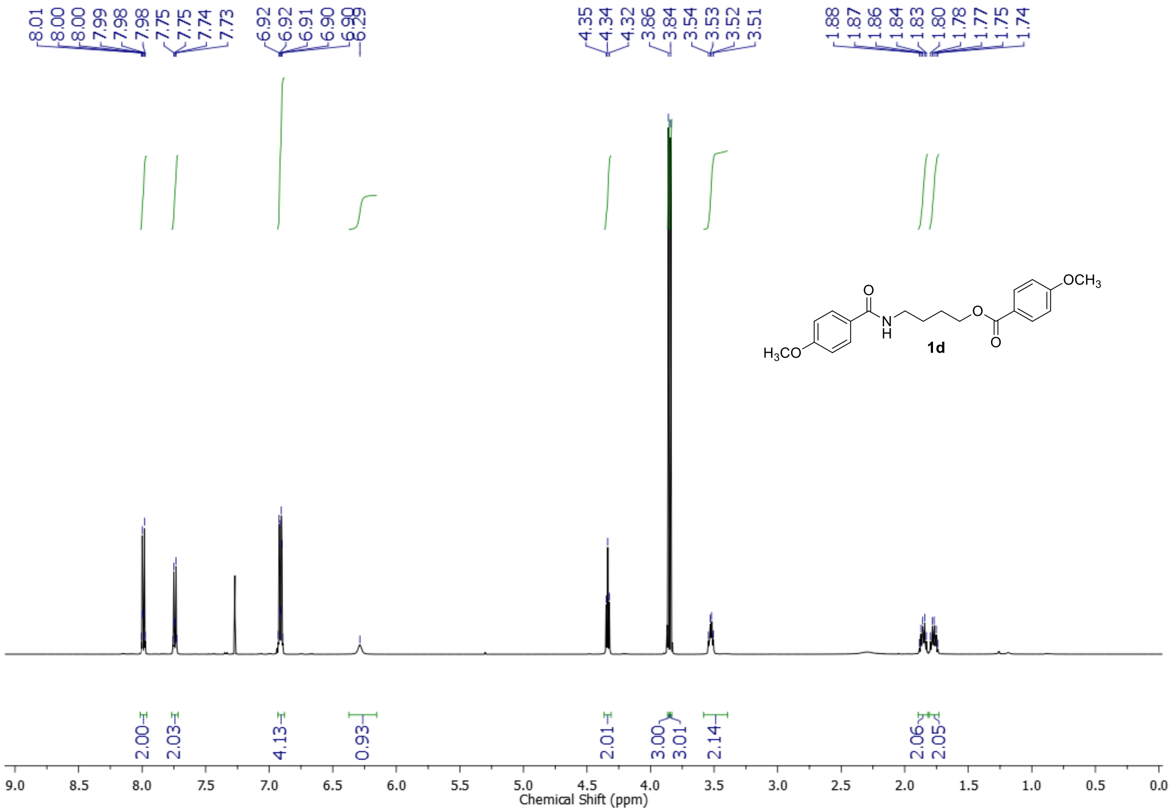

^{13}C NMR (125 MHz, $CDCl_3$) spectrum of compound **1d**

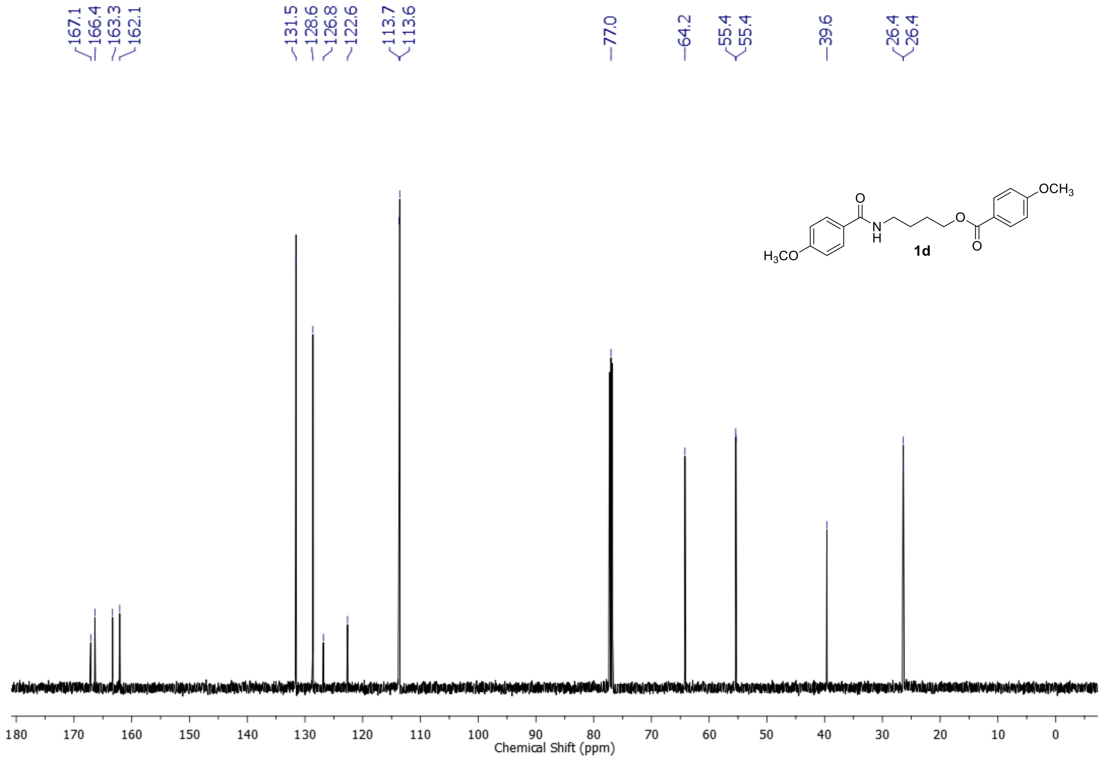

^{1}H NMR (500 MHz, $CDCl_3$) spectrum of compound **1e**

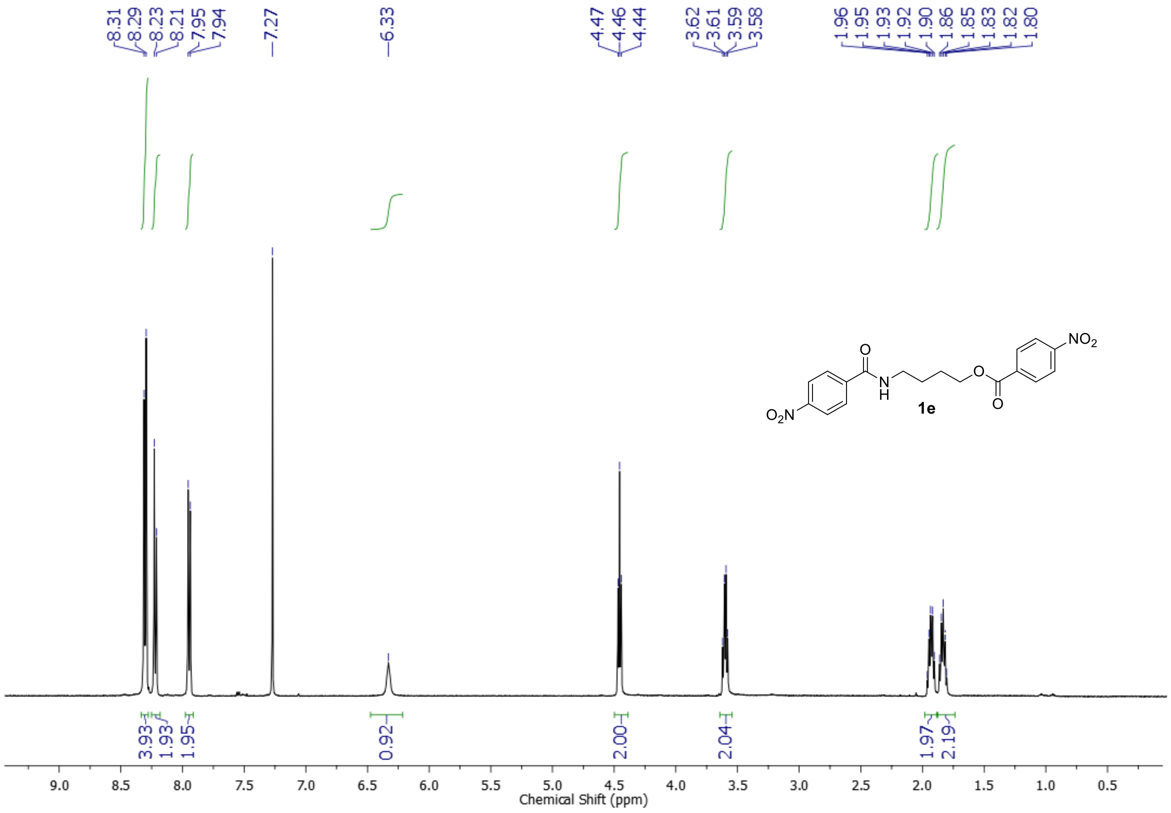

^{13}C NMR (125 MHz, $CDCl_3$) spectrum of compound **1e**

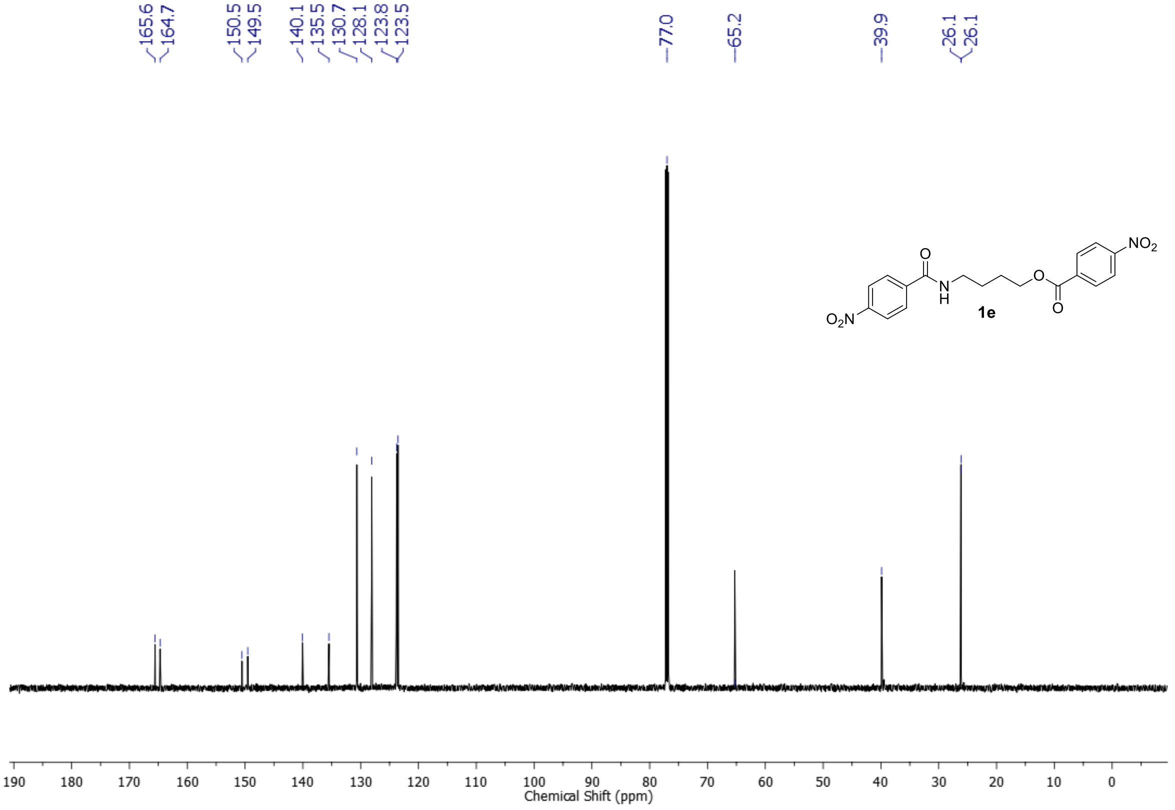

^{1}H NMR (500 MHz, $CDCl_3$) spectrum of compound **1f**

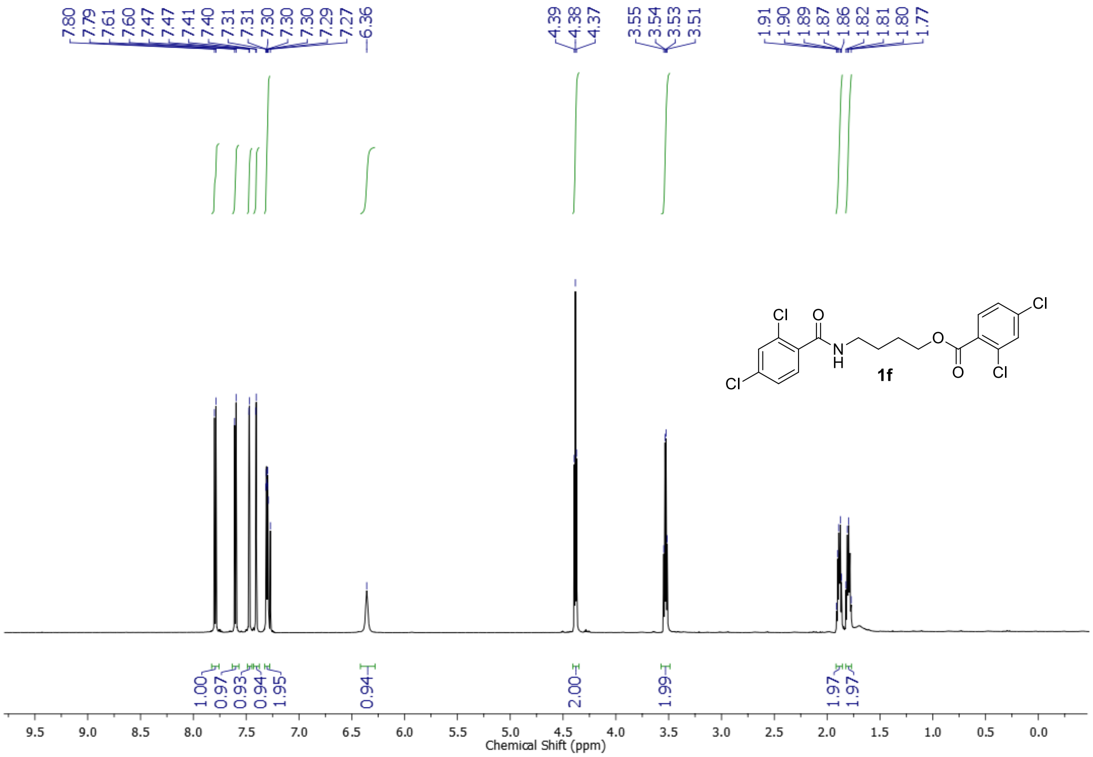

^{13}C NMR (125 MHz, $CDCl_3$) spectrum of compound **1f**

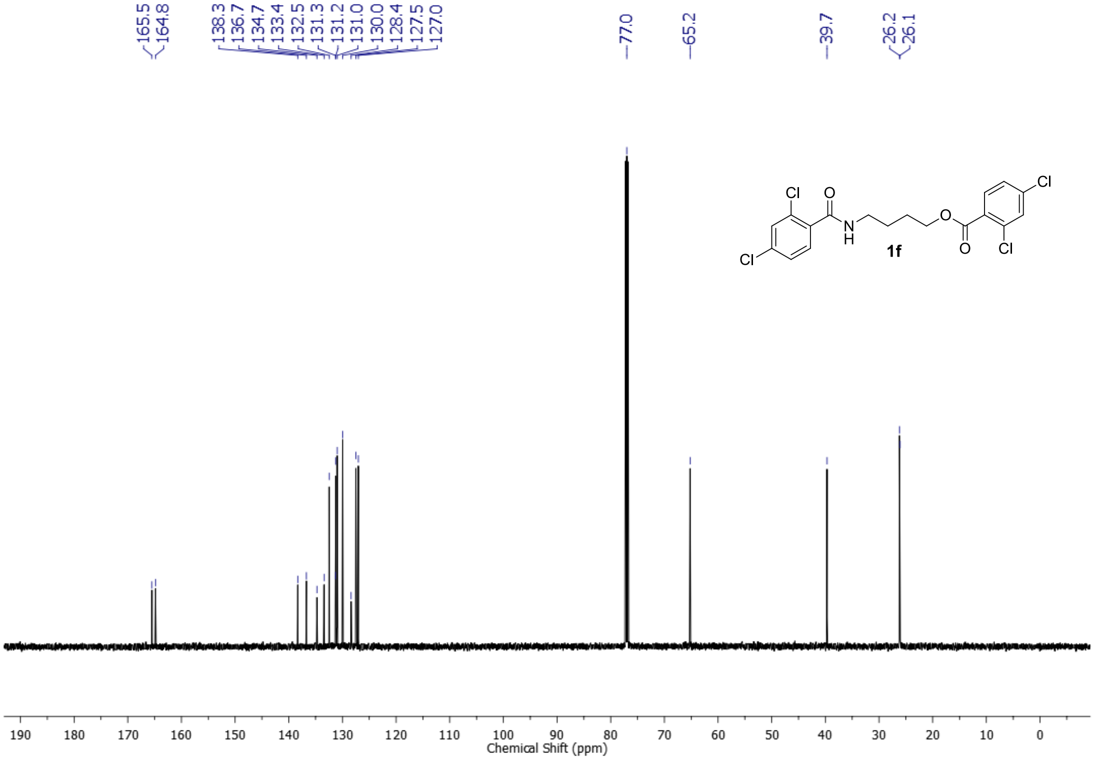

^{1}H NMR (500 MHz, $CDCl_3$) spectrum of compound **1g**

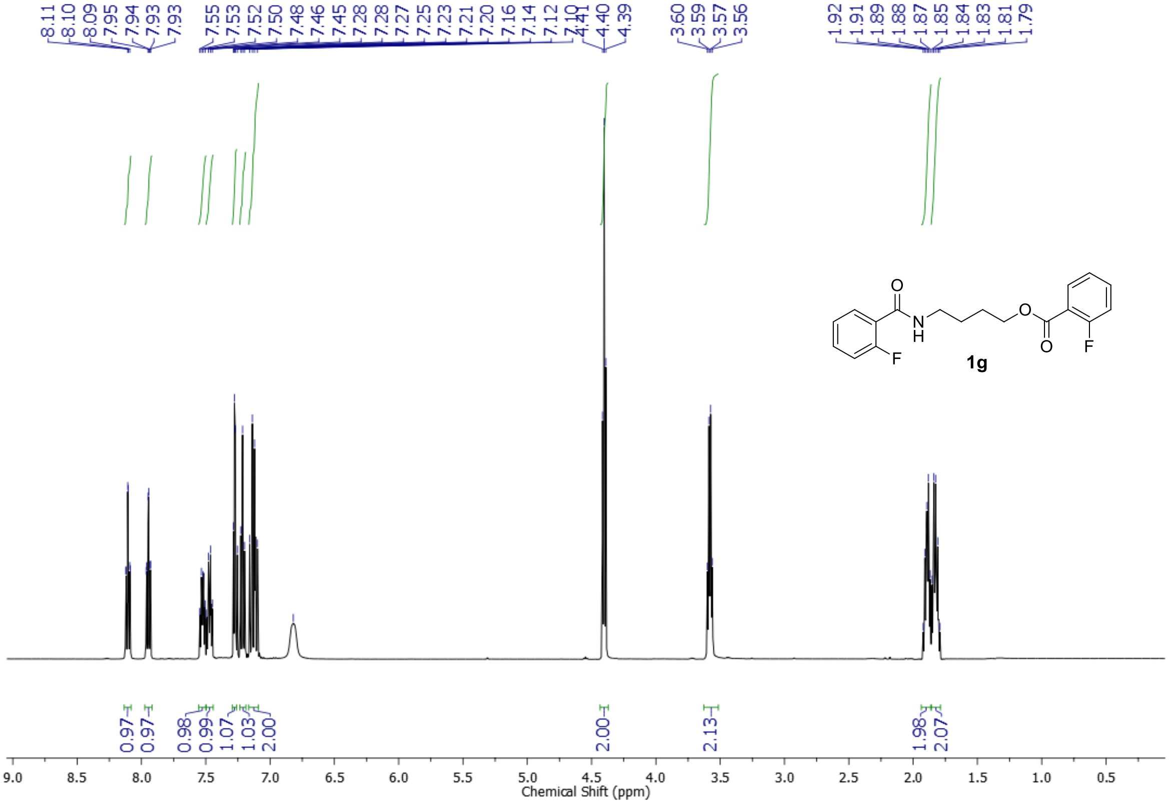

^{13}C NMR (125 MHz, $CDCl_3$) spectrum of compound **1g**

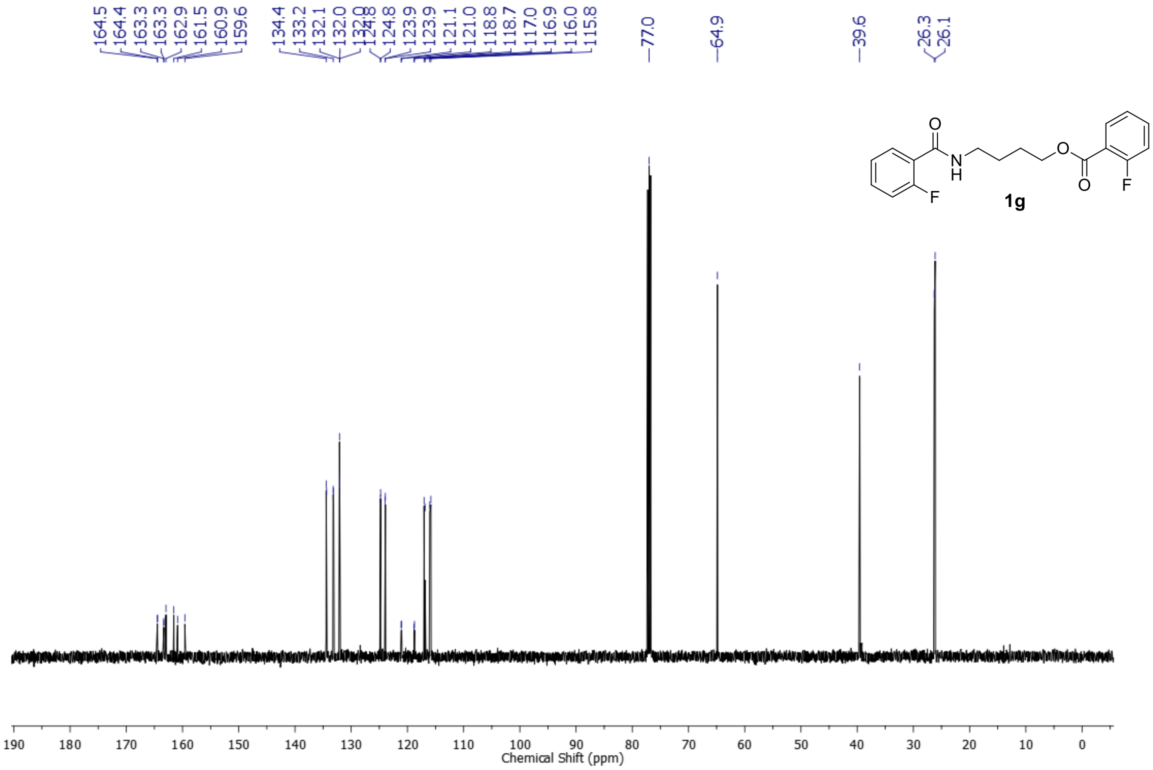

^{1}H NMR (600 MHz, $CDCl_3$) spectrum of compound **1h**

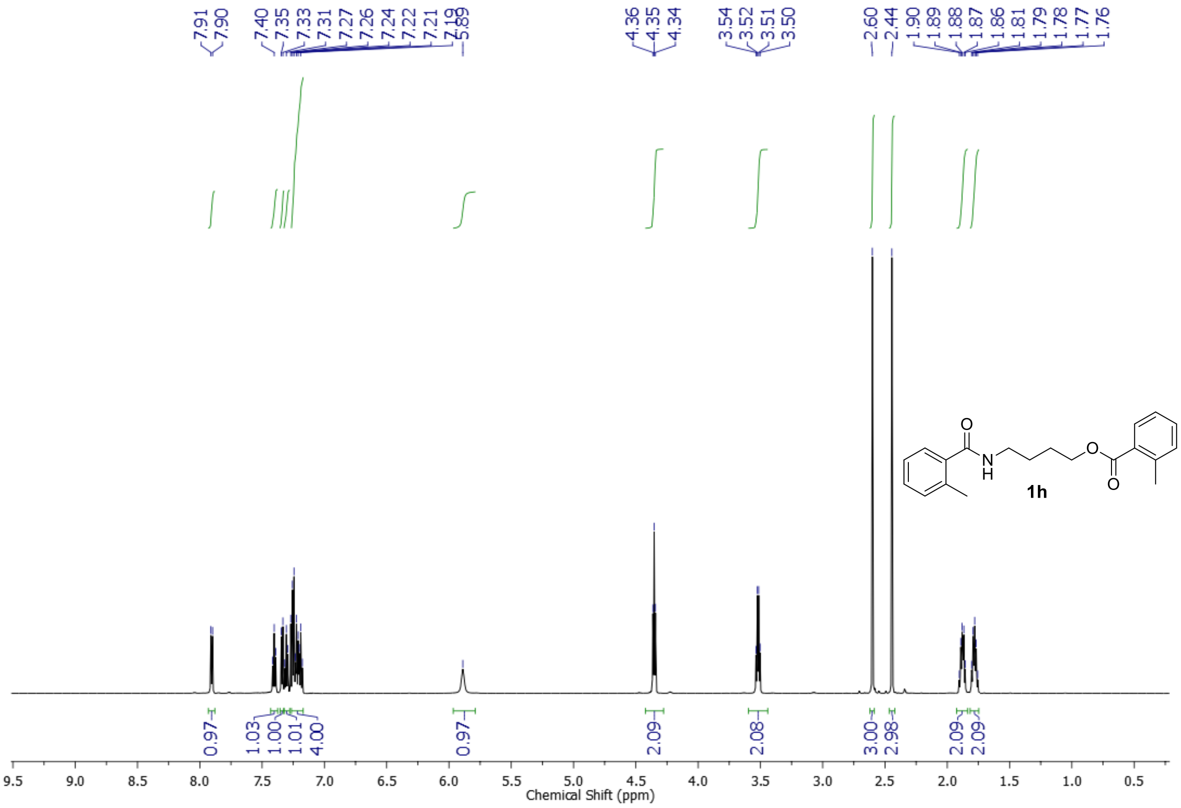

^{13}C NMR (151 MHz, $CDCl_3$) spectrum of compound **1h**

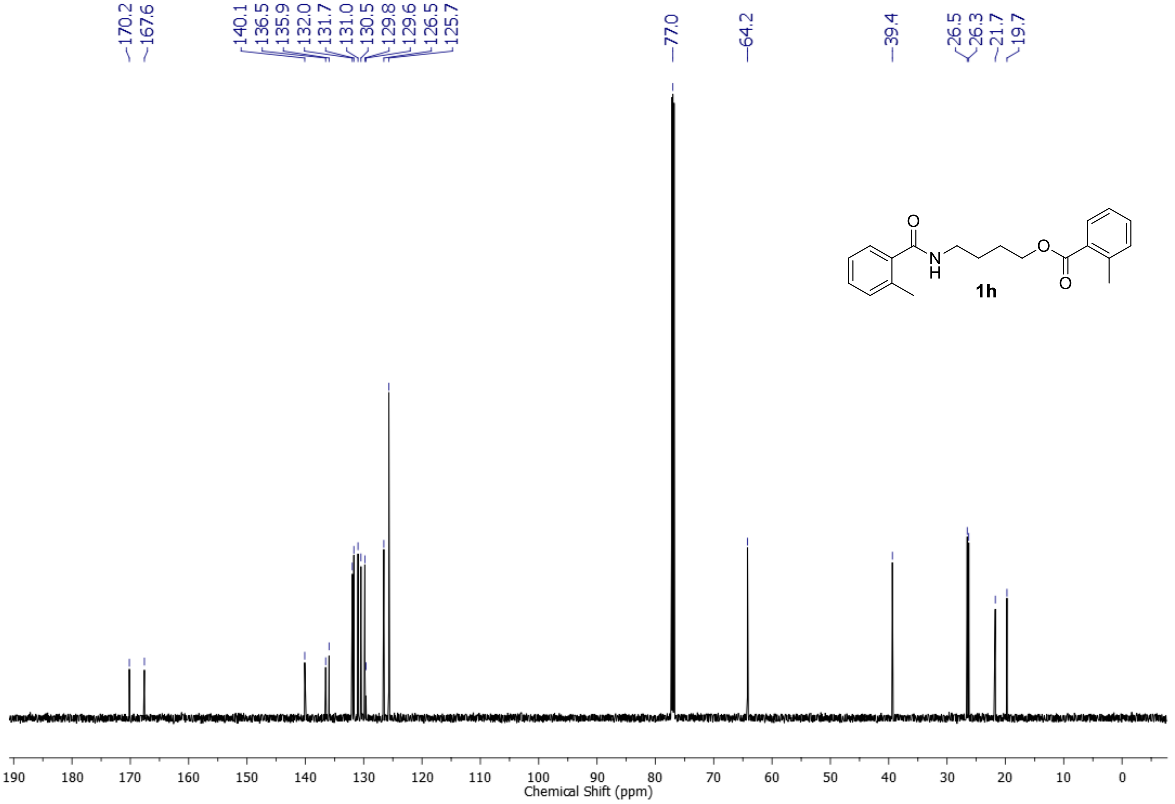

^{1}H NMR (600 MHz, $CDCl_3$) spectrum of compound **1i**

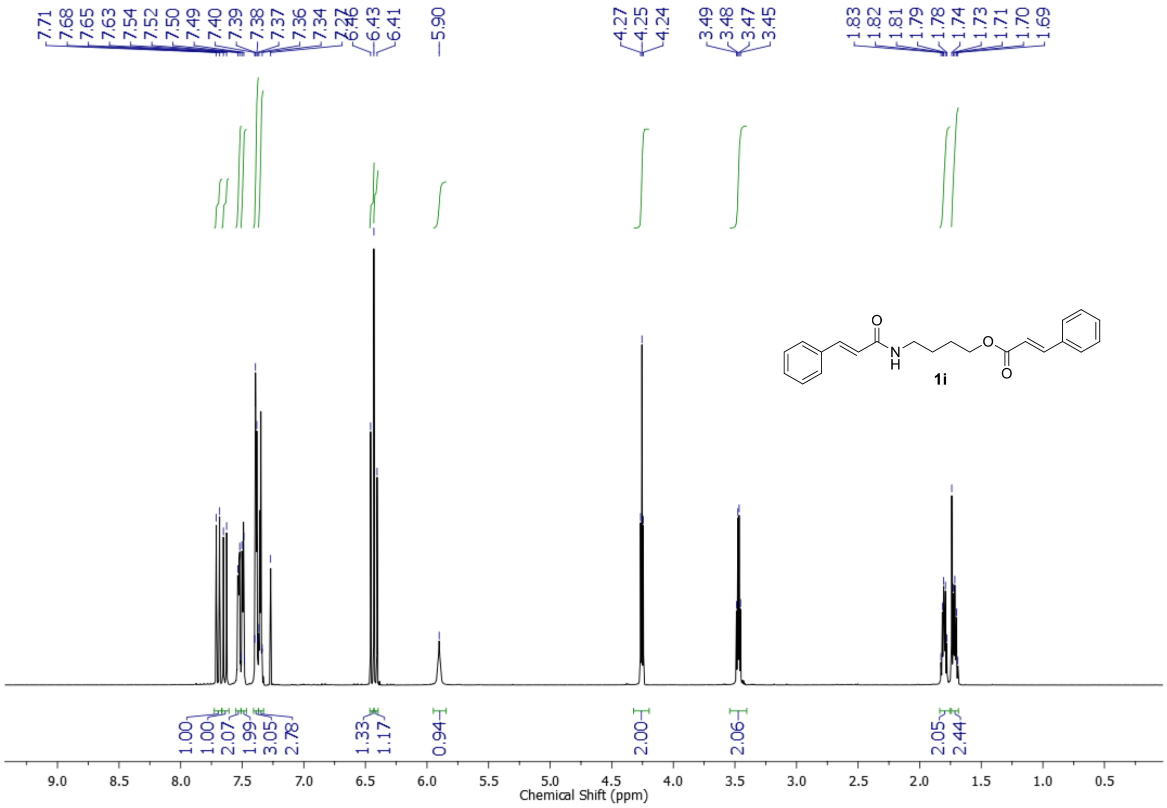

^{13}C NMR (151 MHz, $CDCl_3$) spectrum of compound **1i**

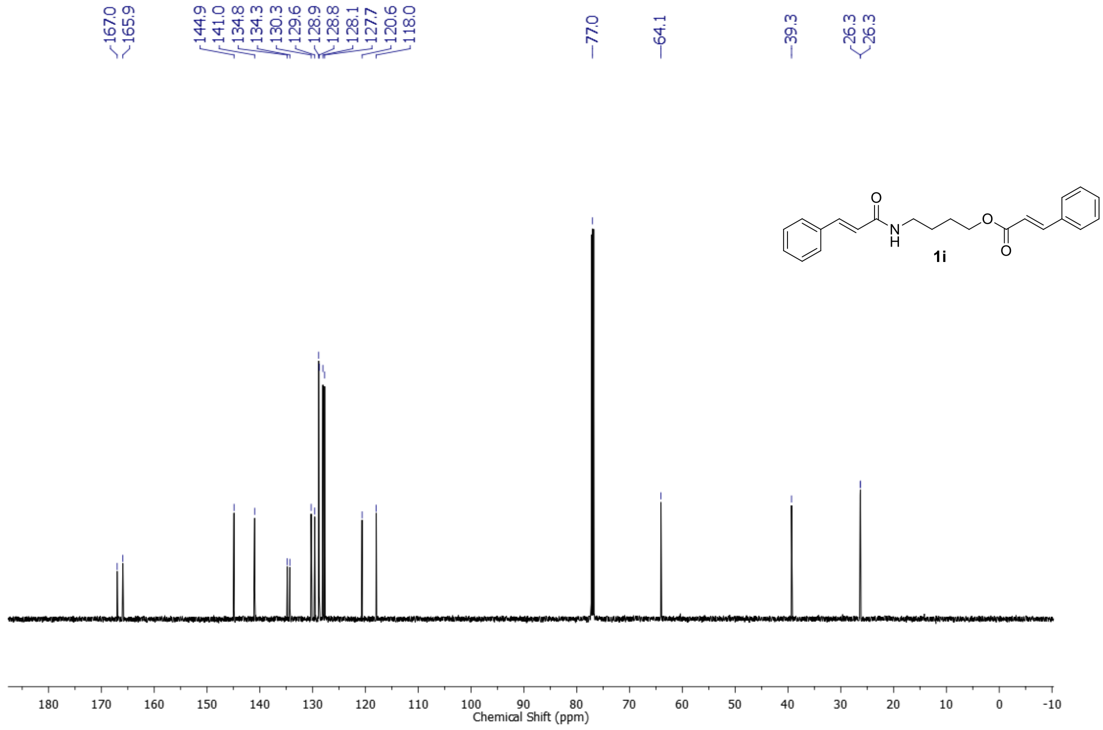

^{1}H NMR (600 MHz, $CDCl_3$) spectrum of compound **1j**

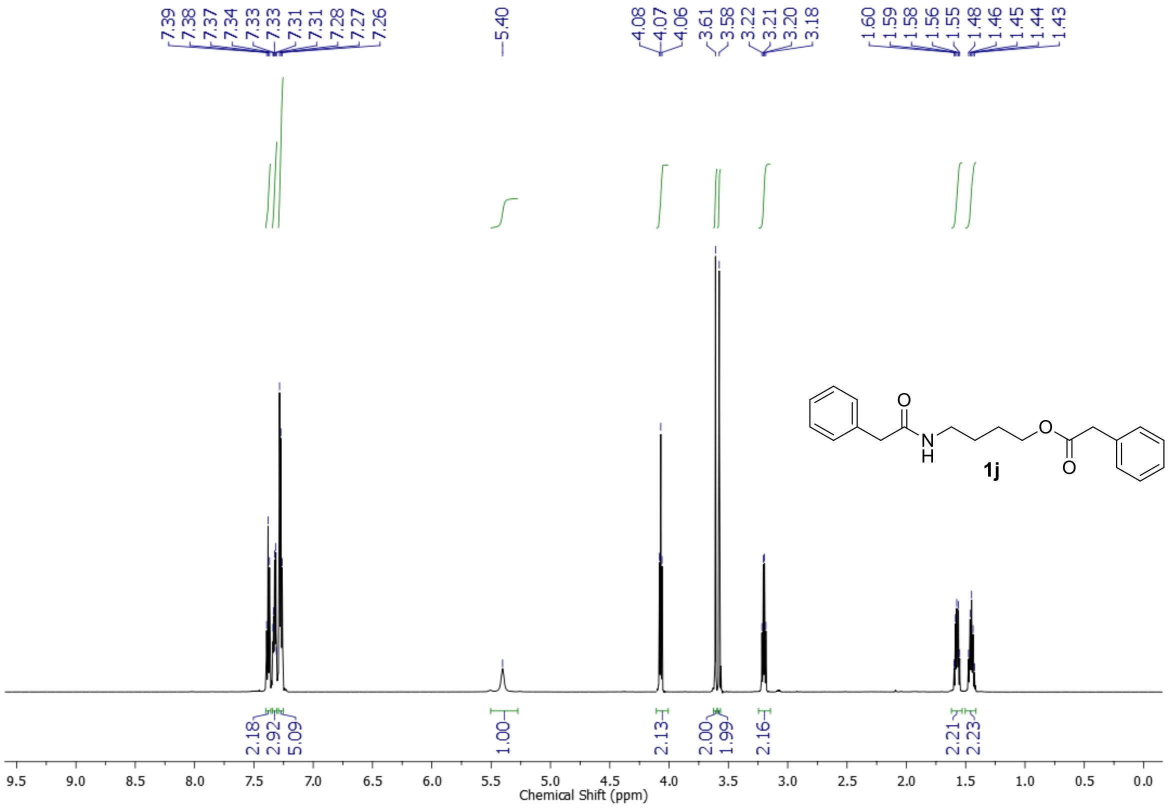

^{13}C NMR (151 MHz, $CDCl_3$) spectrum of compound **1j**

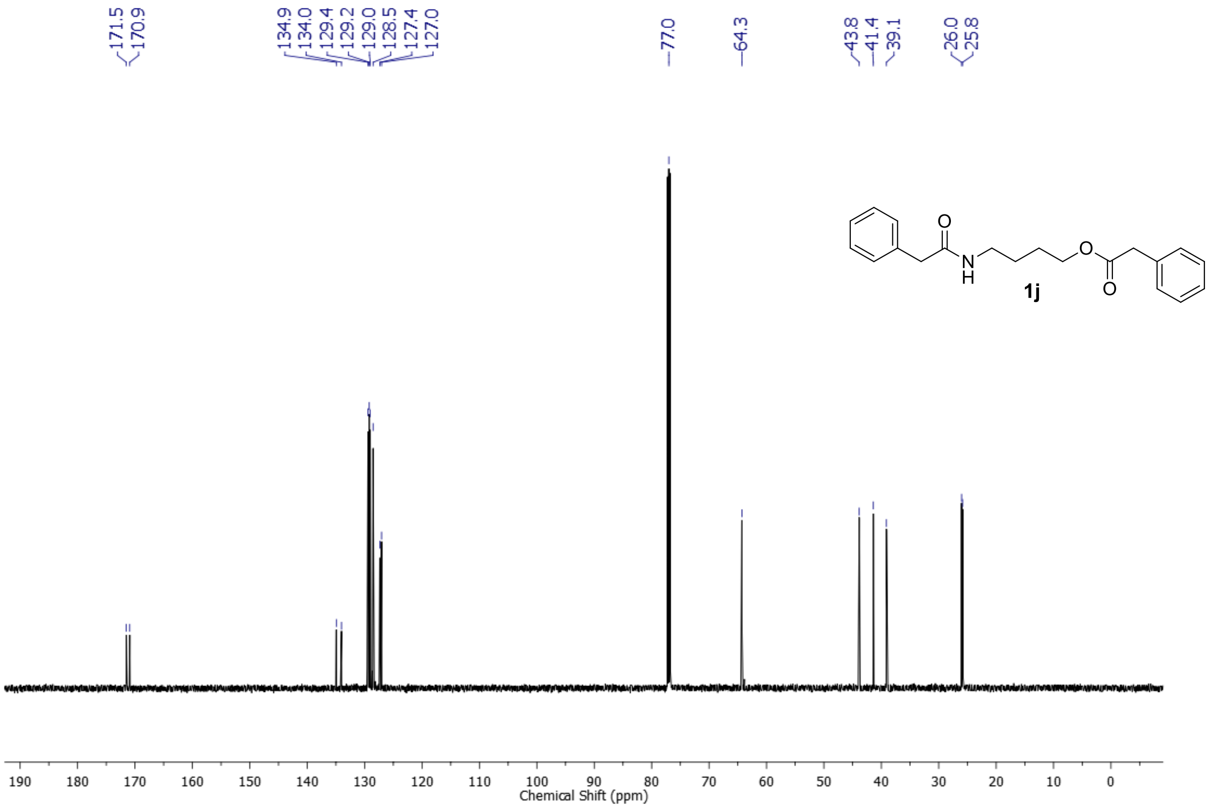

^{1}H NMR (600 MHz, $CDCl_3$) spectrum of compound **1k**

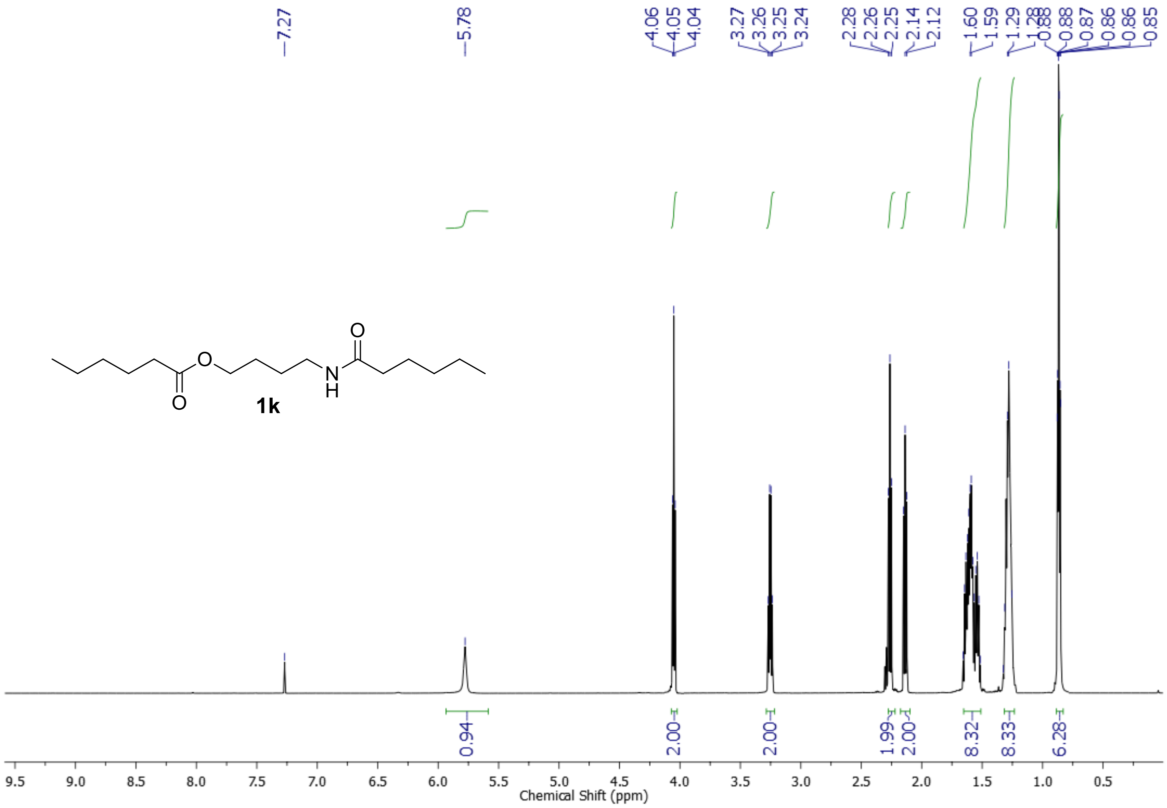

^{13}C NMR (151 MHz, $CDCl_3$) spectrum of compound **1k**

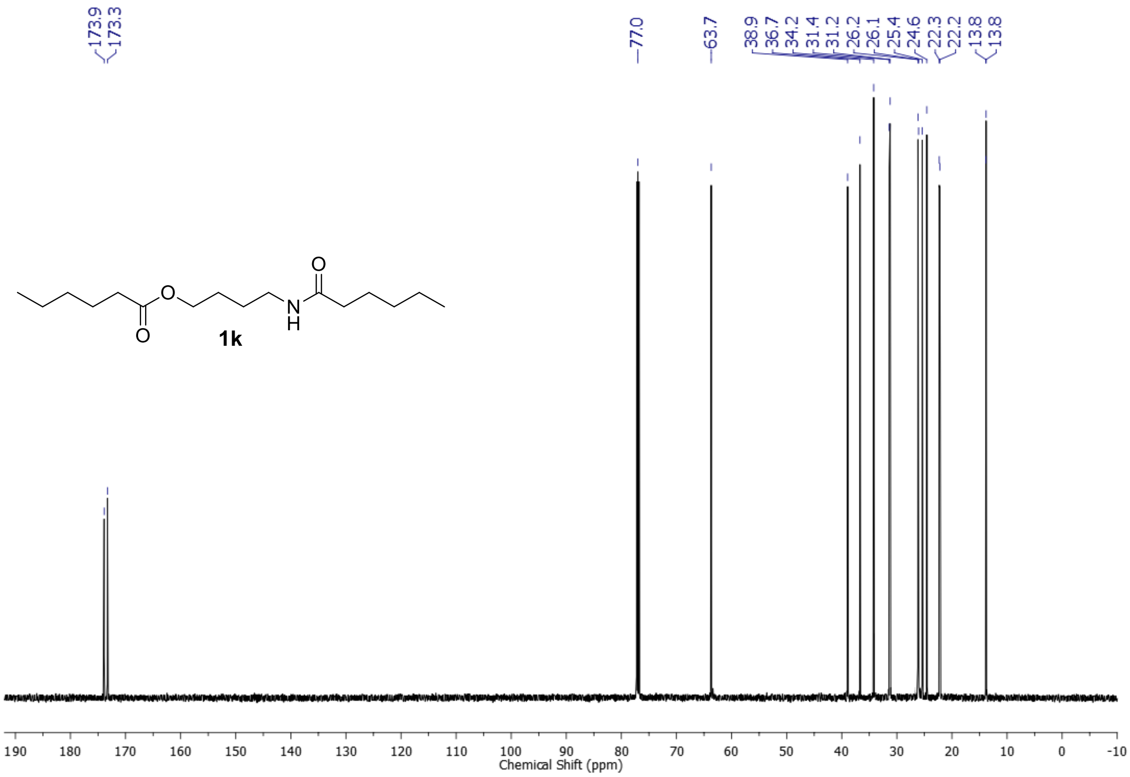

^{1}H NMR (600 MHz, $CDCl_3$) spectrum of compound **1l**

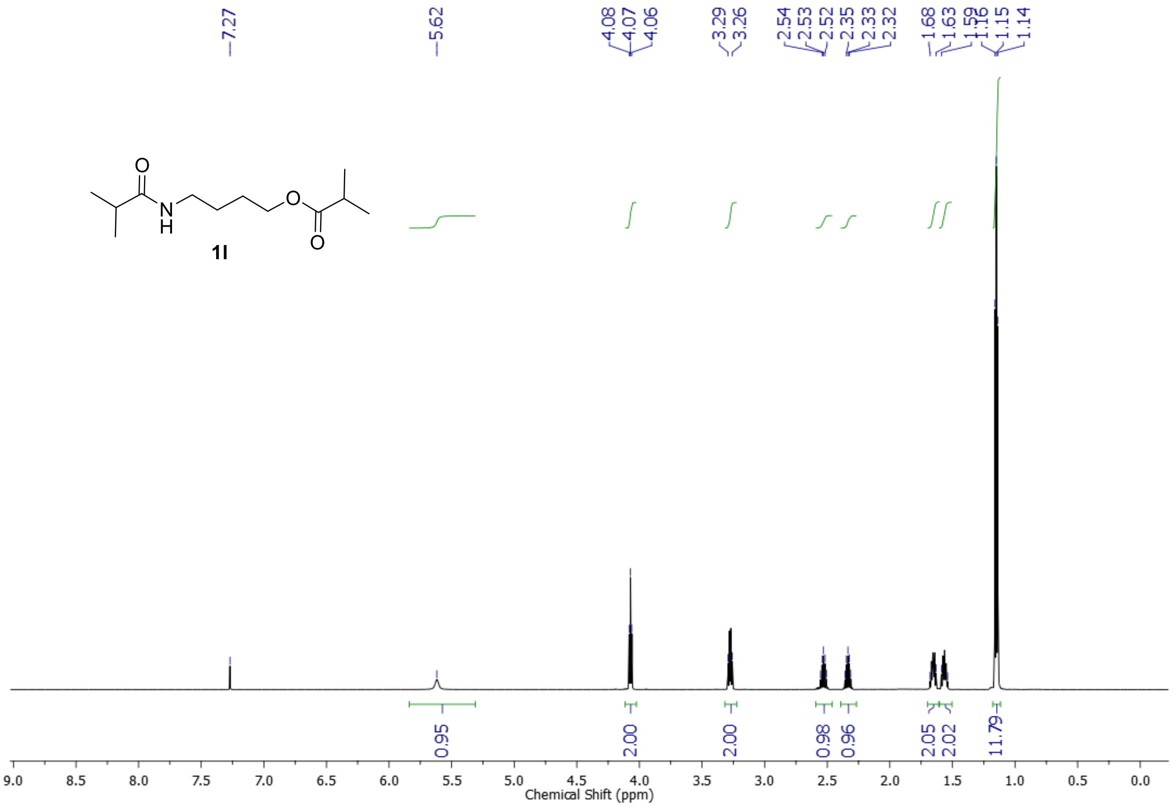

^{13}C NMR (151 MHz, $CDCl_3$) spectrum of compound **1l**

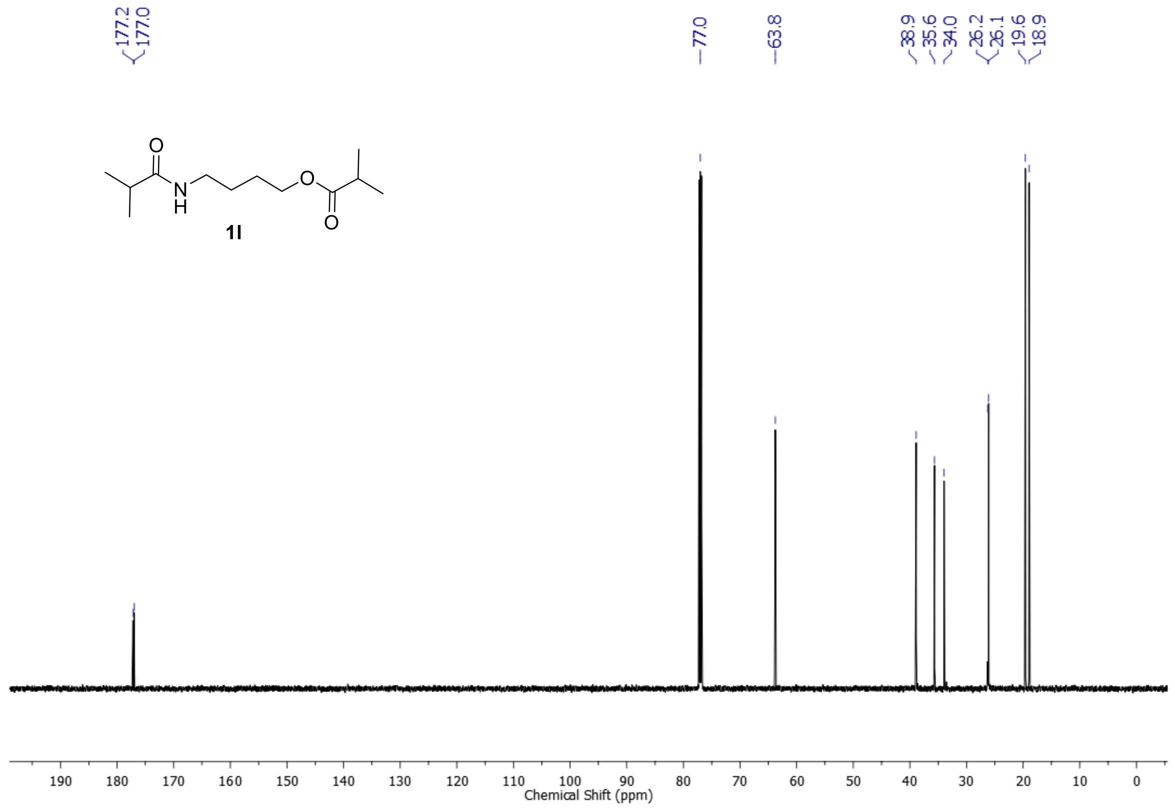

^{1}H NMR (600 MHz, $CDCl_3$) spectrum of compound **1m**

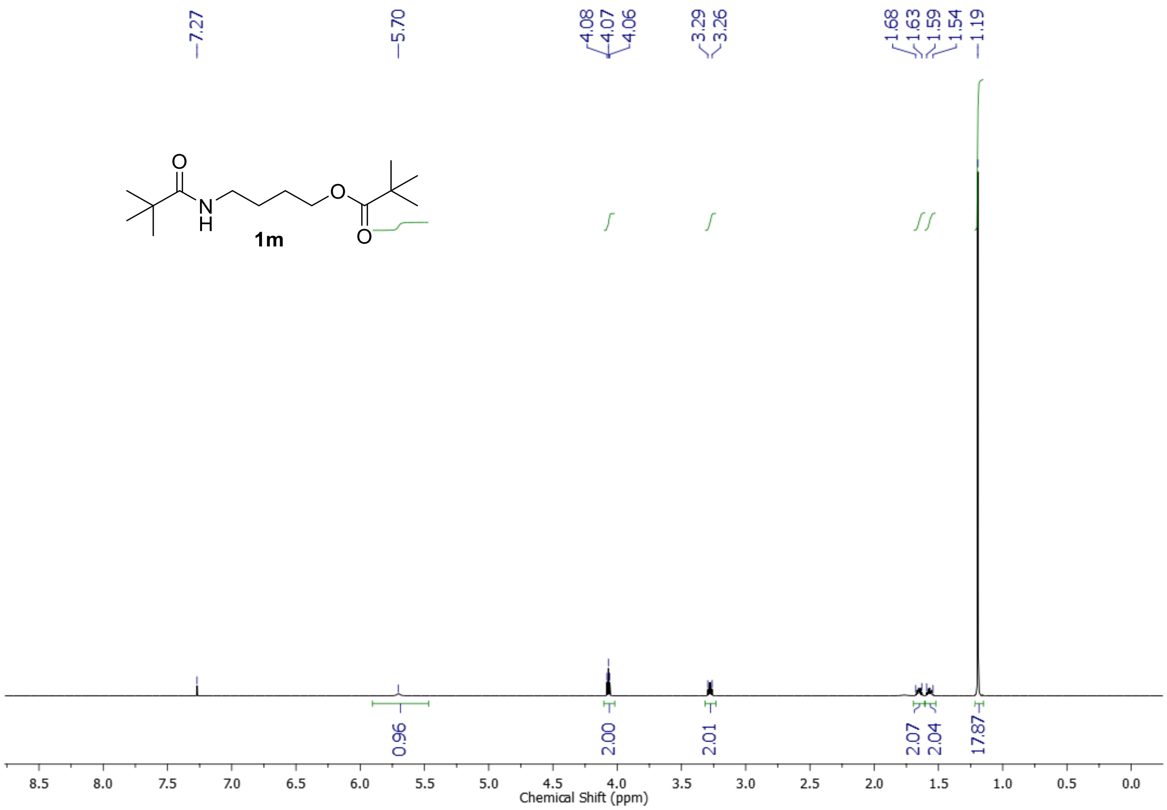

^{13}C NMR (151 MHz, $CDCl_3$) spectrum of compound **1m**

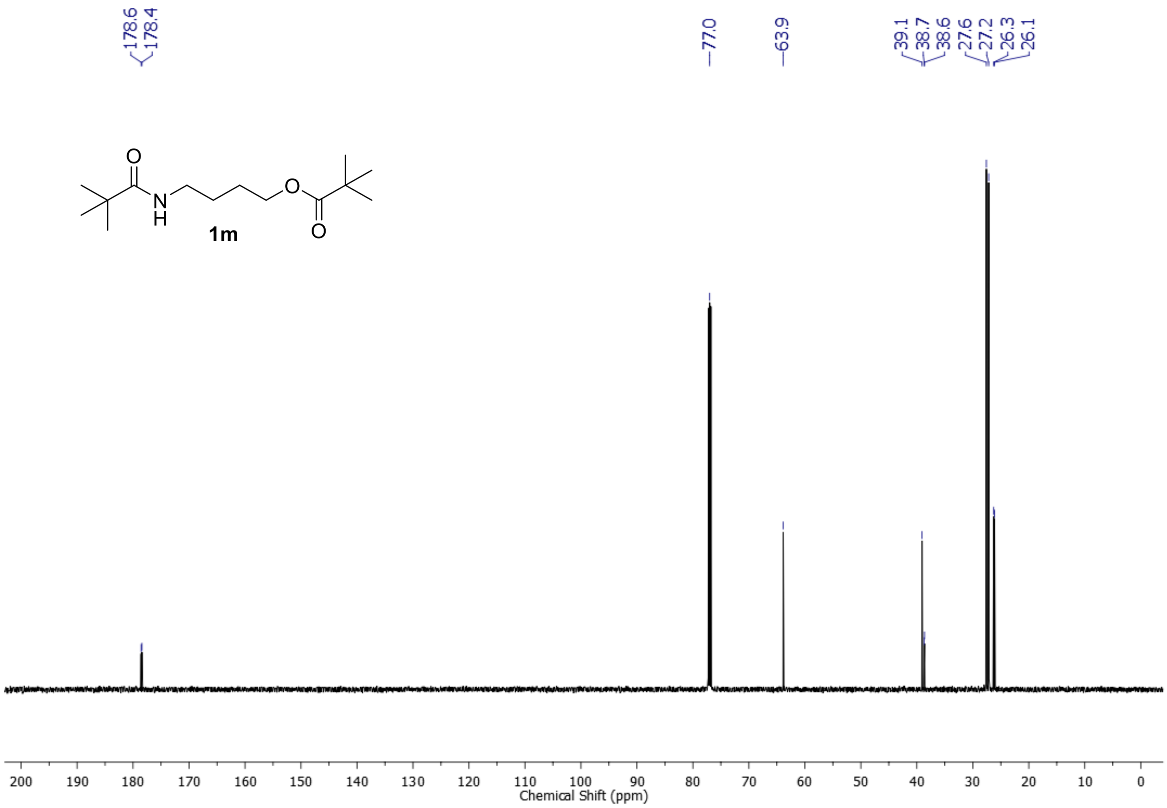

^{1}H NMR (600 MHz, $CDCl_3$) spectrum of compound **2a**

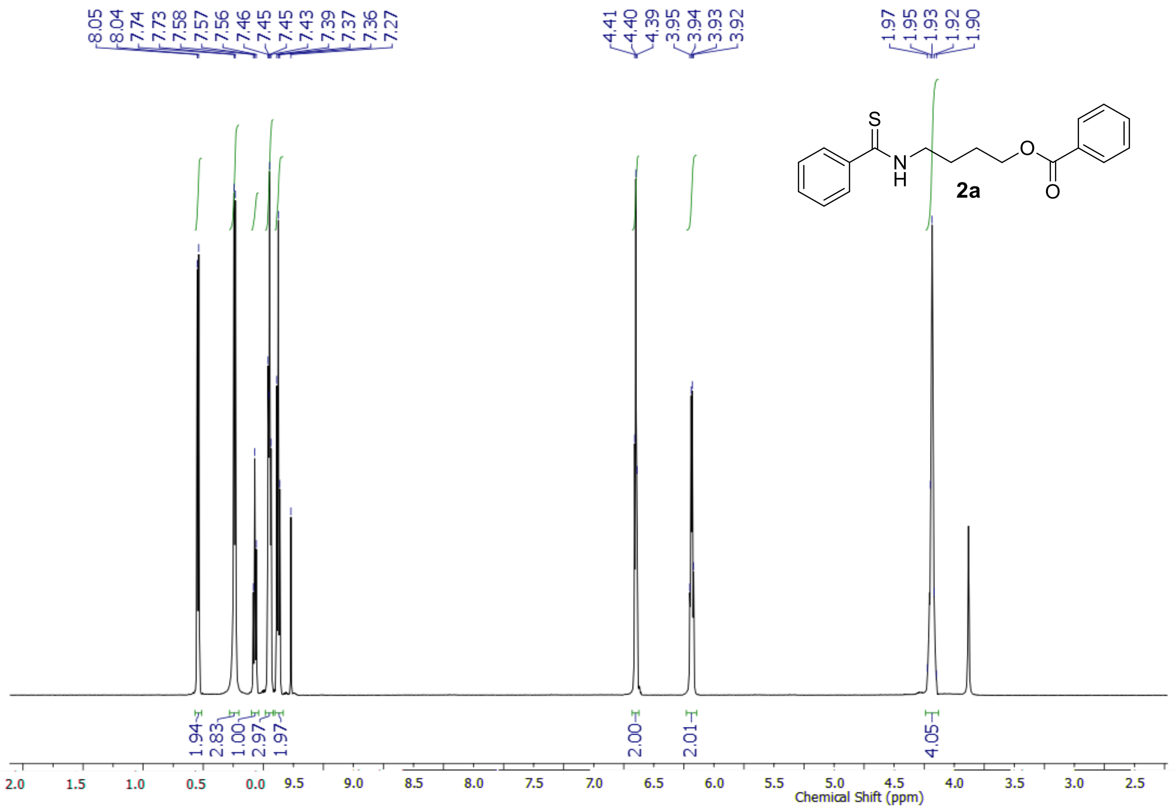

^{13}C NMR (151 MHz, $CDCl_3$) spectrum of compound **2a**

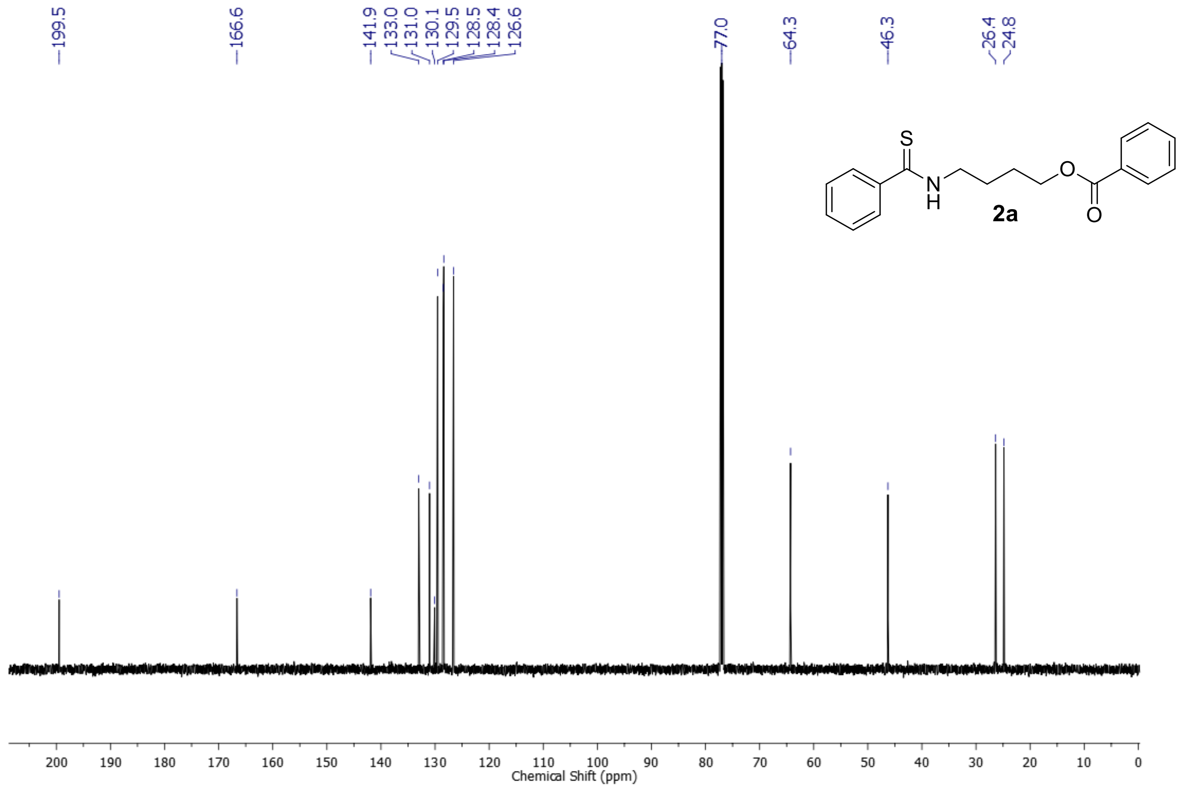

^{1}H NMR (500 MHz, $CDCl_3$) spectrum of compound **2b**

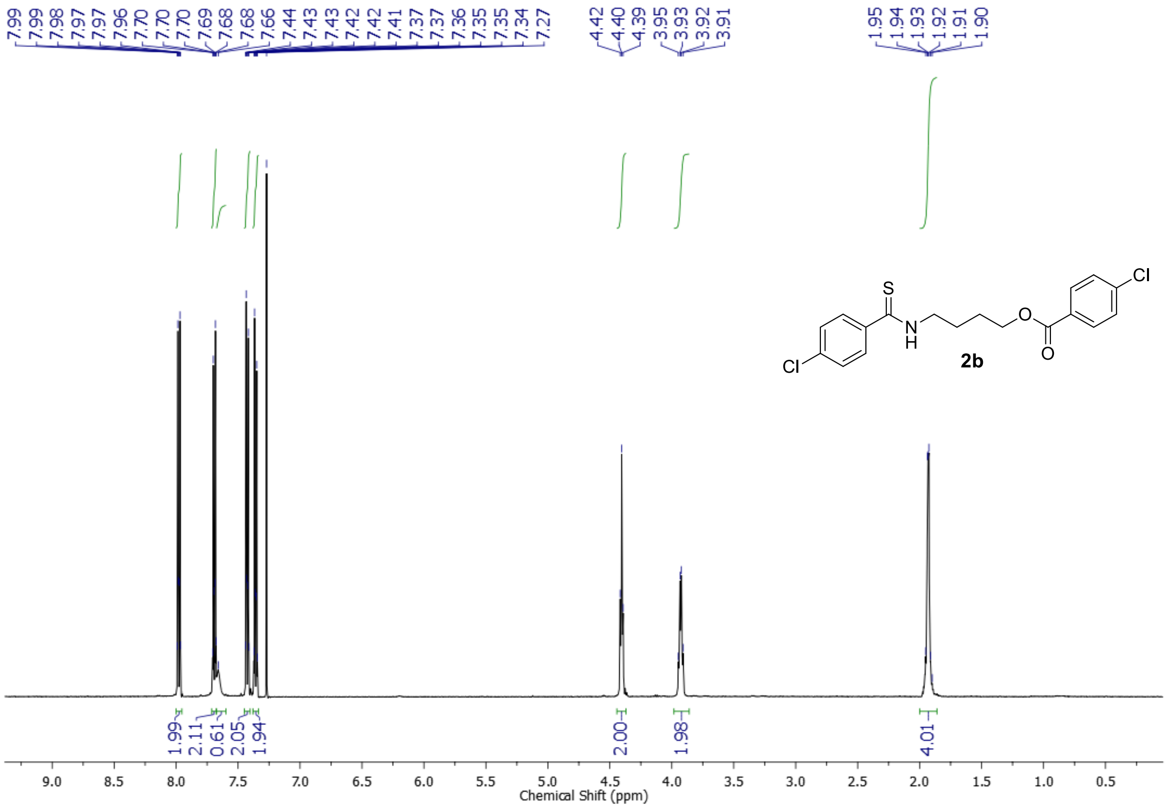

^{13}C NMR (125 MHz, $CDCl_3$) spectrum of compound **2b**

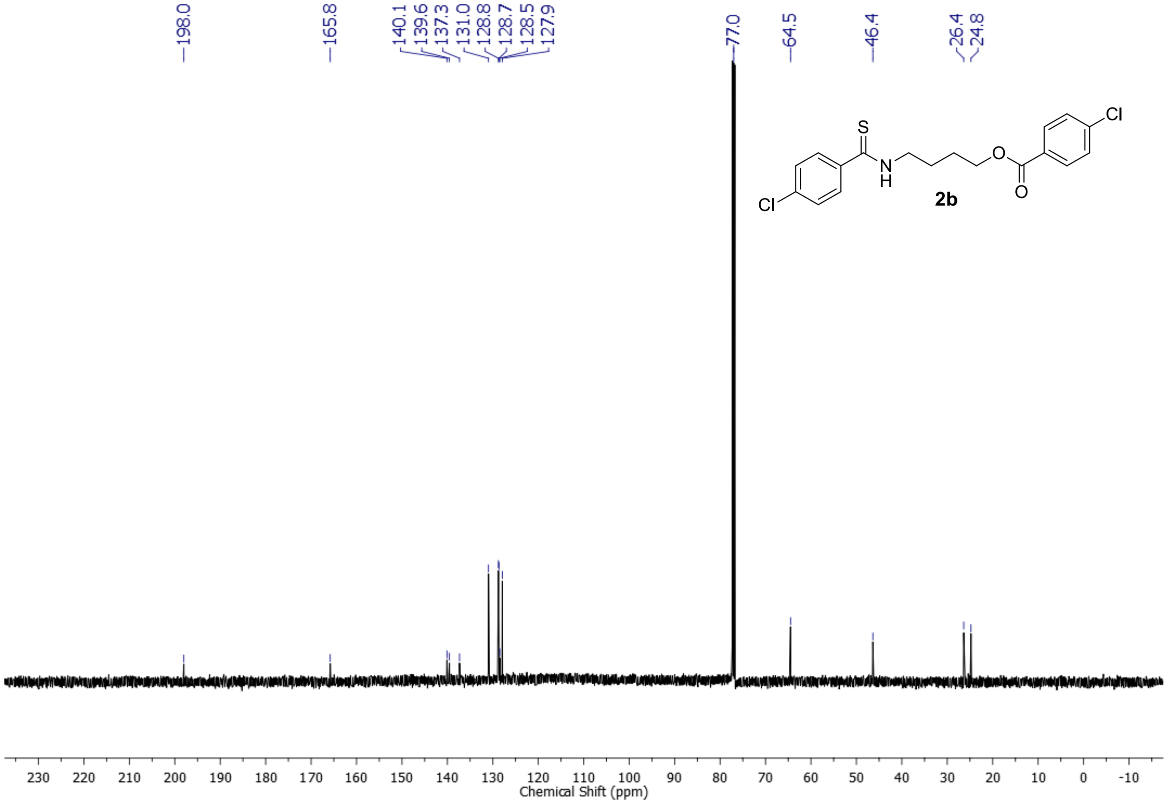

^{1}H NMR (500 MHz, $CDCl_3$) spectrum of compound **2c**

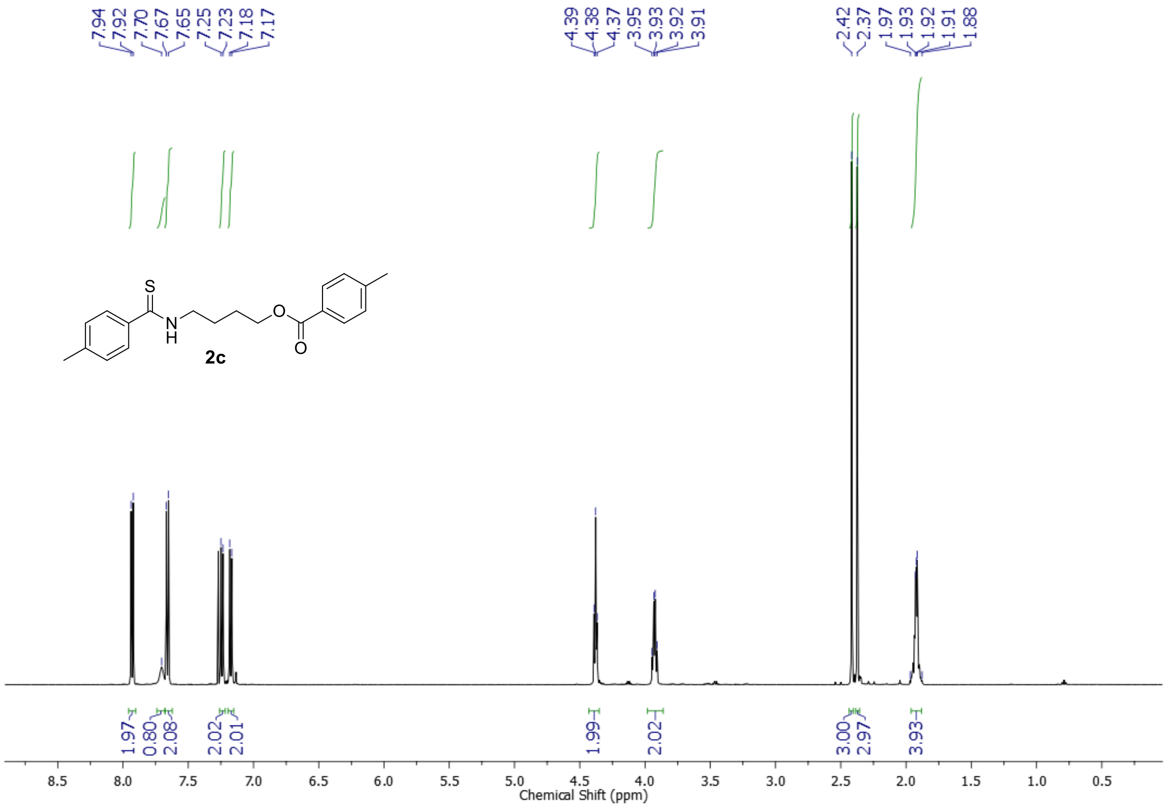

^{13}C NMR (125 MHz, $CDCl_3$) spectrum of compound **2c**

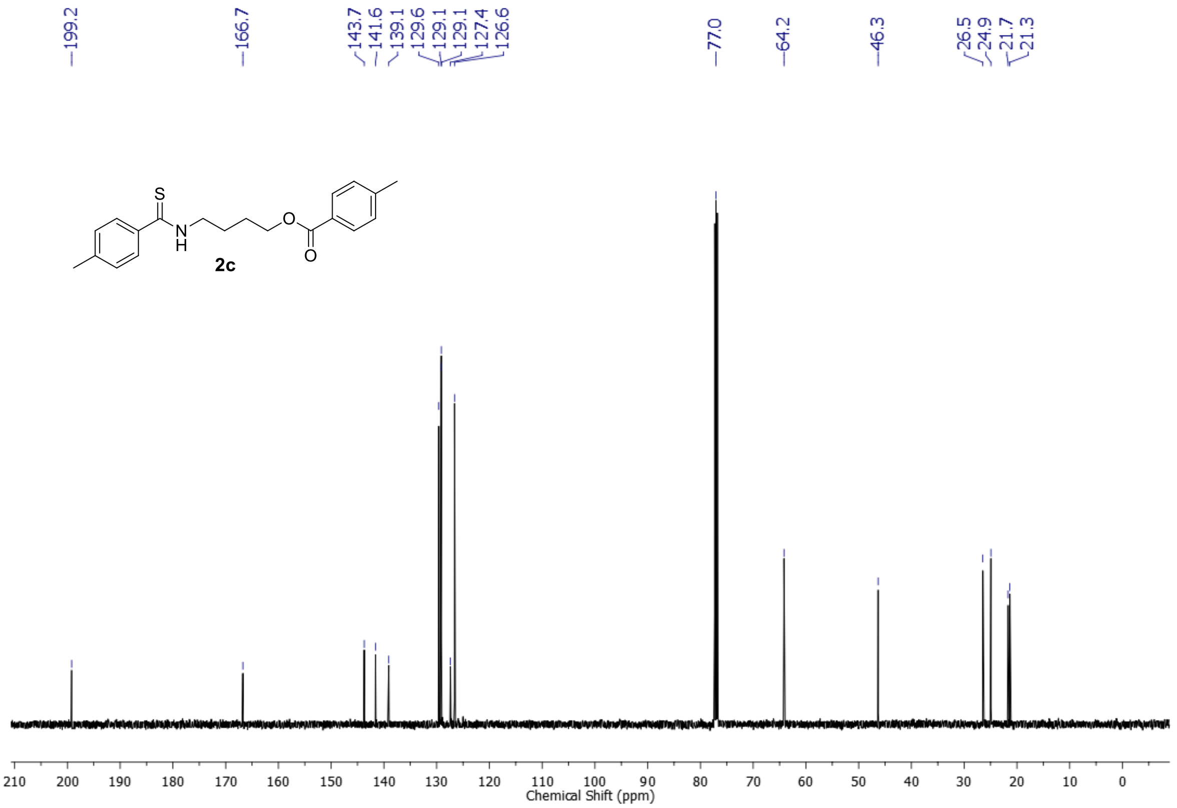

^{1}H NMR (500 MHz, $CDCl_3$) spectrum of compound **2d**

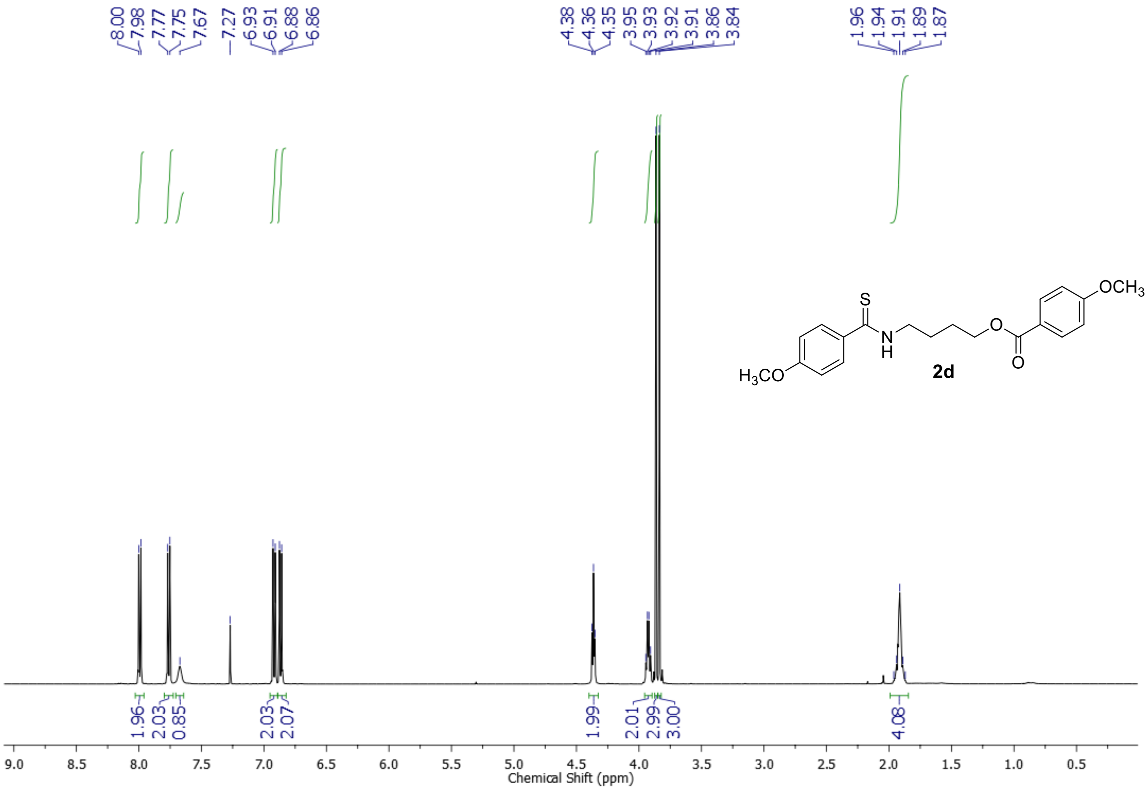

^{13}C NMR (125 MHz, $CDCl_3$) spectrum of compound **2d**

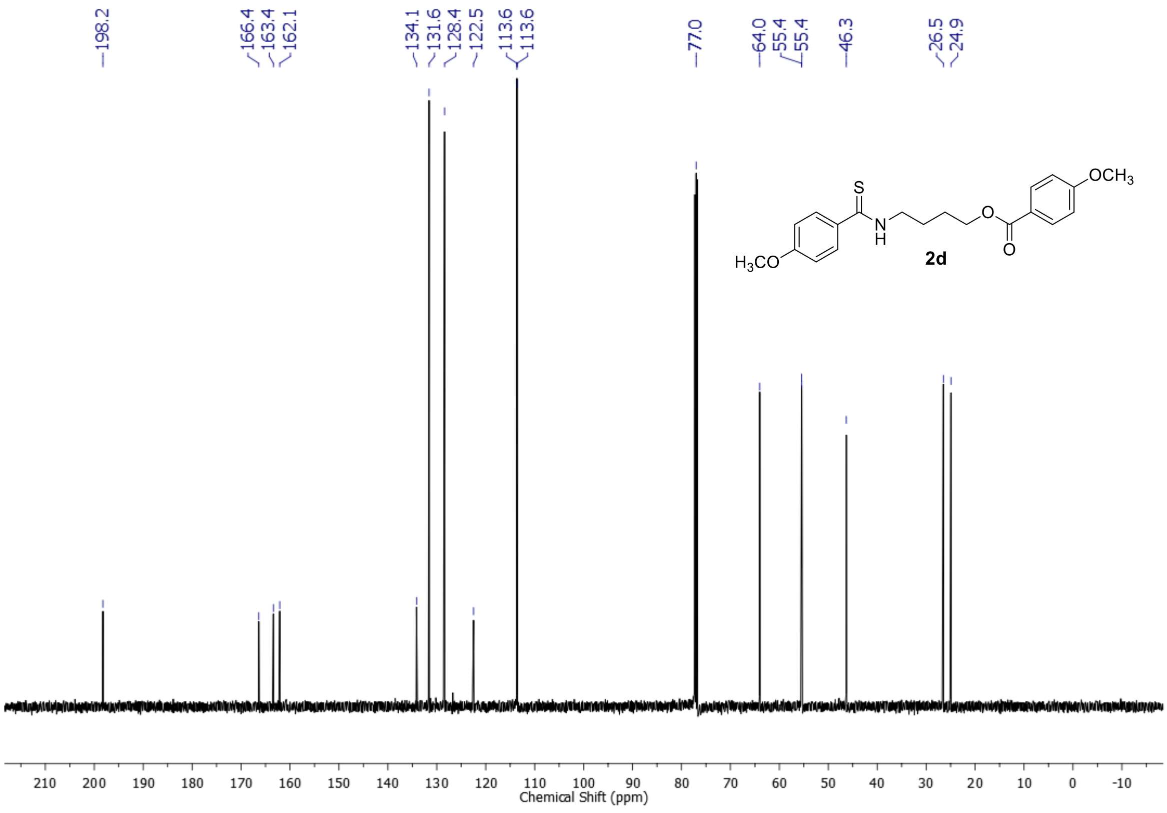

^{1}H NMR (600 MHz, $CDCl_3$) spectrum of compound **2e**

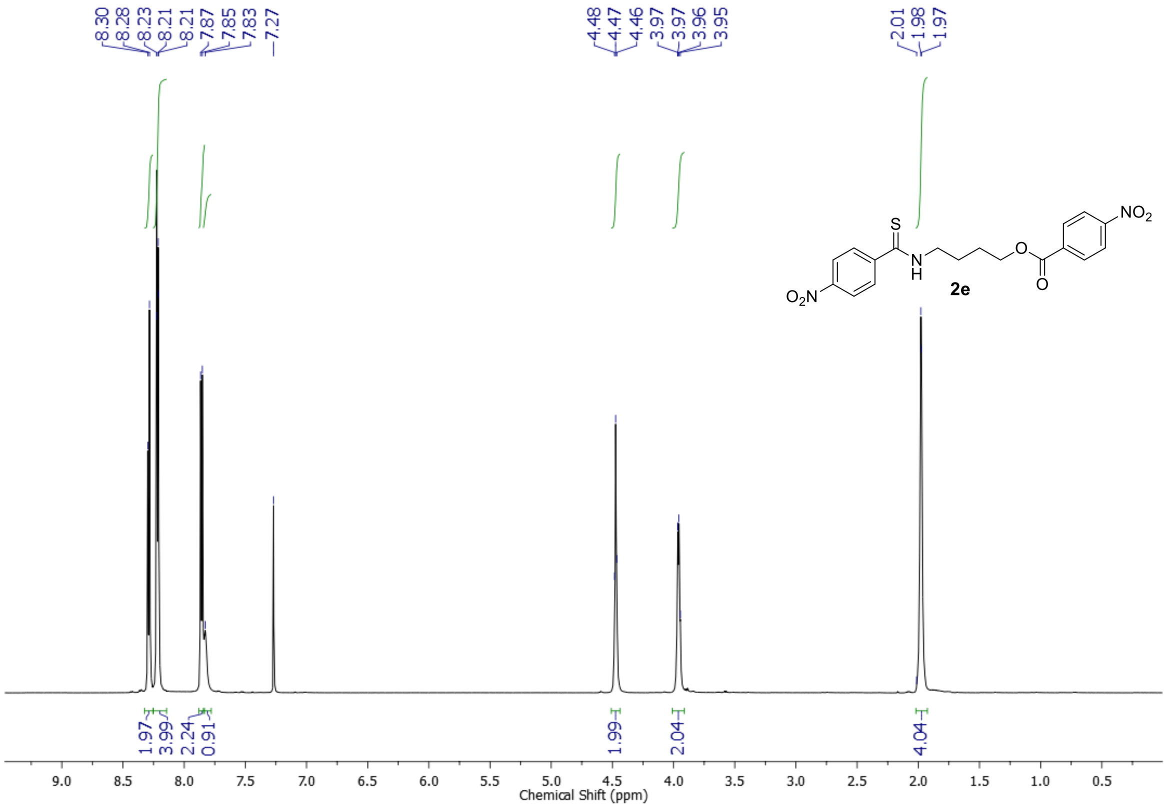

^{13}C NMR (151 MHz, $CDCl_3$) spectrum of compound **2e**

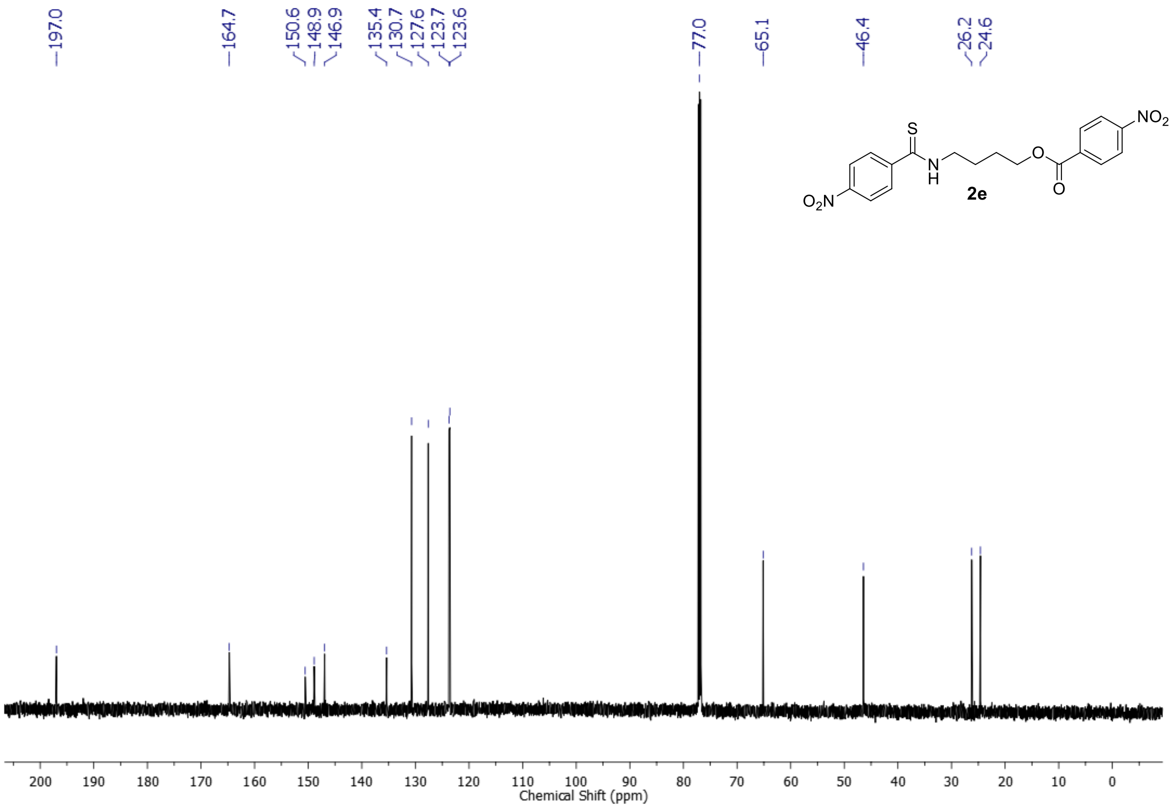

^{1}H NMR (600 MHz, $CDCl_3$) spectrum of compound **2f**

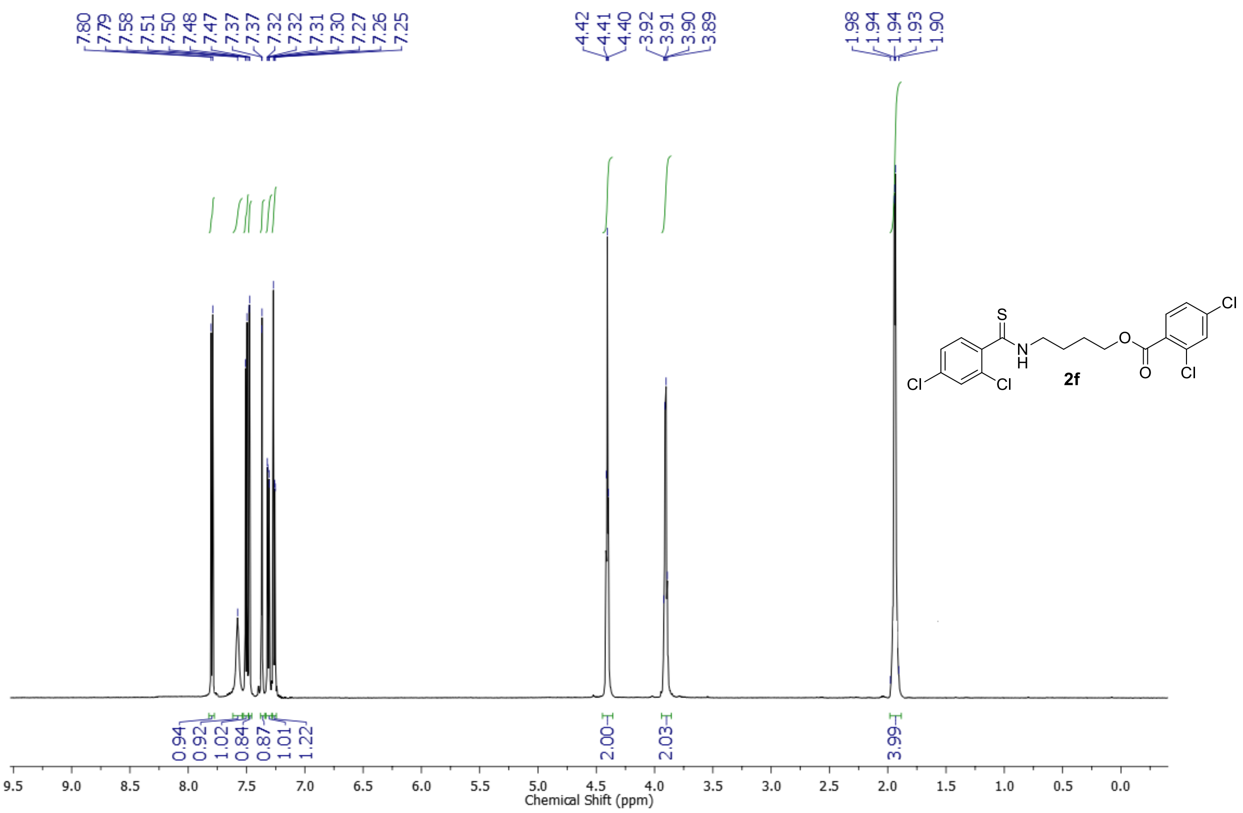

^{13}C NMR (151 MHz, $CDCl_3$) spectrum of compound **2f**

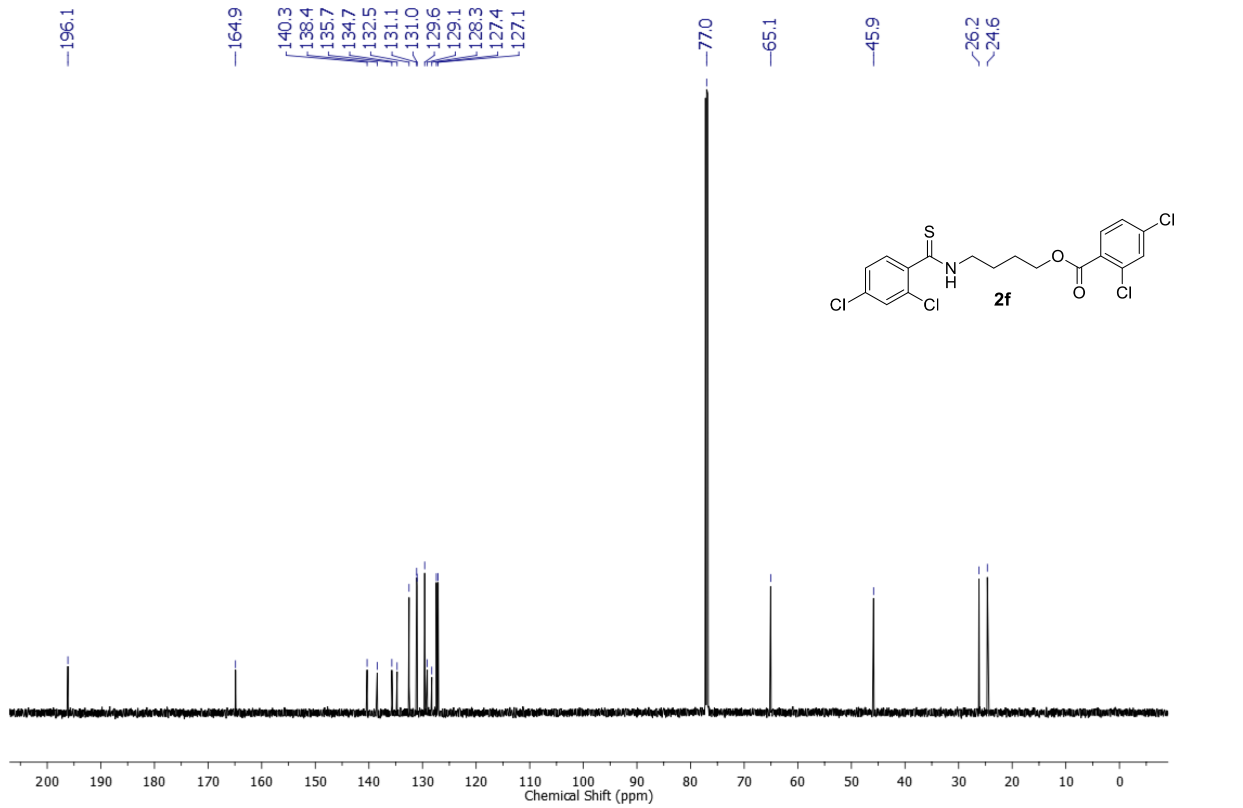

^{1}H NMR (500 MHz, $CDCl_3$) spectrum of compound **2g**

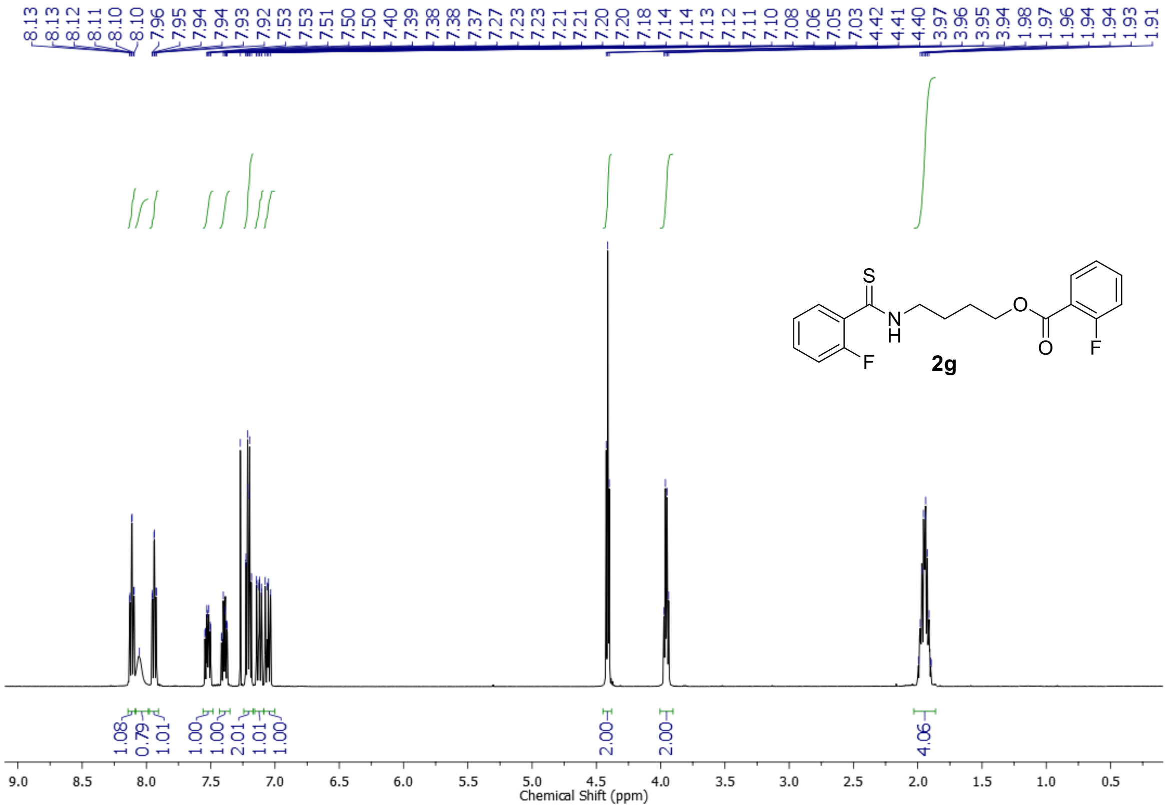

^{13}C NMR (125 MHz, $CDCl_3$) spectrum of compound **2g**

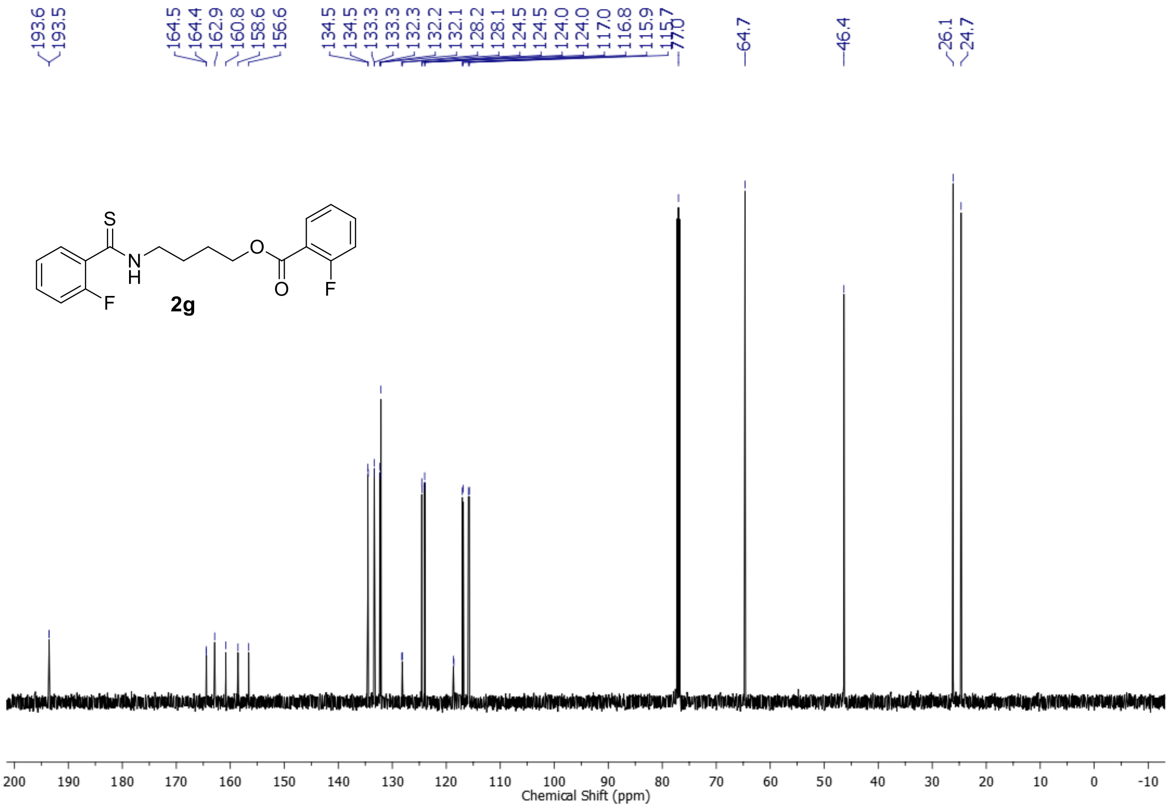

^{1}H NMR (500 MHz, $CDCl_3$) spectrum of compound **2h**

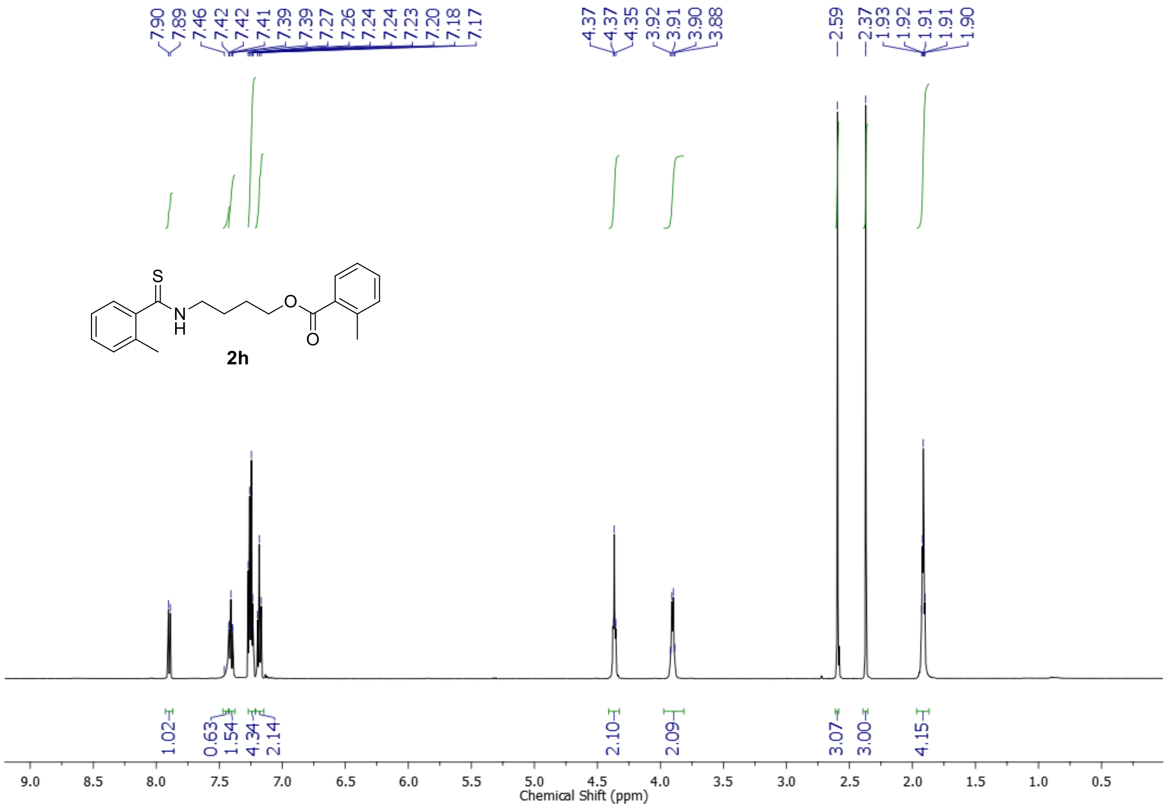

^{13}C NMR (125 MHz, $CDCl_3$) spectrum of compound **2h**

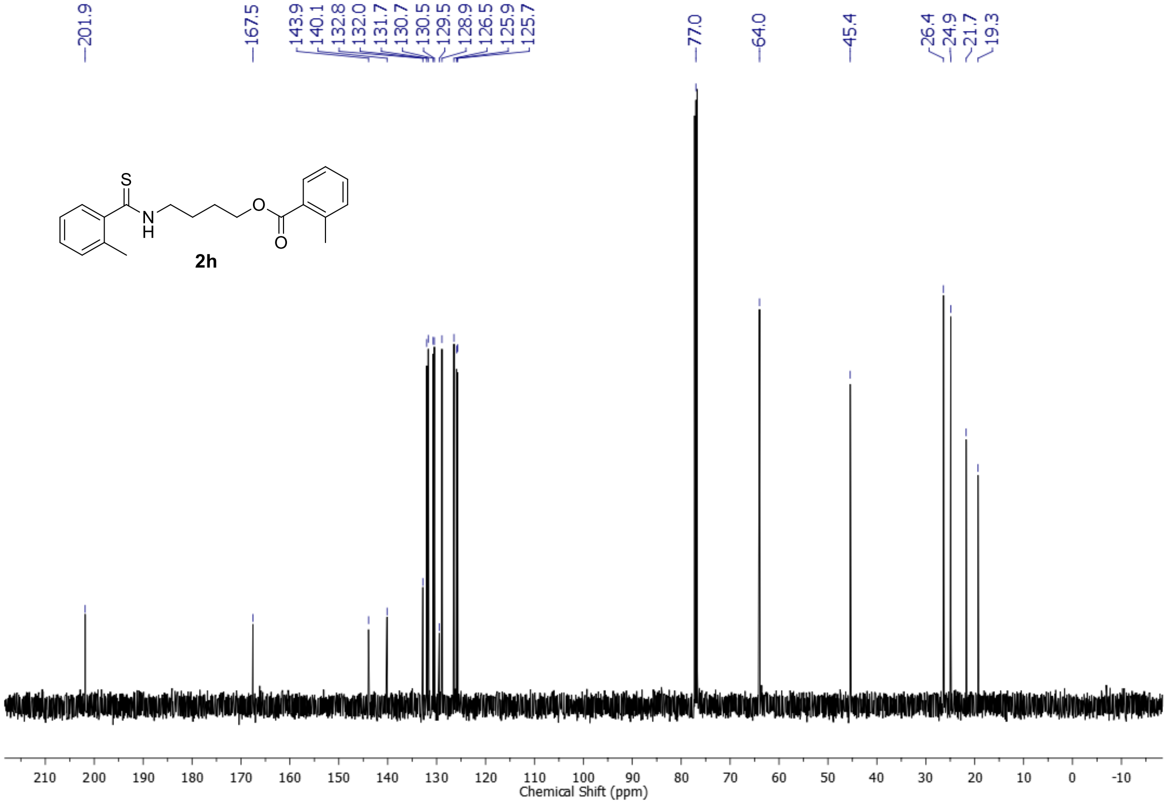

^{1}H NMR (600 MHz, $CDCl_3$) spectrum of compound **2i**

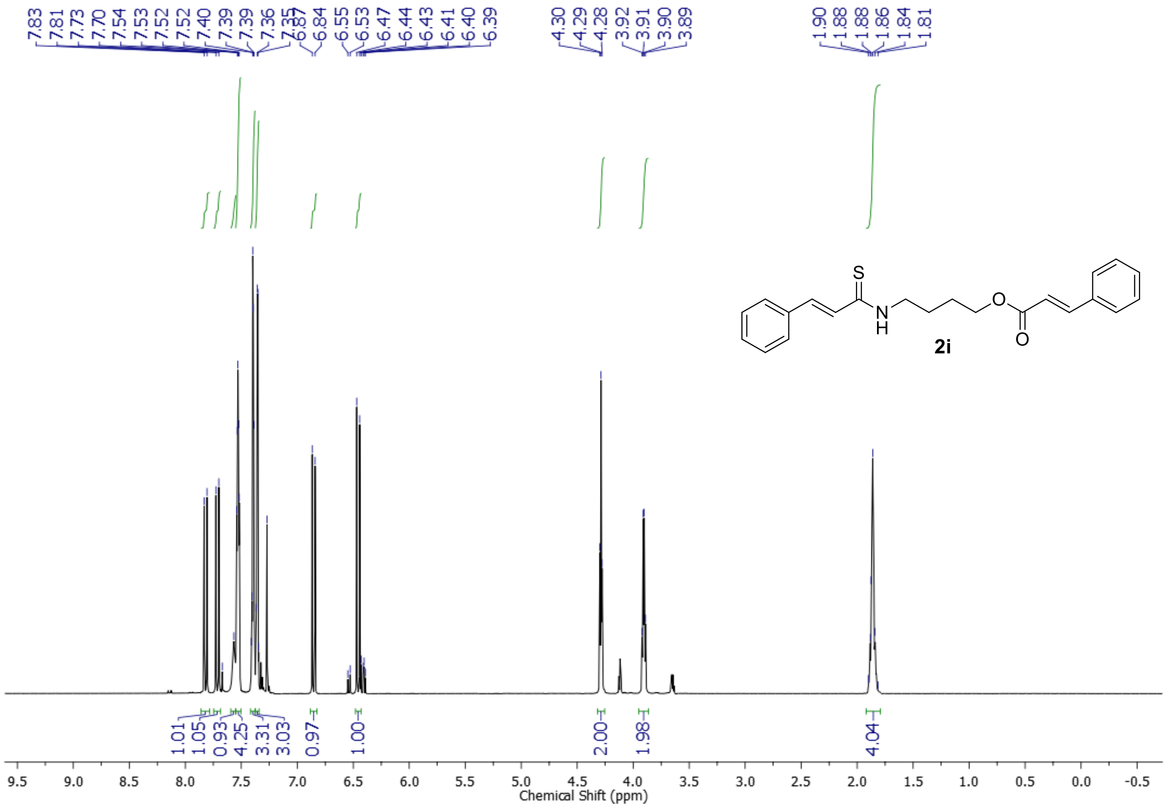

^{13}C NMR (151 MHz, $CDCl_3$) spectrum of compound **2i**

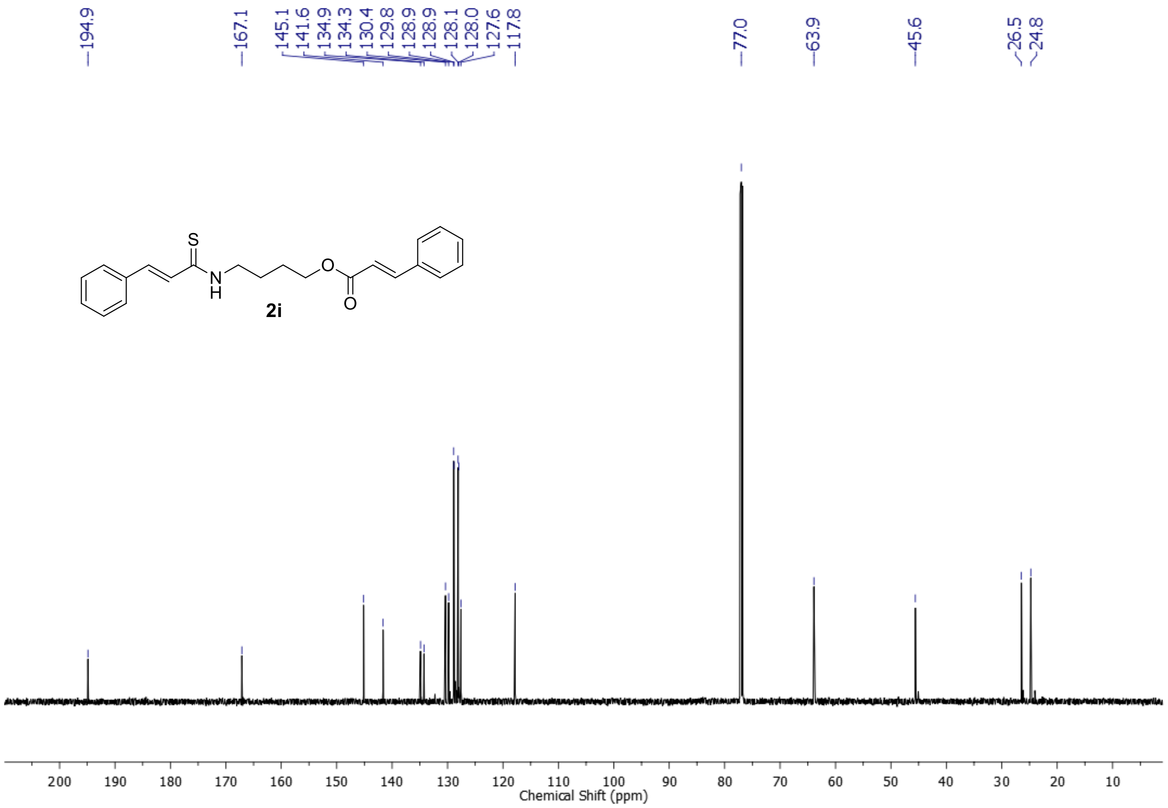

^{1}H NMR (600 MHz, $CDCl_3$) spectrum of compound **2j**

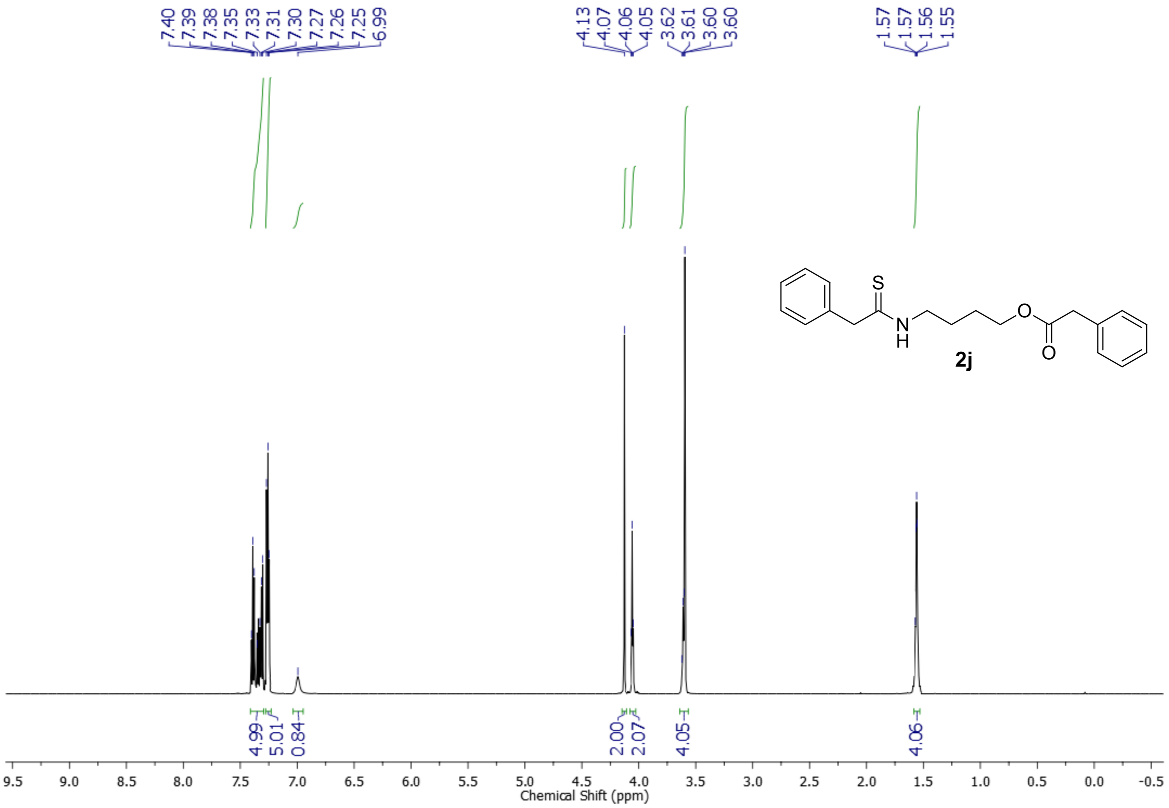

^{13}C NMR (151 MHz, $CDCl_3$) spectrum of compound **2j**

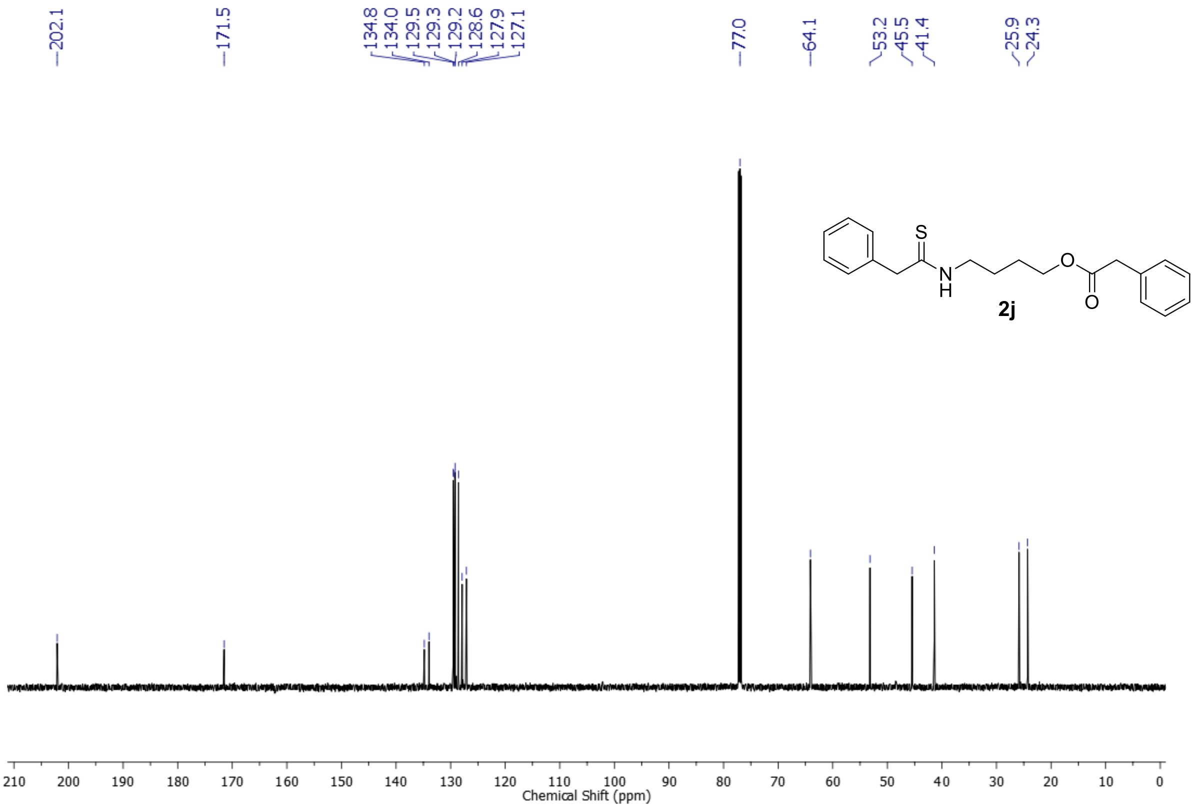

^{1}H NMR (600 MHz, $CDCl_3$) spectrum of compound **2k**

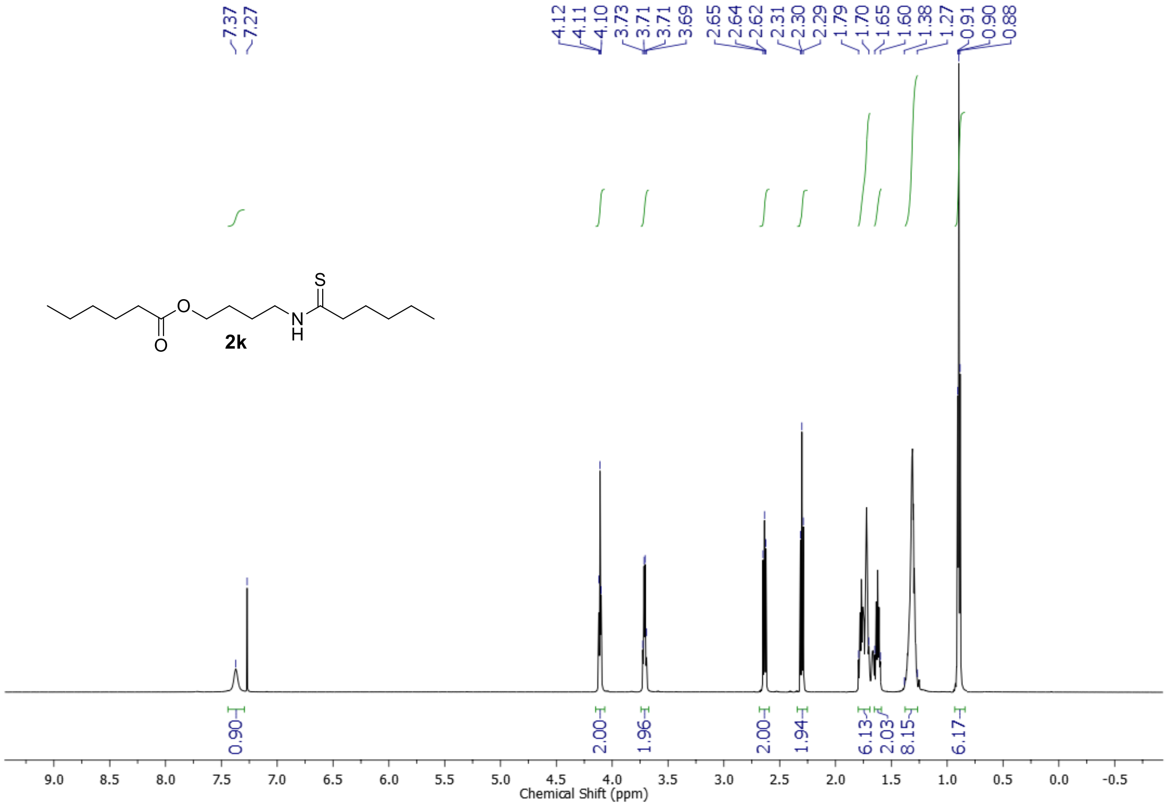

^{13}C NMR (151 MHz, $CDCl_3$) spectrum of compound **2k**

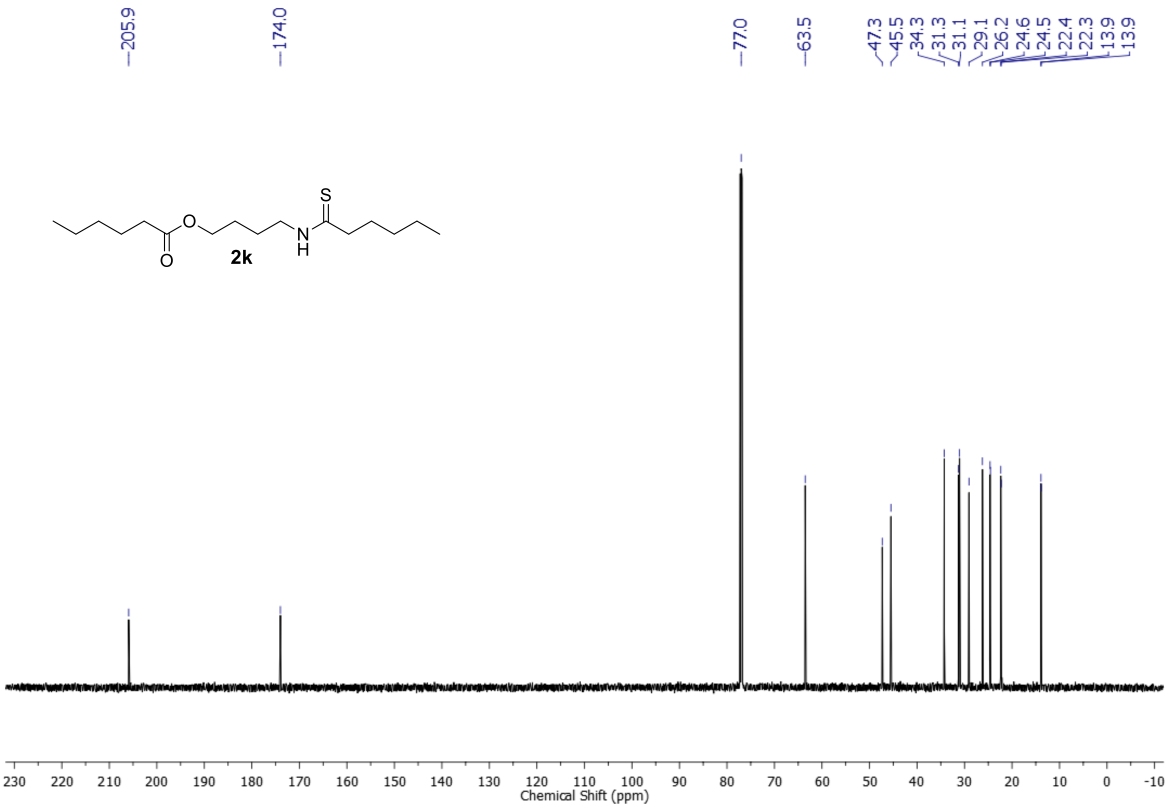

^{1}H NMR (600 MHz, $CDCl_3$) spectrum of compound **2l**

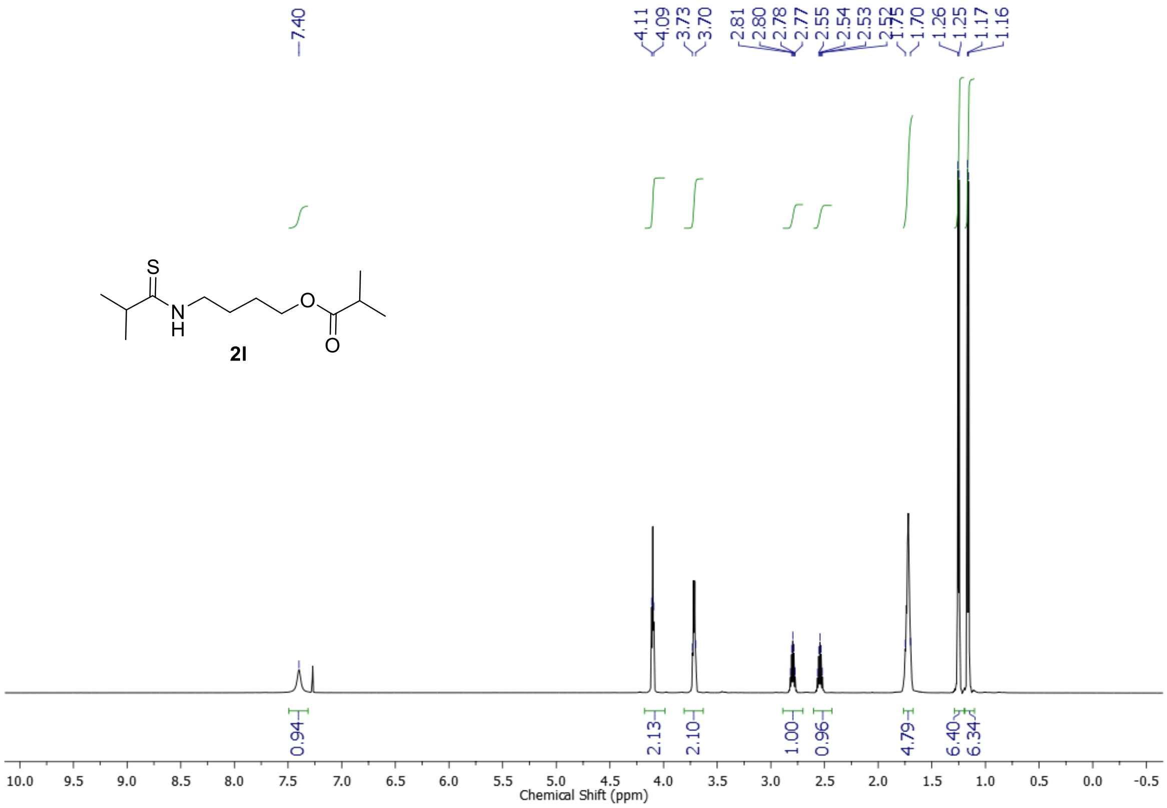

^{13}C NMR (151 MHz, $CDCl_3$) spectrum of compound **2l**

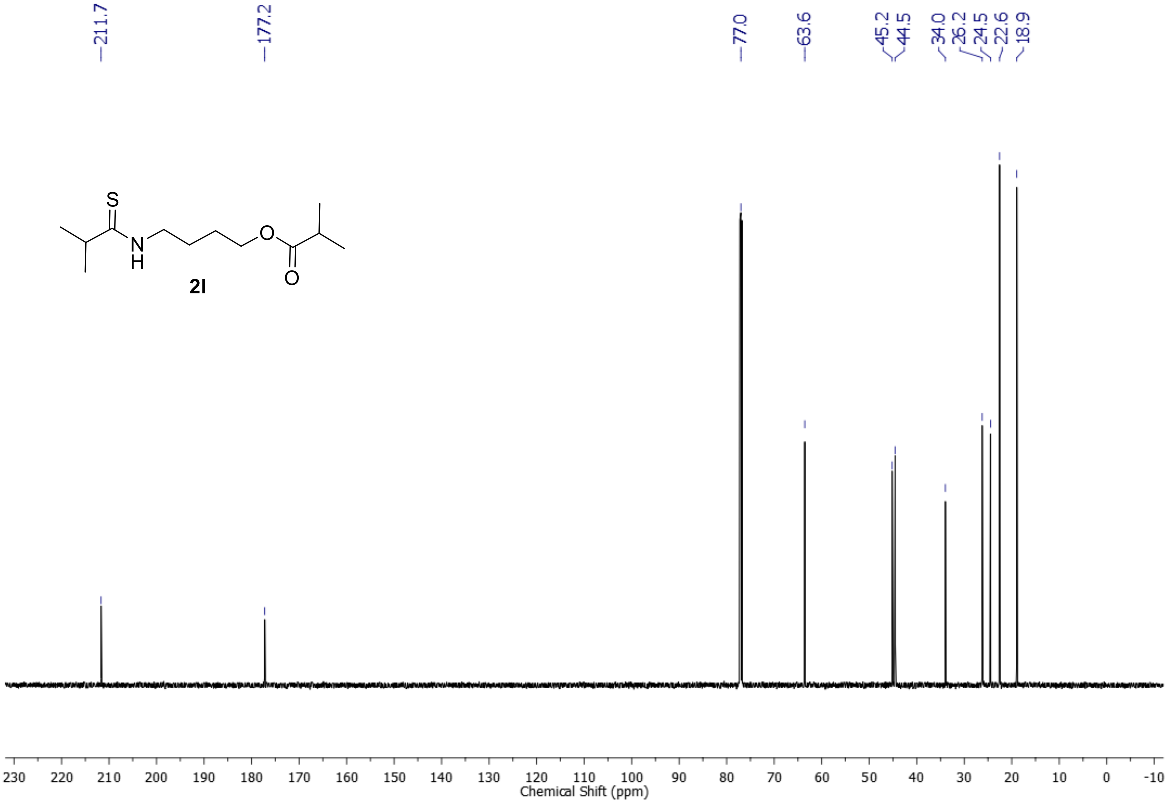

^{1}H NMR (600 MHz, $CDCl_3$) spectrum of compound **2m**

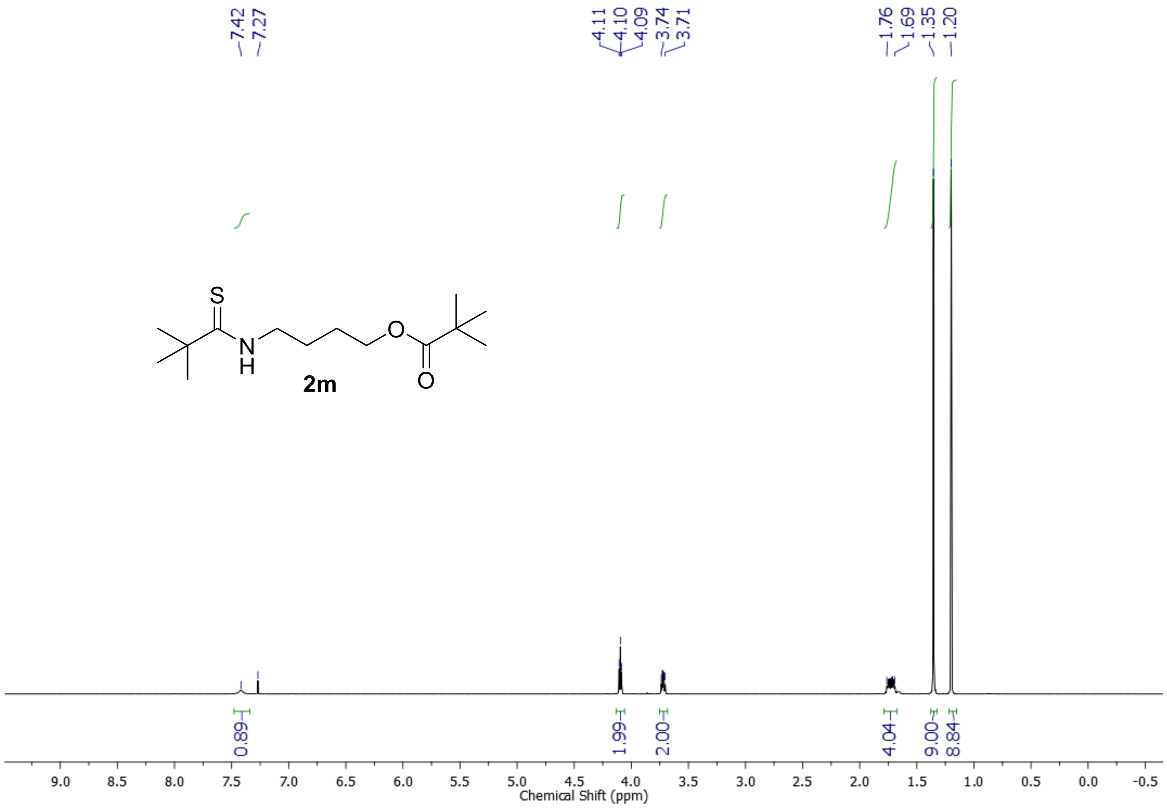

^{13}C NMR (151 MHz, $CDCl_3$) spectrum of compound **2m**

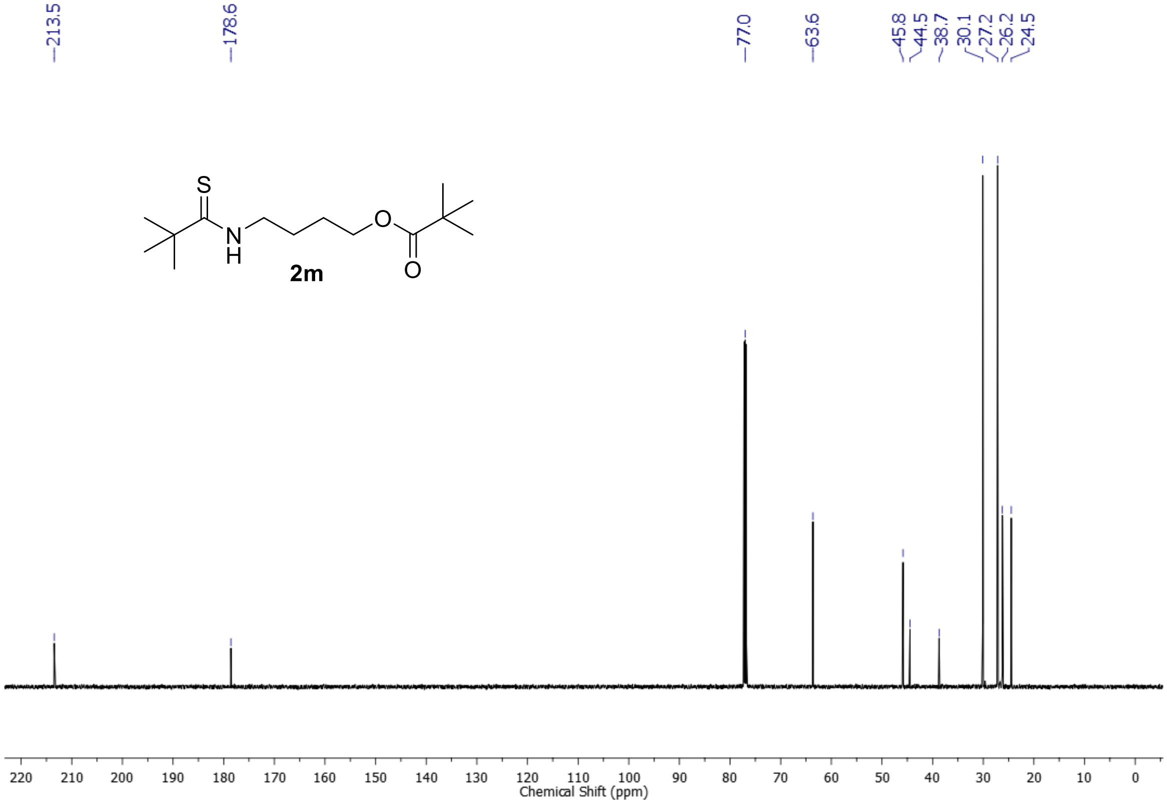

^{1}H NMR (600 MHz, $CDCl_3$) spectrum of compound **3a**

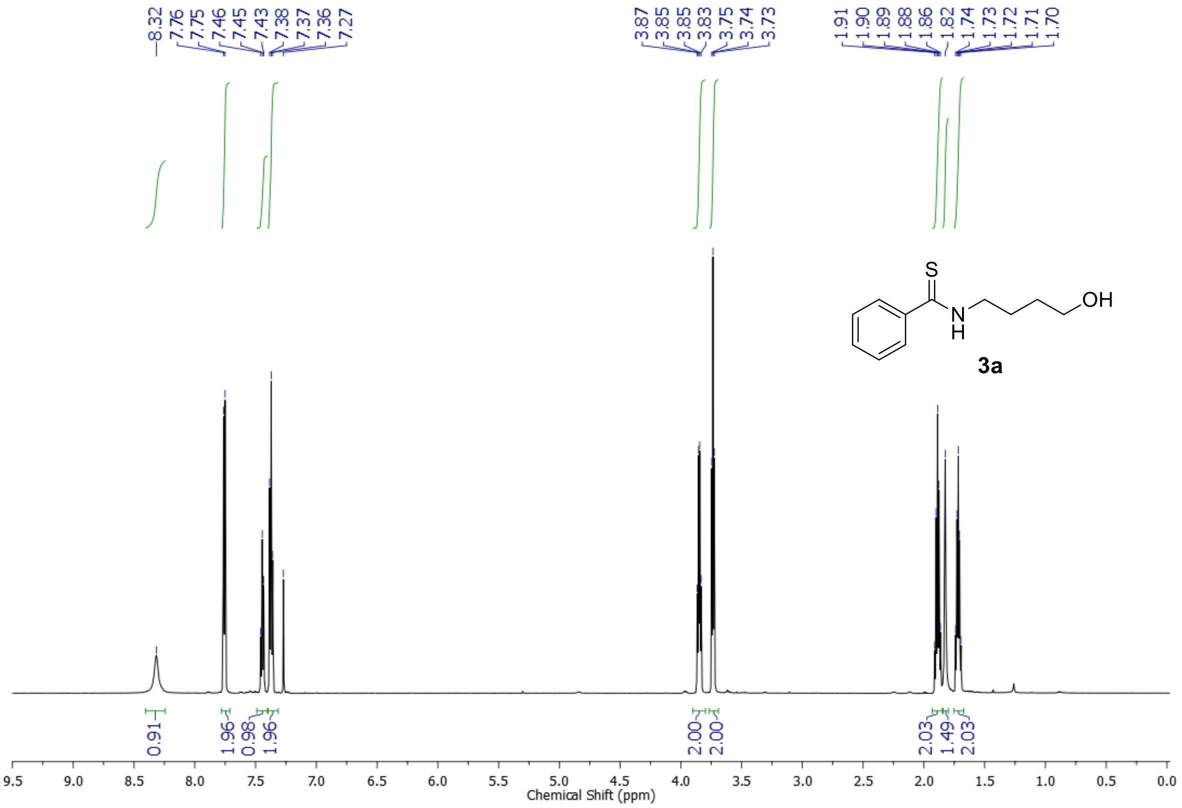

^{1}H NMR (500 MHz, $CDCl_3$) spectrum of compound **3b**

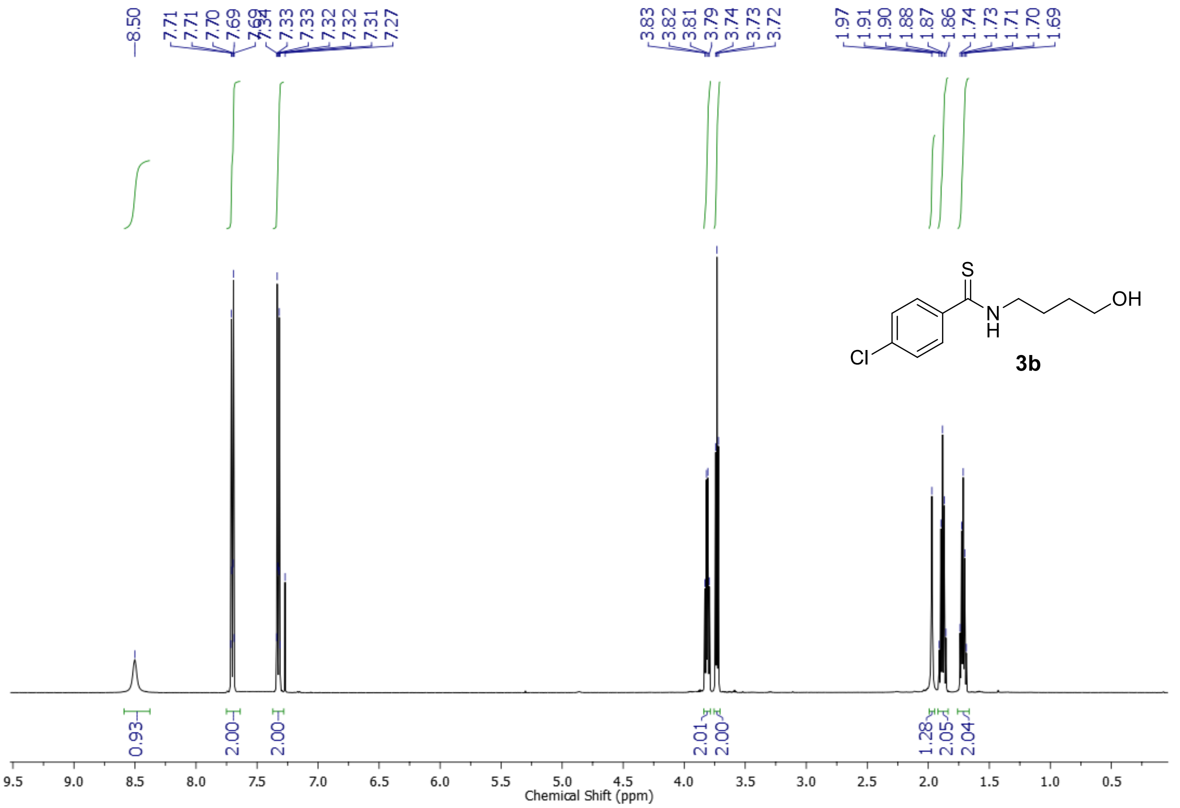

^{13}C NMR (125 MHz, $CDCl_3$) spectrum of compound **3b**

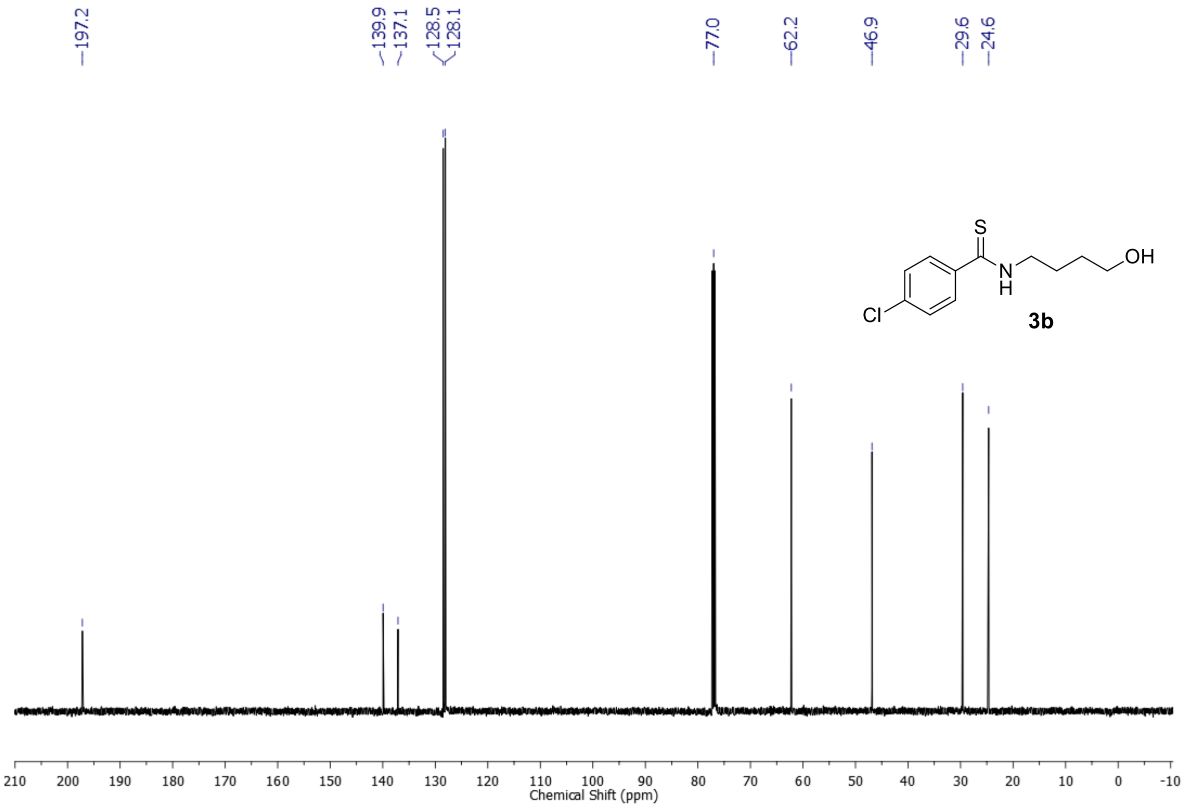

^{1}H NMR (500 MHz, $CDCl_3$) spectrum of compound **3c**

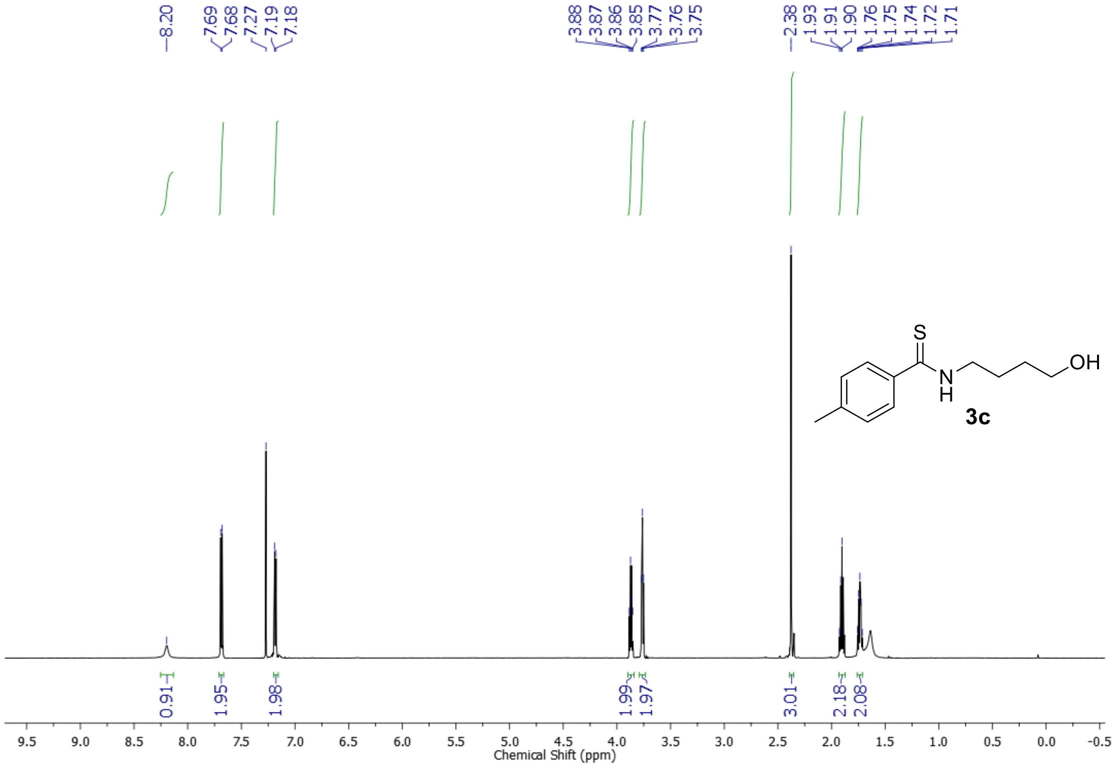

^{13}C NMR (125 MHz, $CDCl_3$) spectrum of compound **3c**

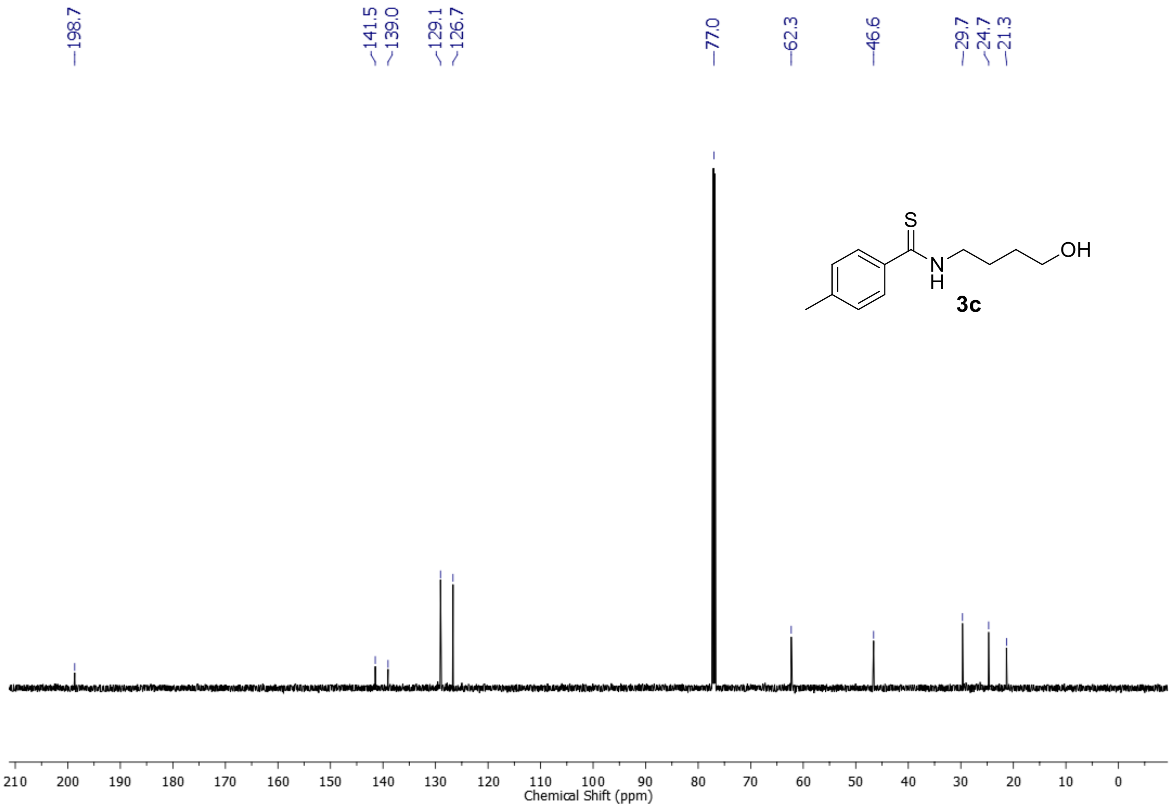

^{1}H NMR (500 MHz, $CDCl_3$) spectrum of compound **3d**

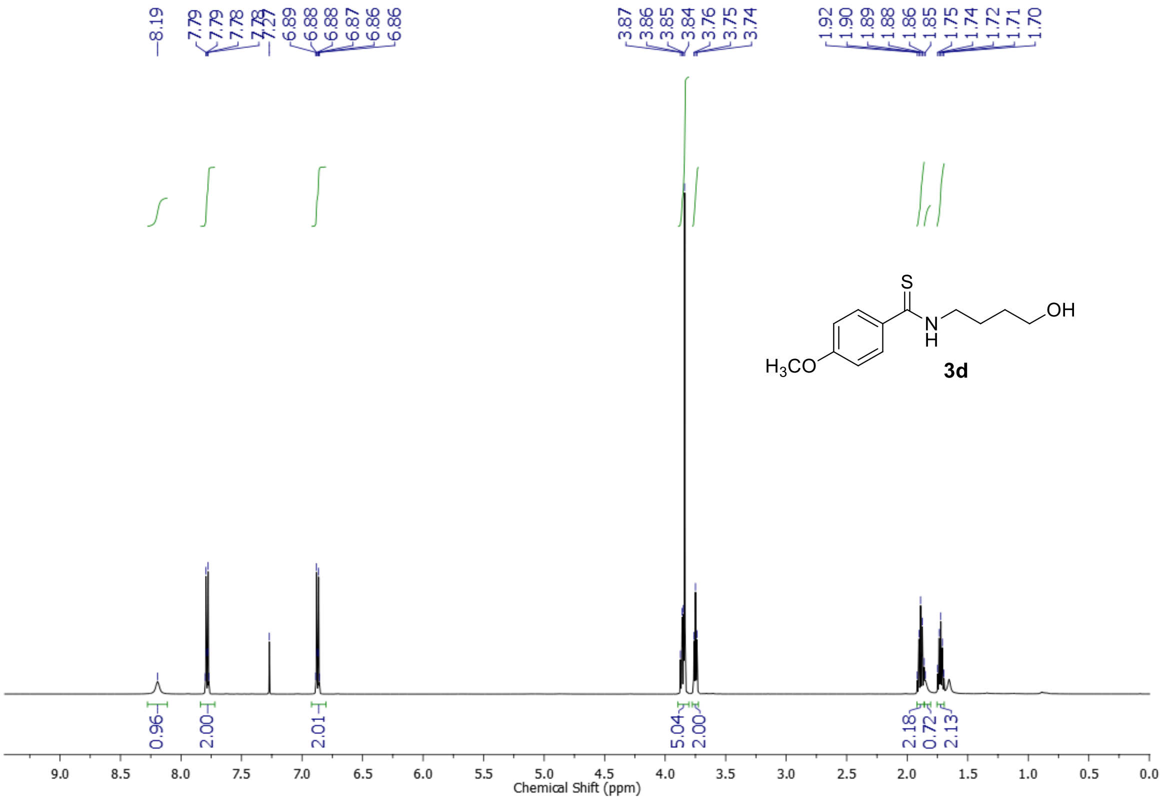

^{13}C NMR (125 MHz, $CDCl_3$) spectrum of compound **3d**

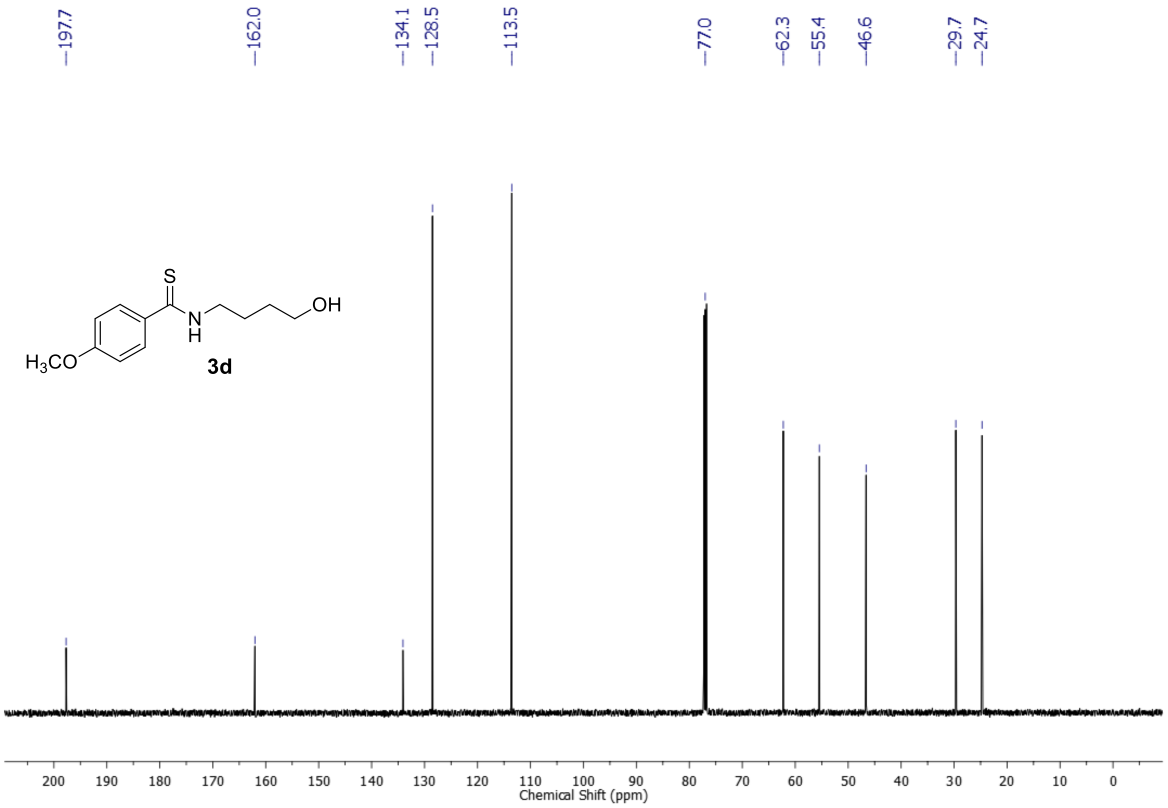

^{1}H NMR (500 MHz, $CDCl_3$) spectrum of compound **3e**

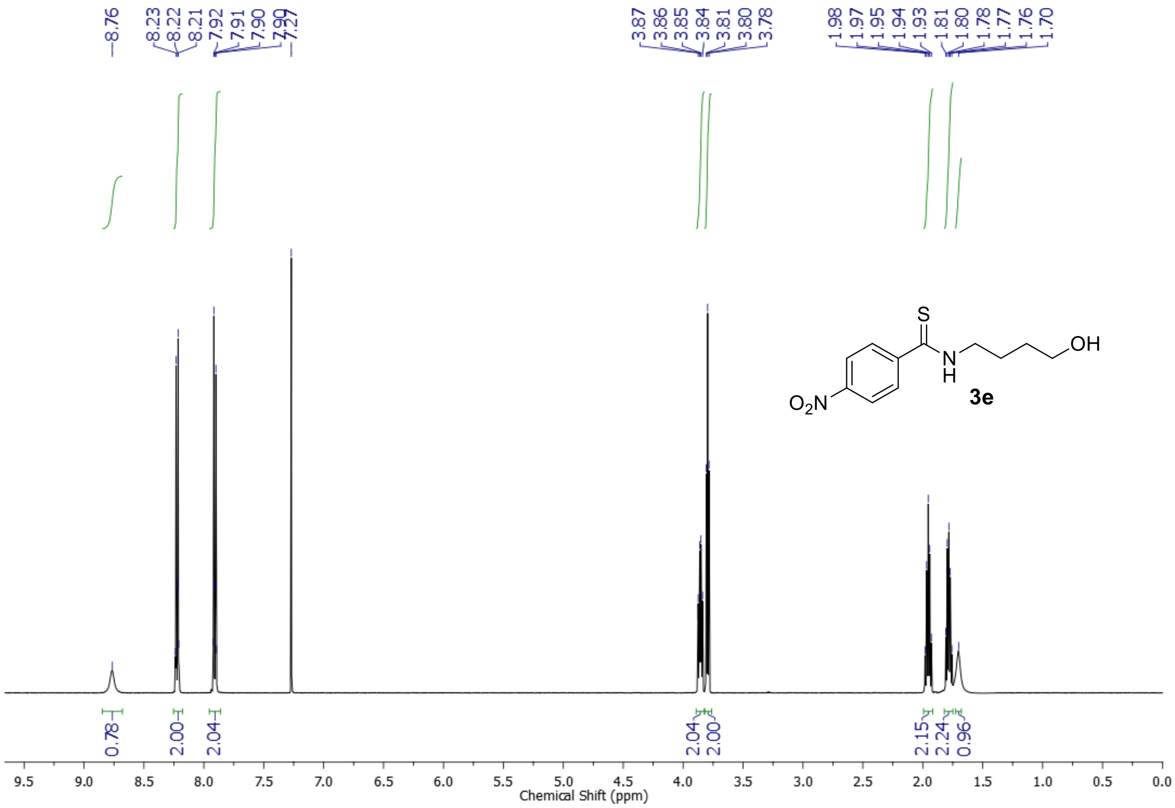

^{13}C NMR (125 MHz, $CDCl_3$) spectrum of compound **3e**

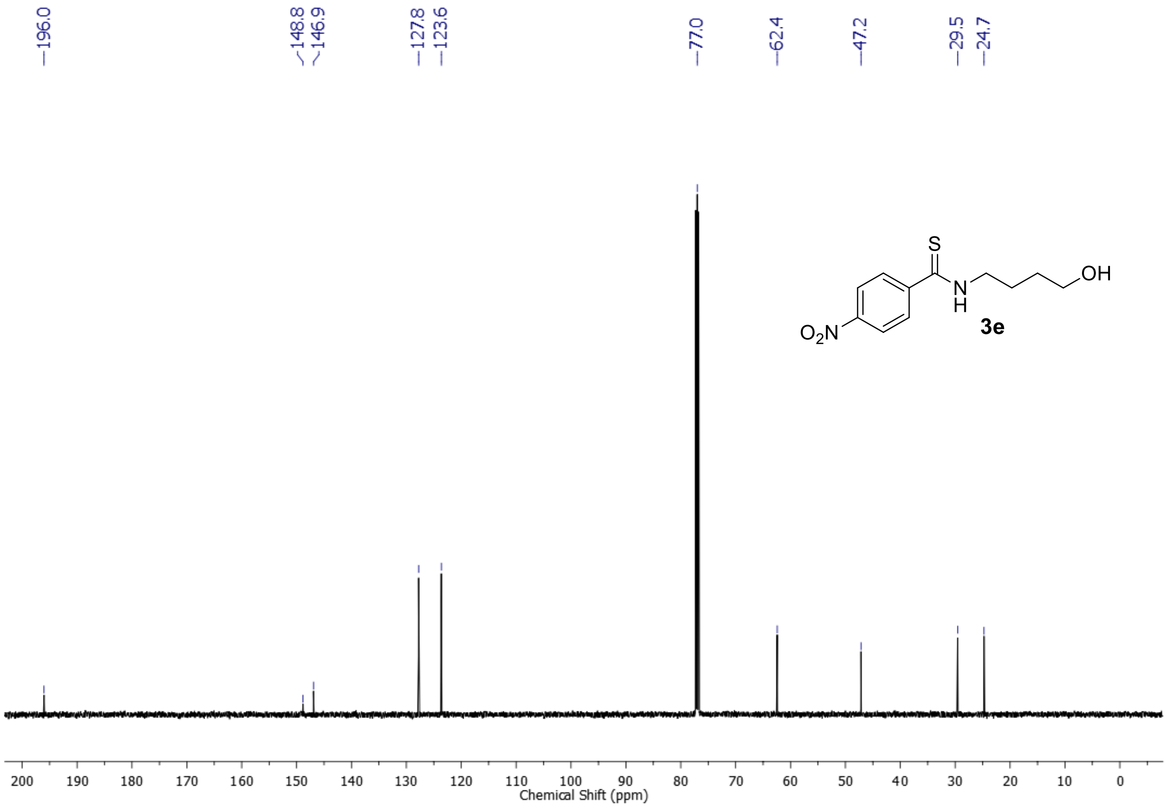

^{1}H NMR (600 MHz, $CDCl_3$) spectrum of compound **3f**

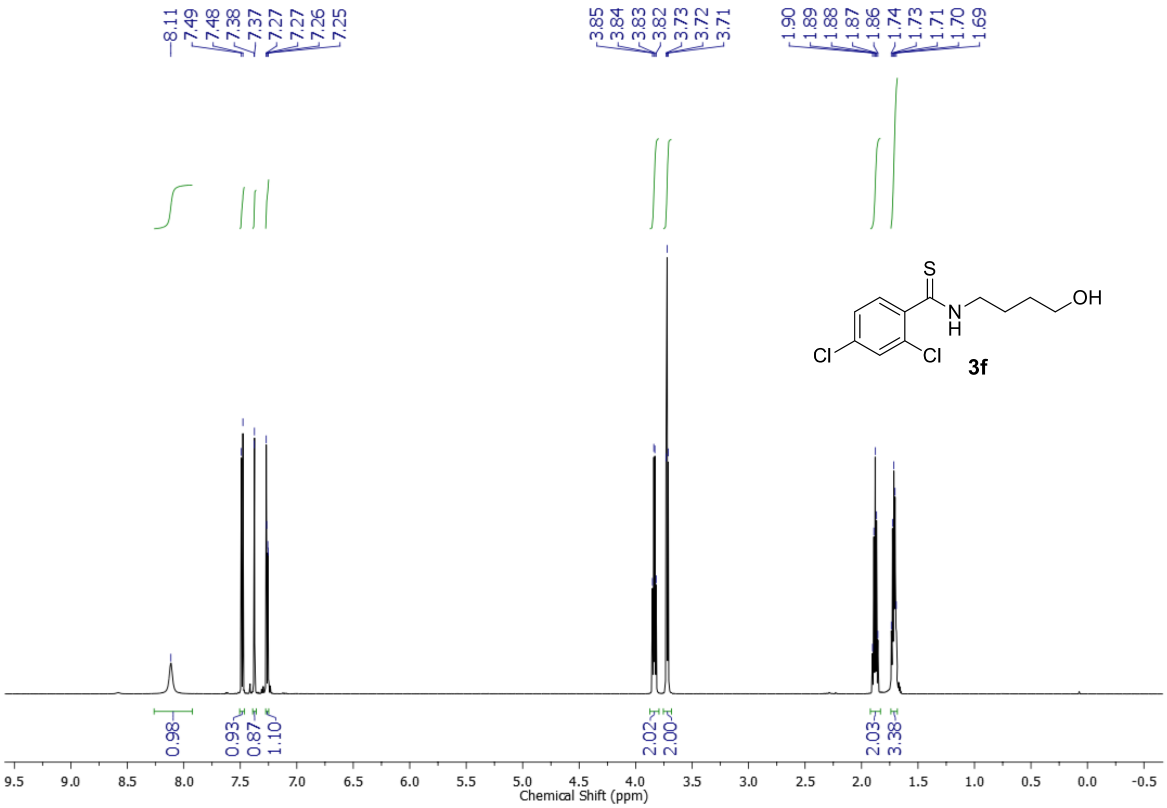

^{13}C NMR (151 MHz, $CDCl_3$) spectrum of compound **3f**

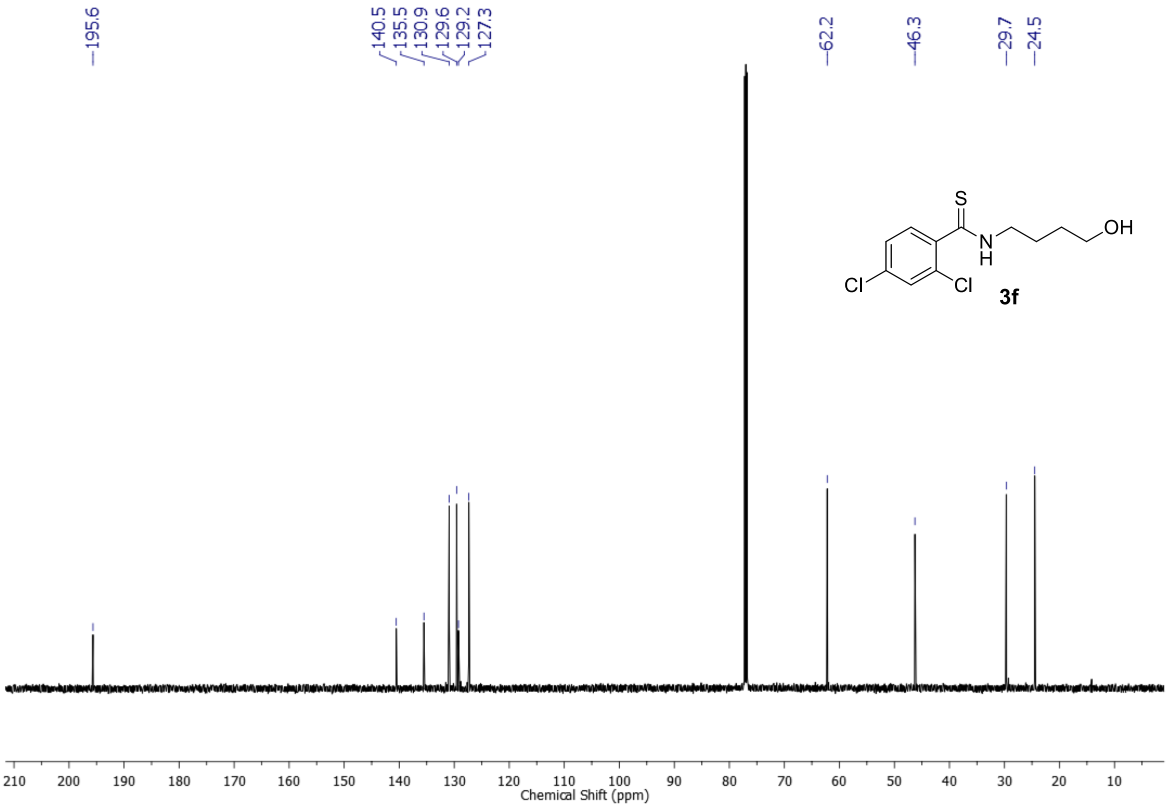

^{1}H NMR (500 MHz, $CDCl_3$) spectrum of compound **3g**

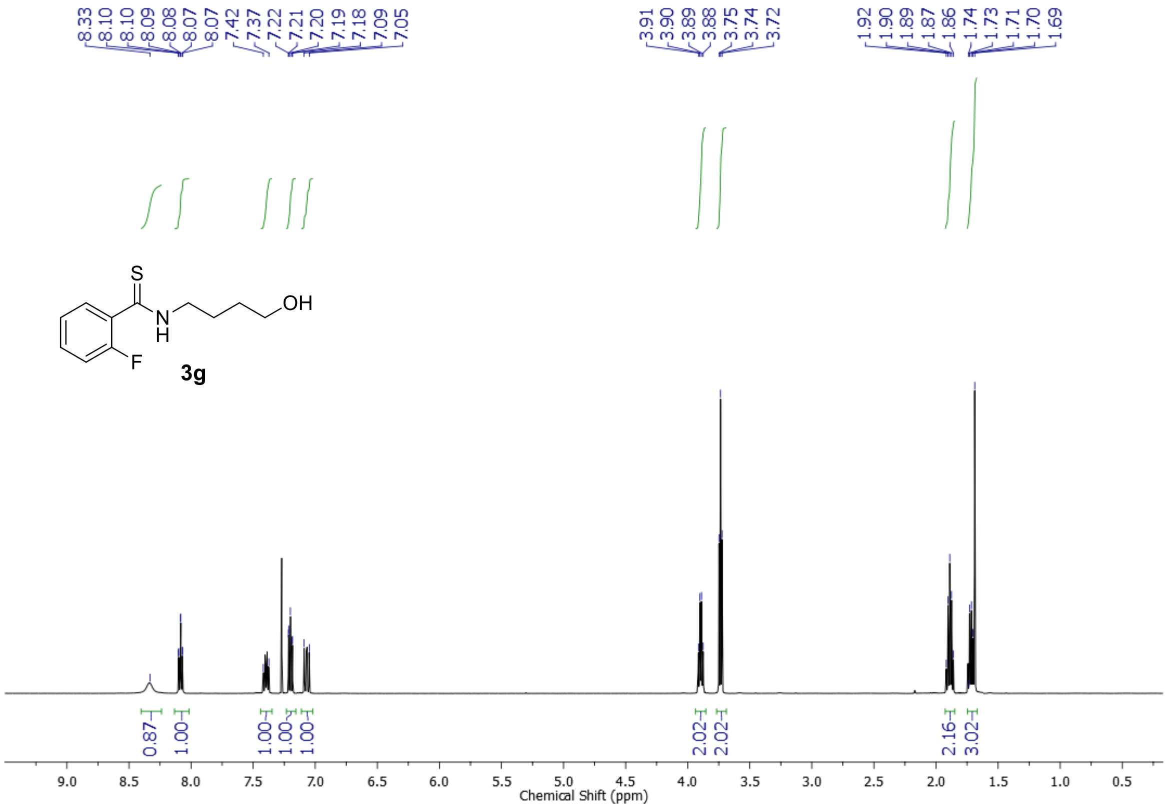

^{13}C NMR (125 MHz, $CDCl_3$) spectrum of compound **3g**

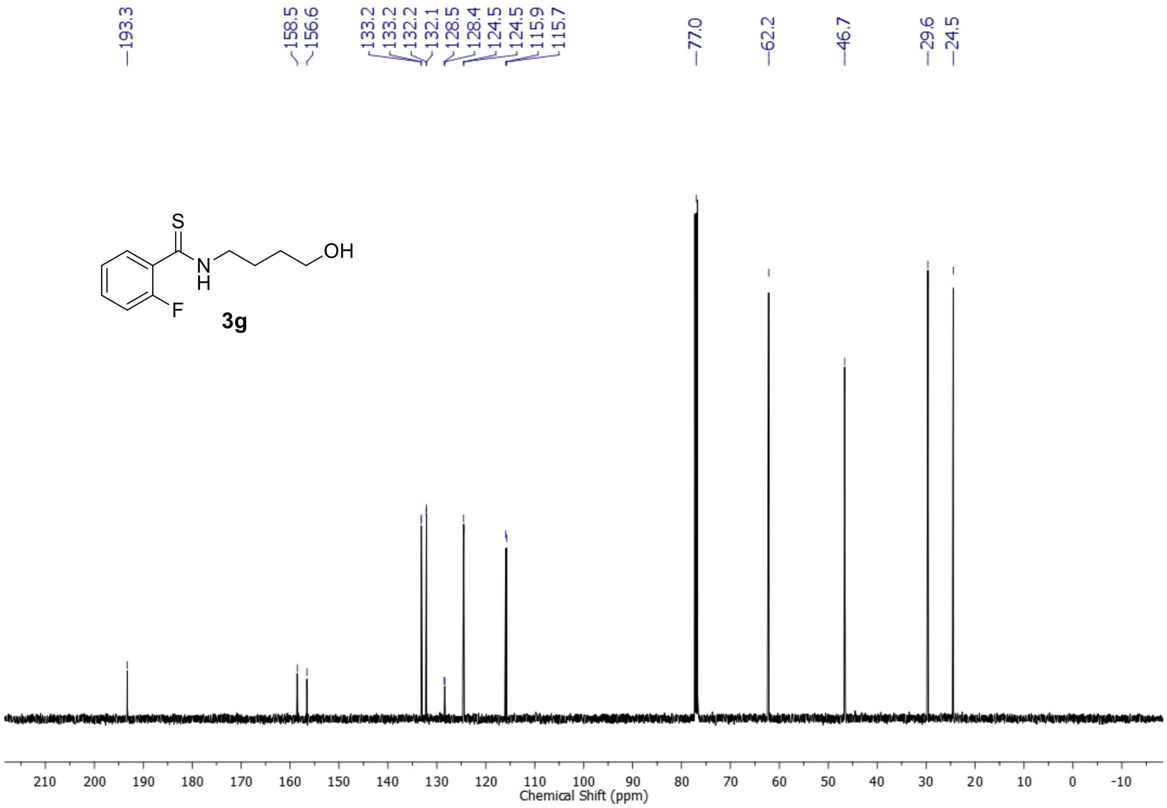

^{1}H NMR (500 MHz, $CDCl_3$) spectrum of compound **3h**

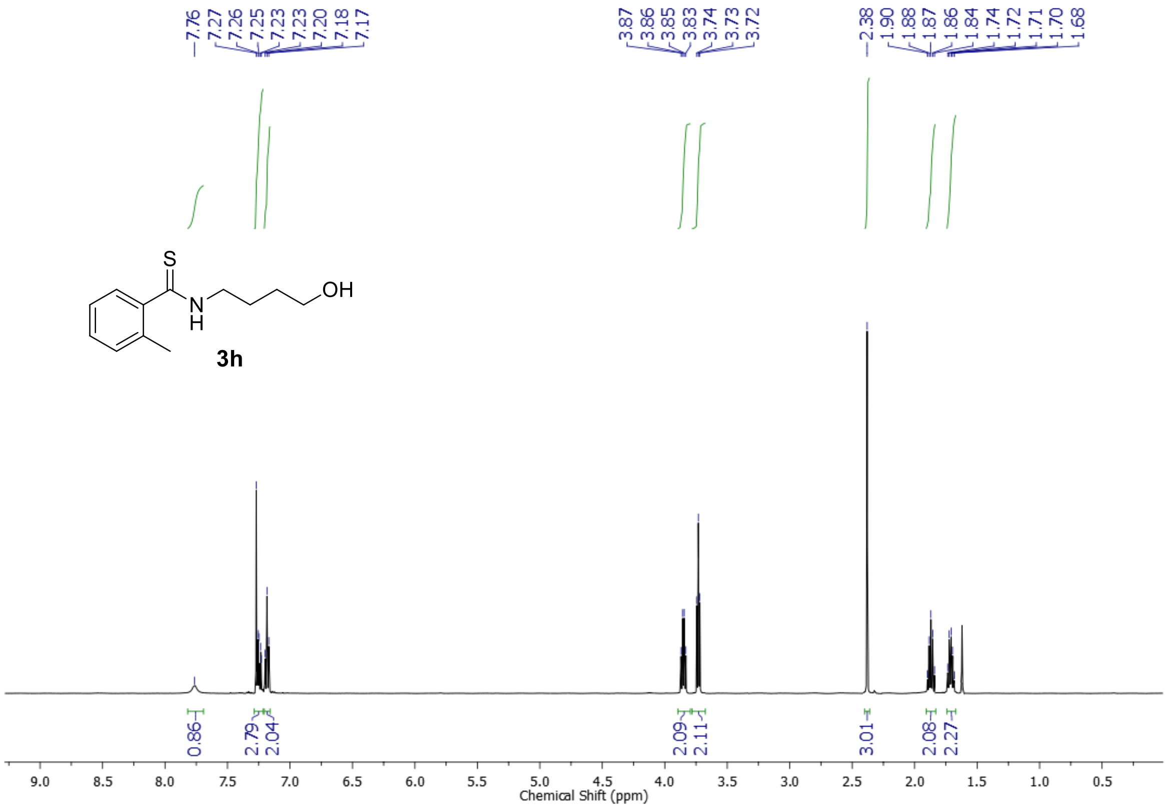

^{13}C NMR (125 MHz, $CDCl_3$) spectrum of compound **3h**

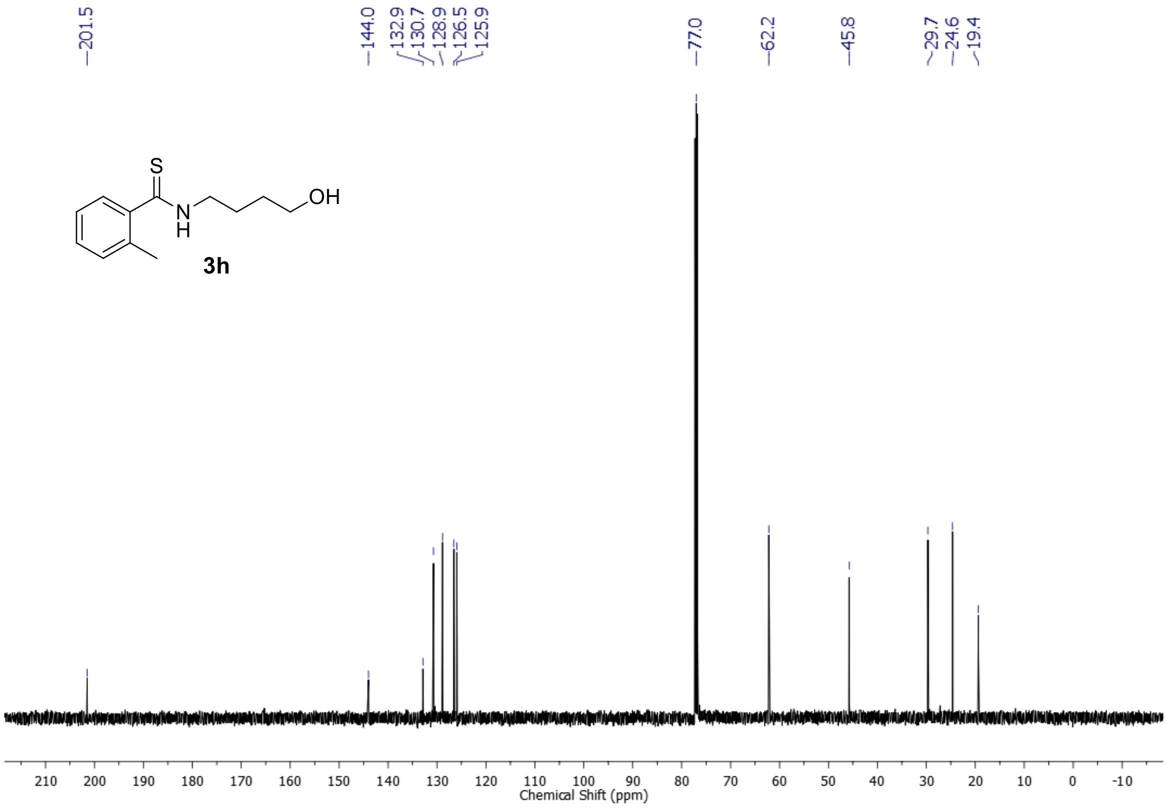

^{1}H NMR (600 MHz, $CDCl_3$) spectrum of compound **3i**

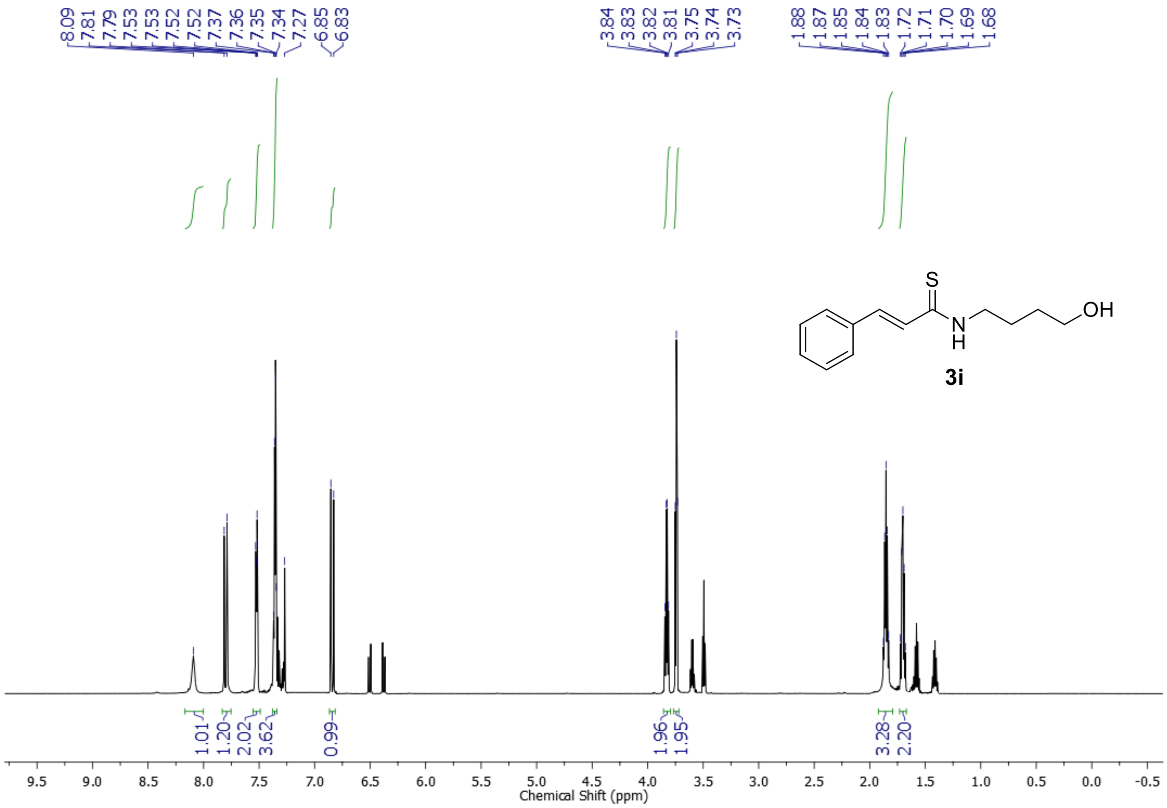

^{13}C NMR (151 MHz, $CDCl_3$) spectrum of compound **3i**

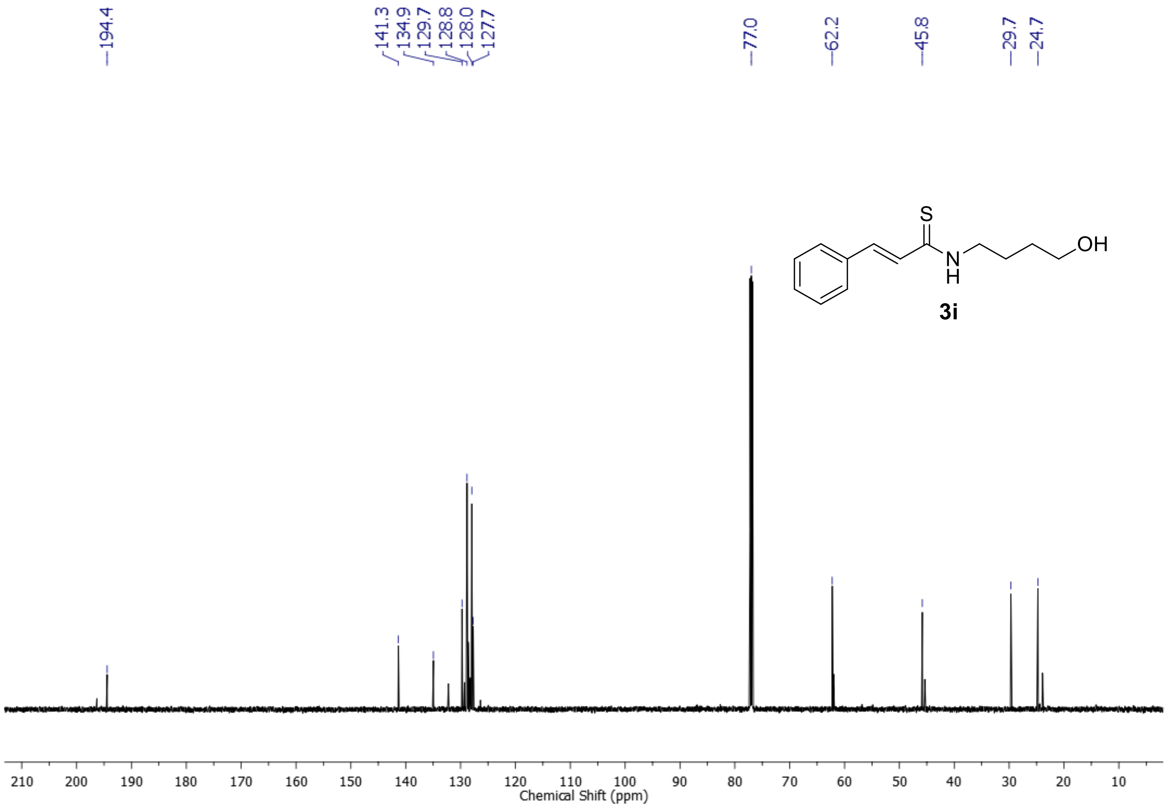

^{1}H NMR (600 MHz, $CDCl_3$) spectrum of compound **3j**

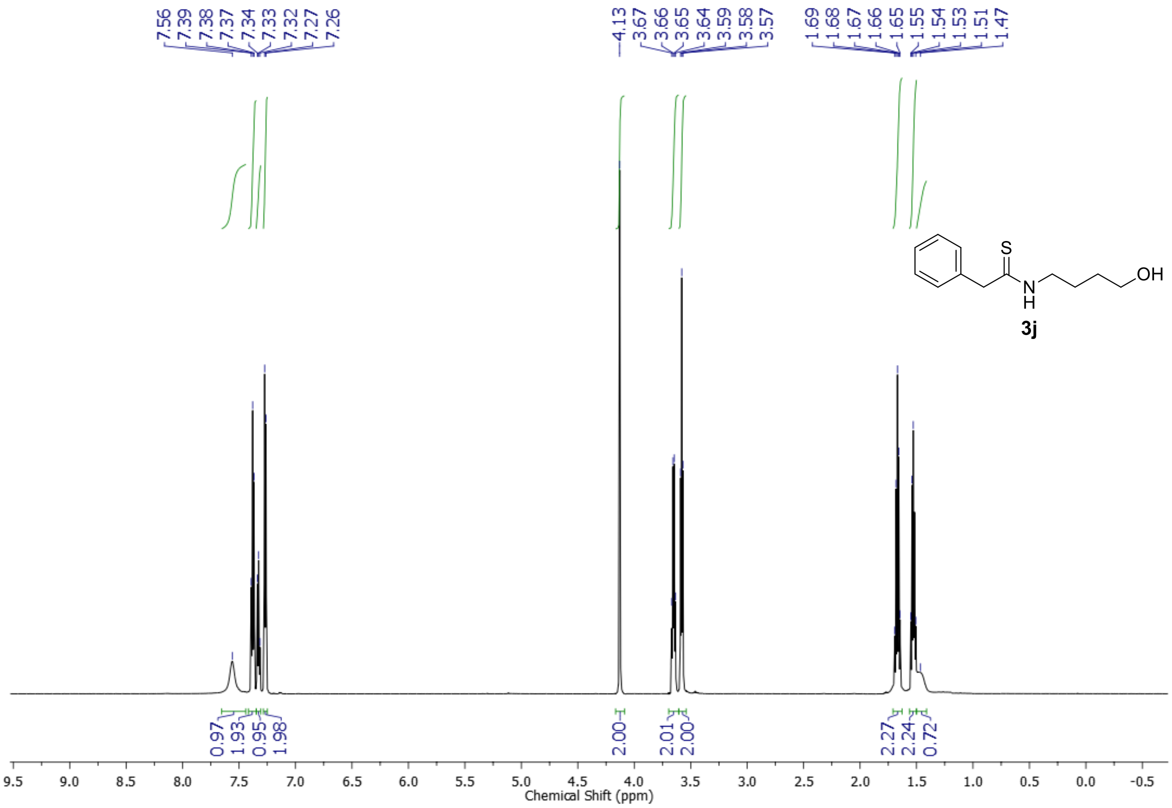

^{1}H NMR (600 MHz, $CDCl_3$) spectrum of compound **3k**

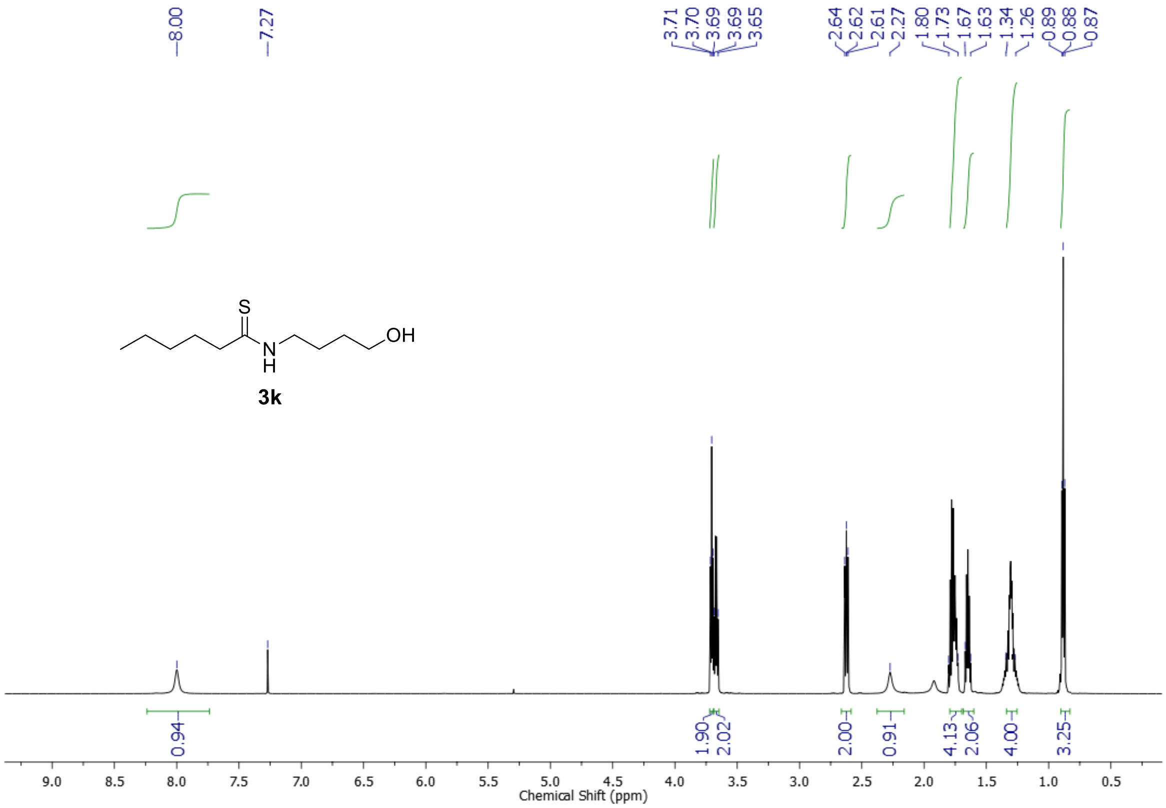

^{13}C NMR (151 MHz, $CDCl_3$) spectrum of compound **3k**

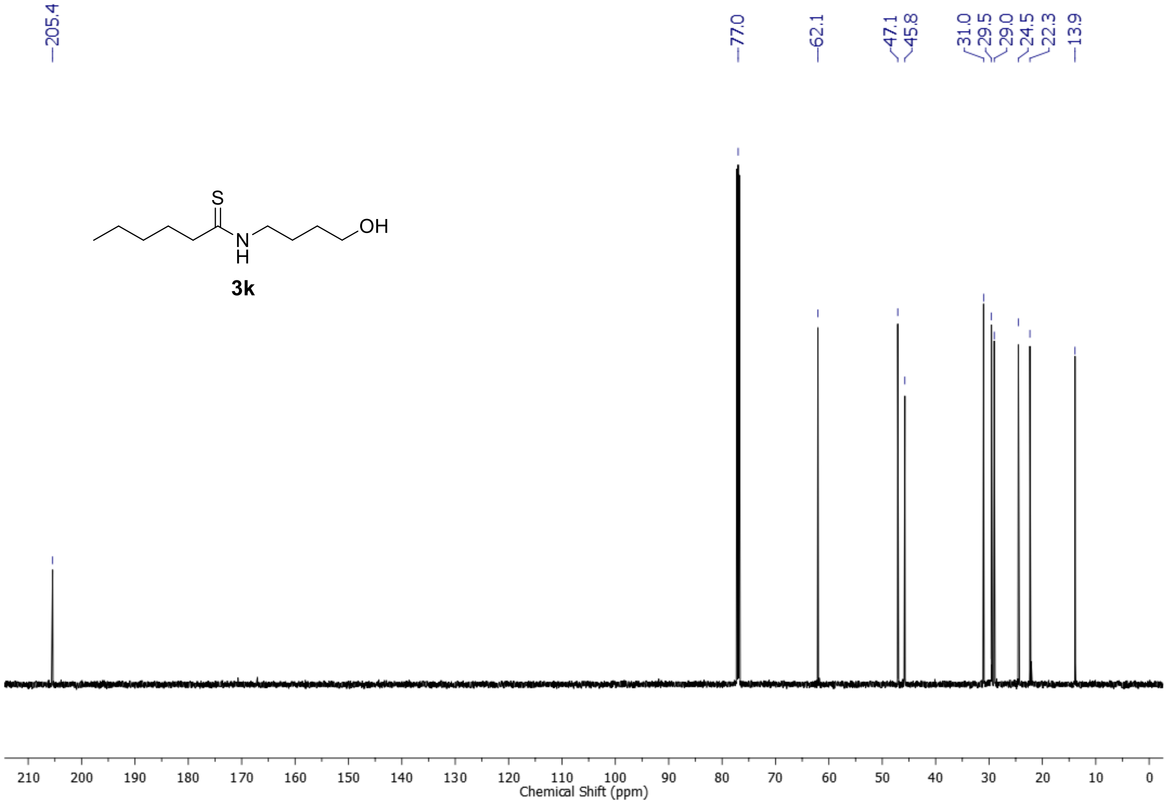

^{1}H NMR (600 MHz, $CDCl_3$) spectrum of compound **3l**

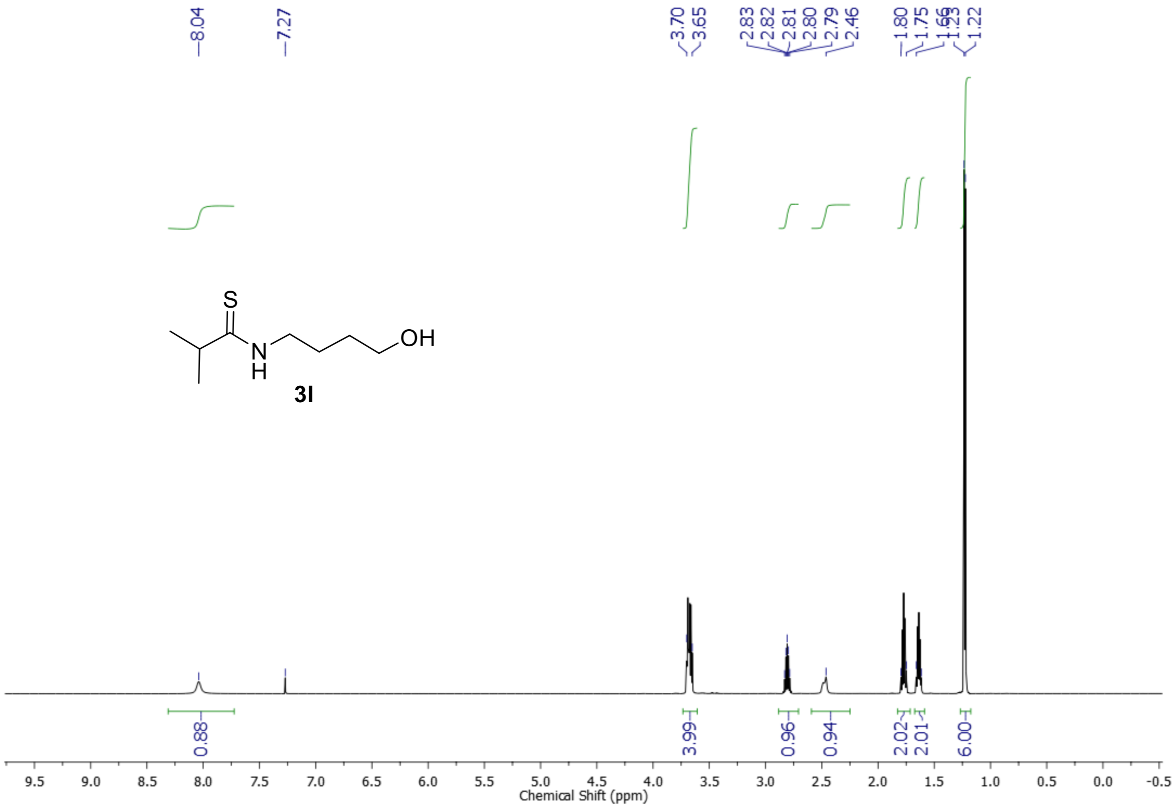

^{13}C NMR (151 MHz, $CDCl_3$) spectrum of compound **3l**

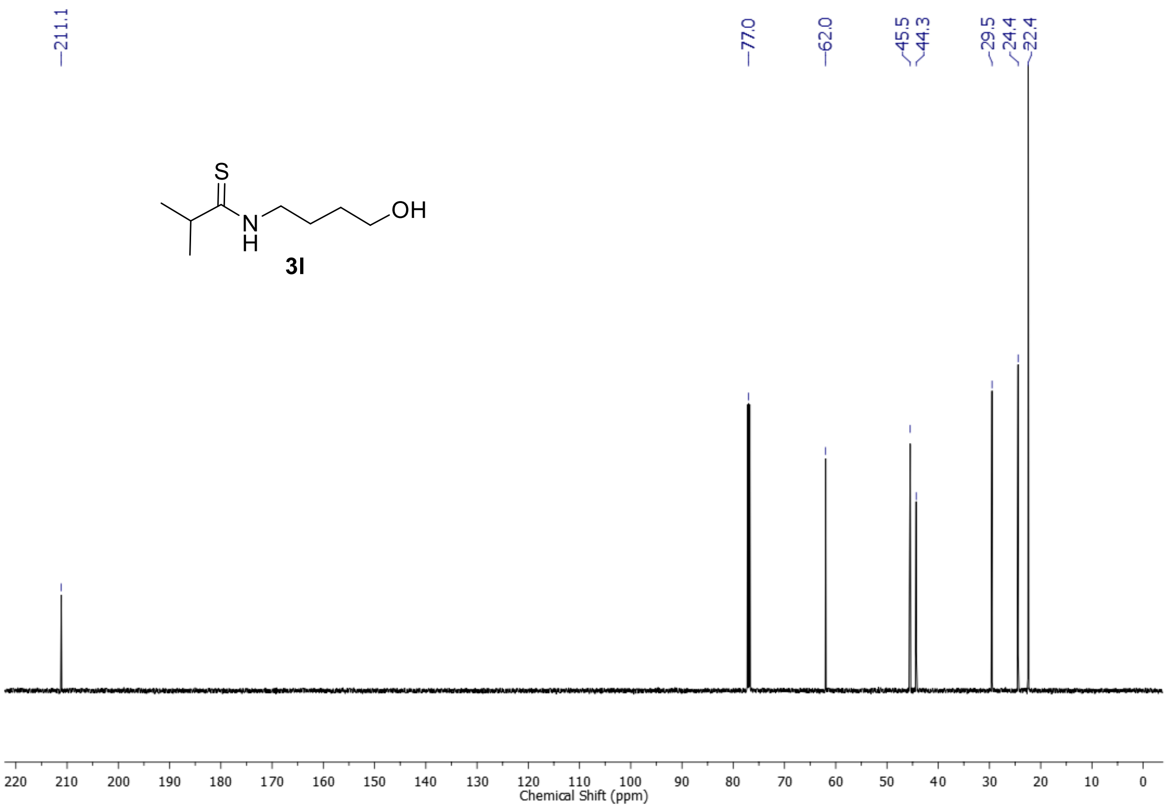

^{1}H NMR (600 MHz, $CDCl_3$) spectrum of compound **3m**

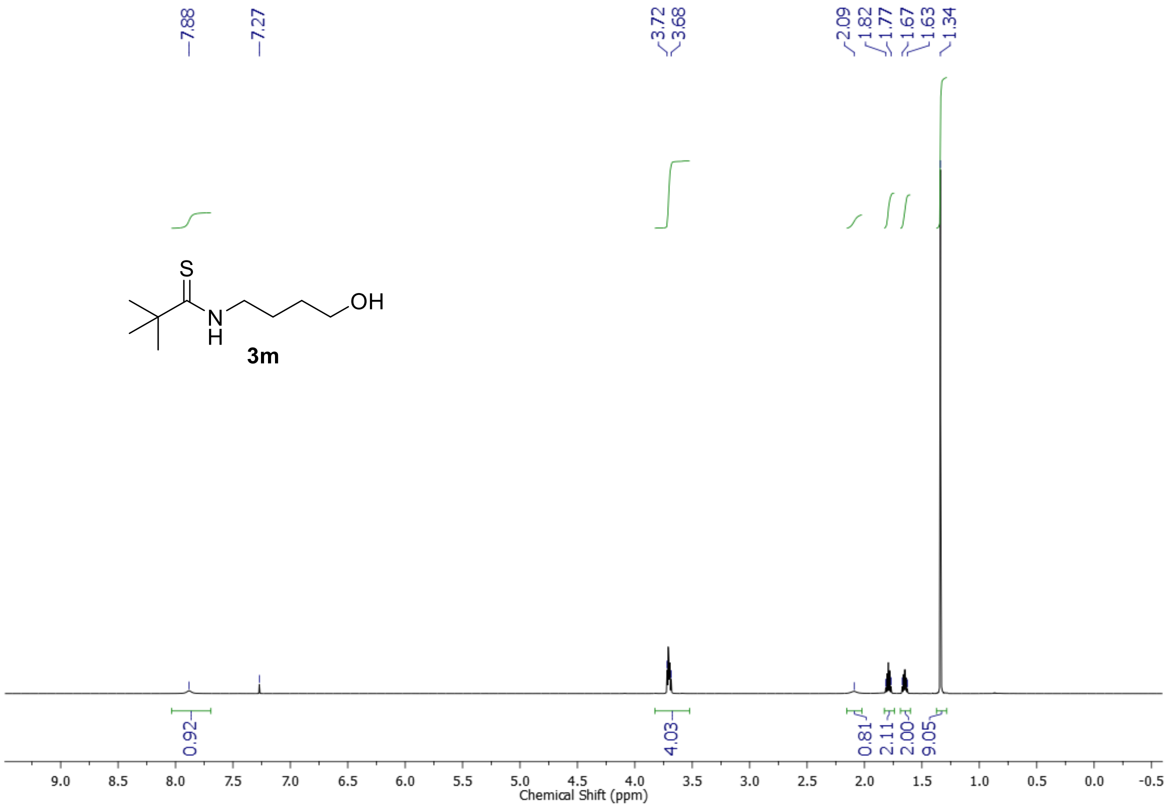

^{13}C NMR (151 MHz, $CDCl_3$) spectrum of compound **3m**

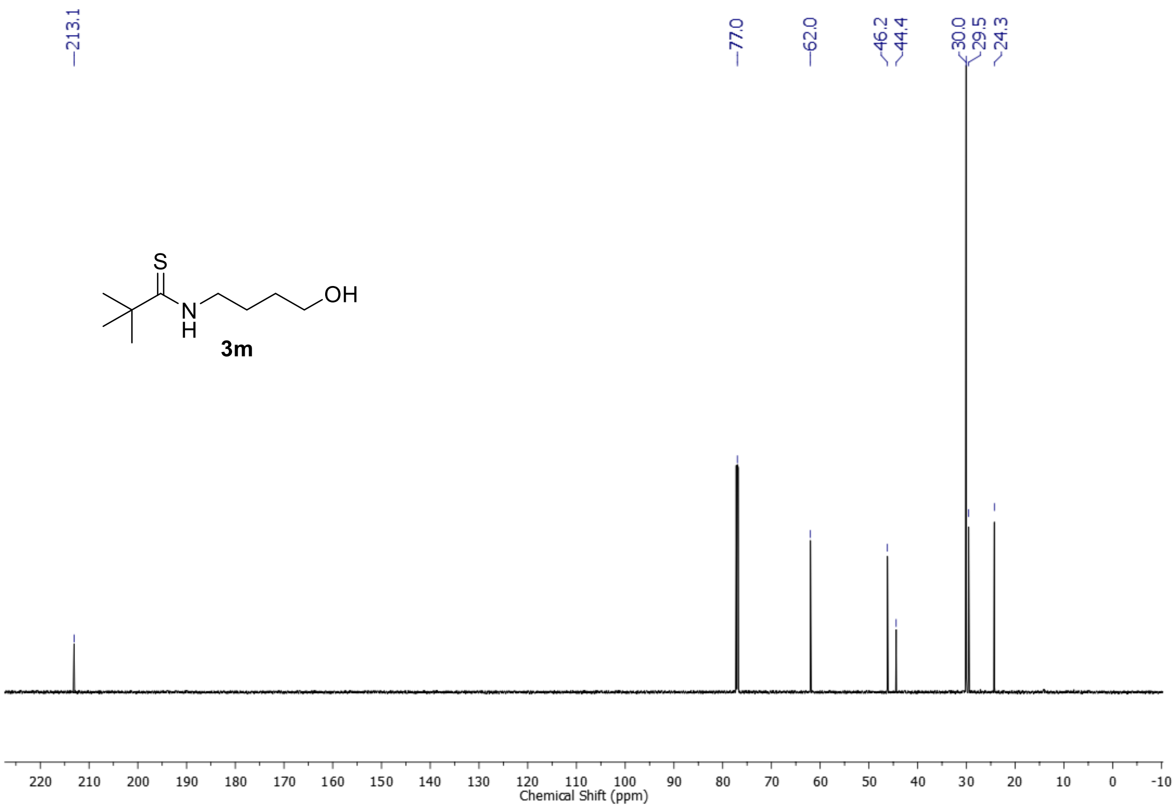

^{1}H NMR (600 MHz, $CDCl_3$) spectrum of compound **4a**

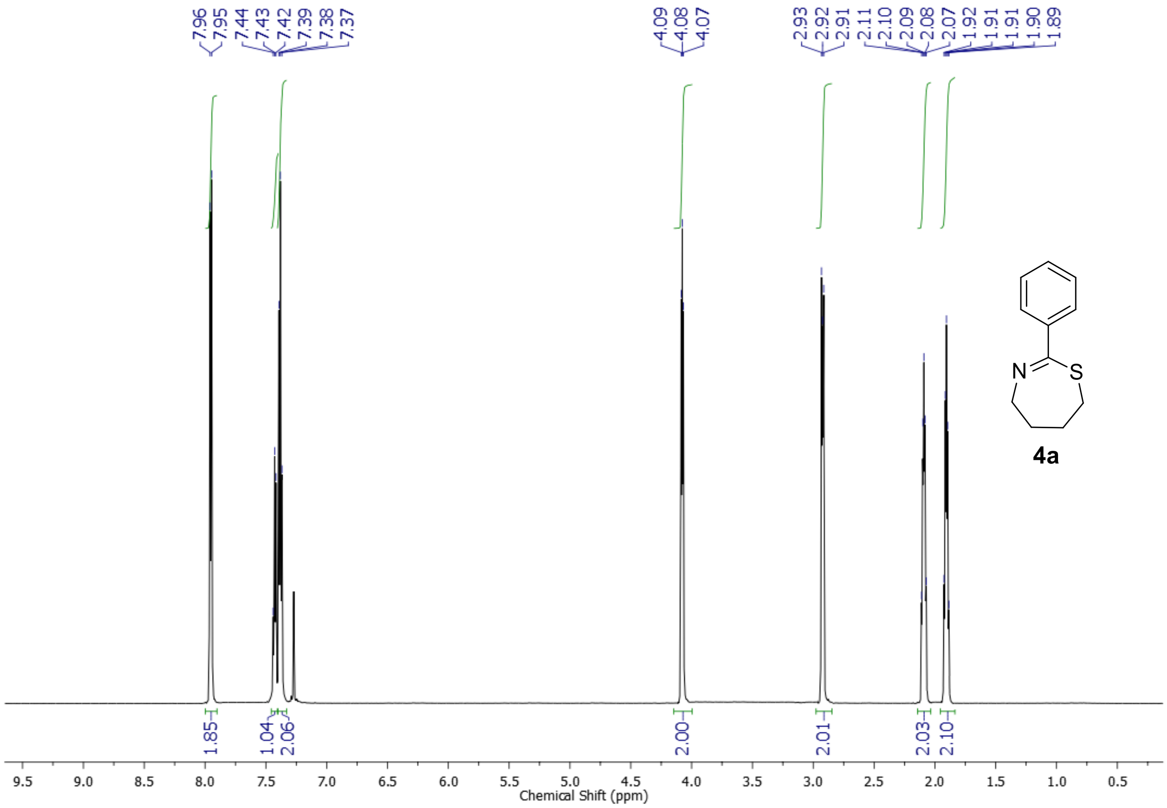

^{13}C NMR (151 MHz, $CDCl_3$) spectrum of compound **4a**

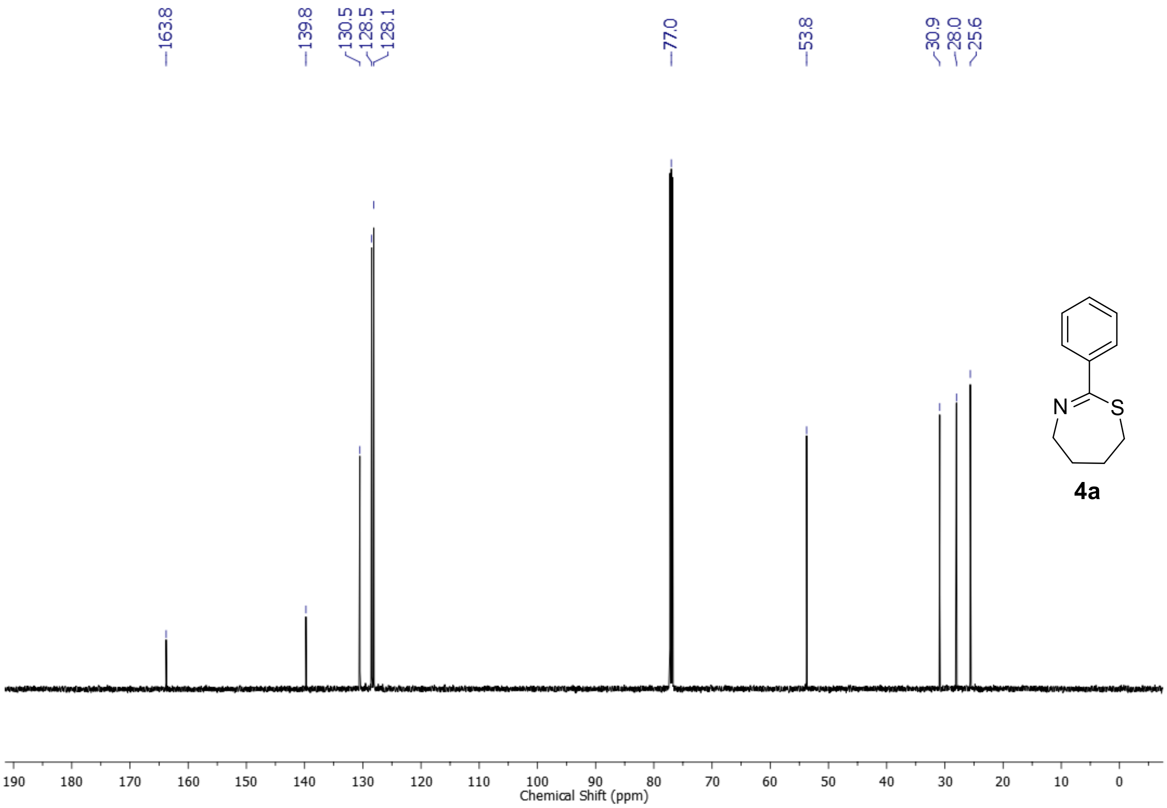

NOESY spectrum of compound **4a**

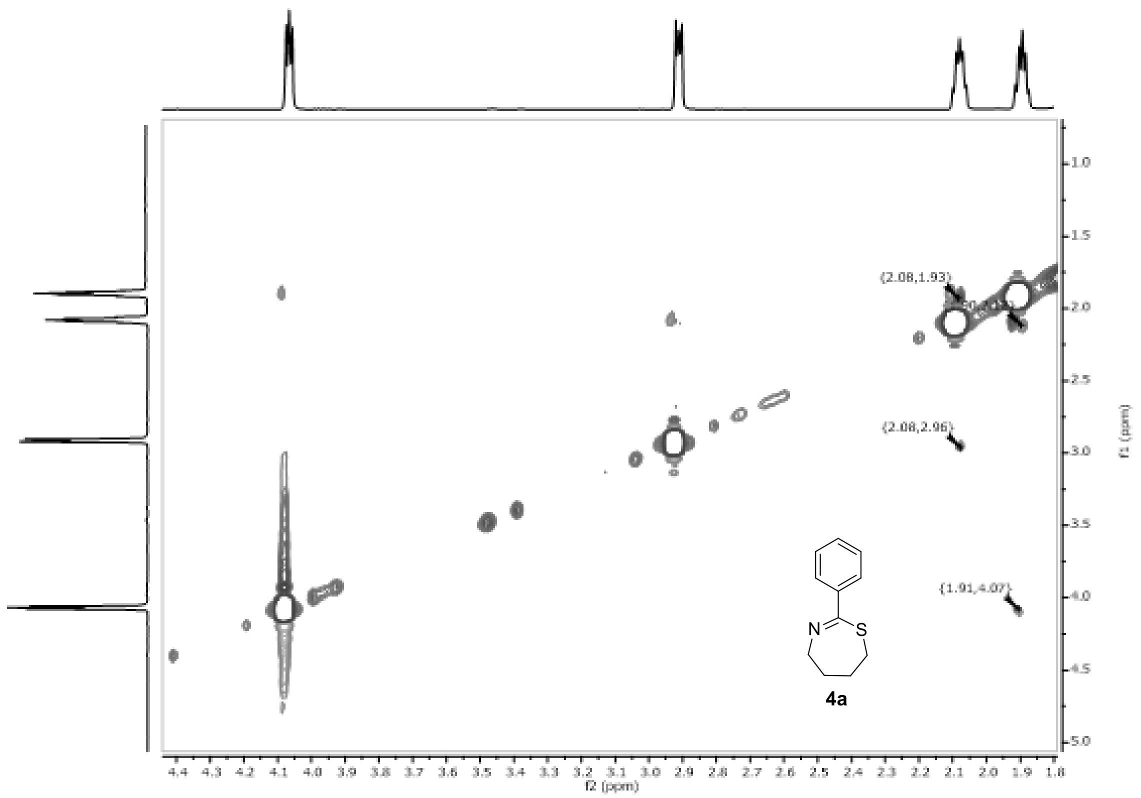

HSQC spectrum of compound **4a**

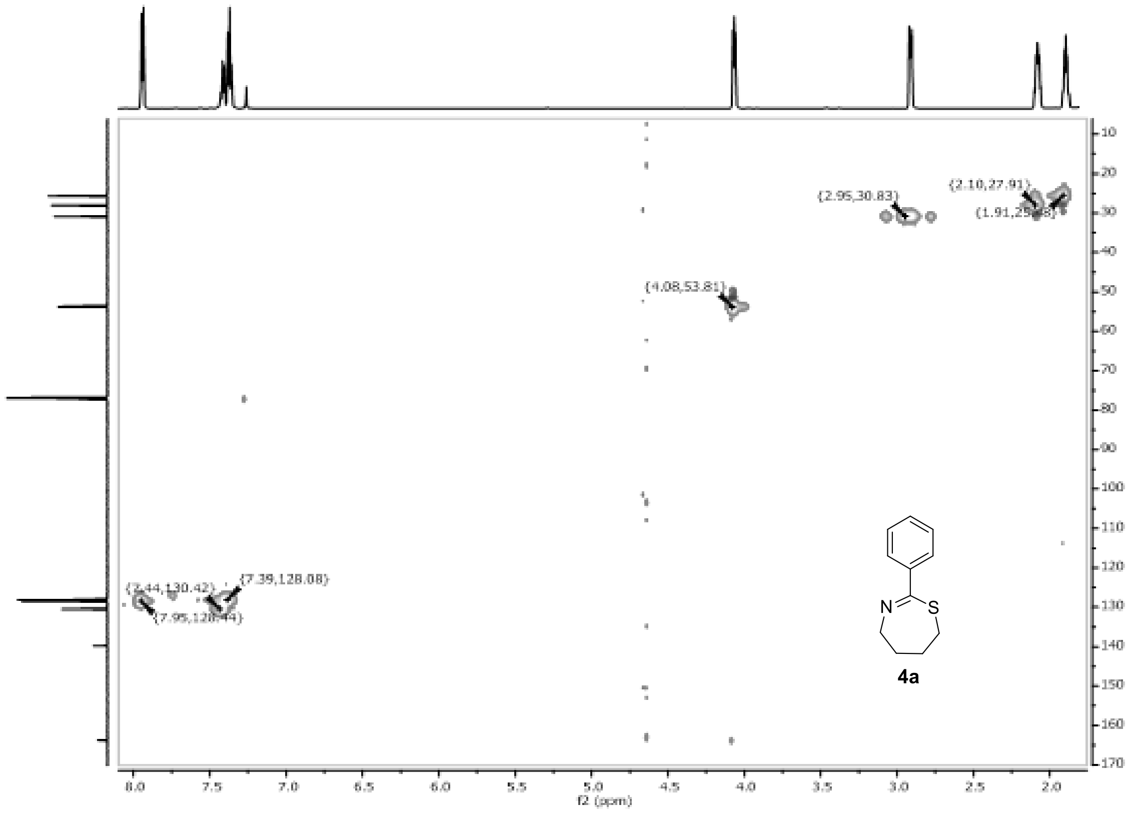

HMBC spectrum of compound **4a**

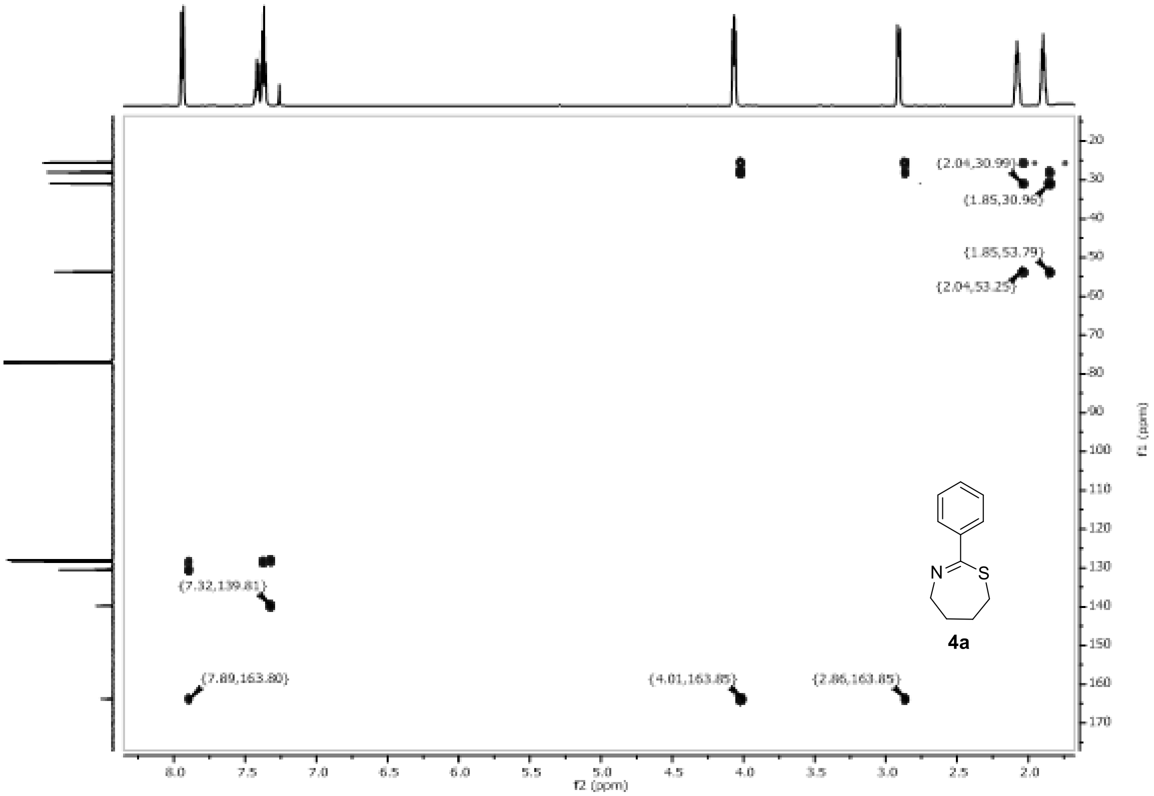

^{1}H NMR (500 MHz, $CDCl_3$) spectrum of compound **4b**

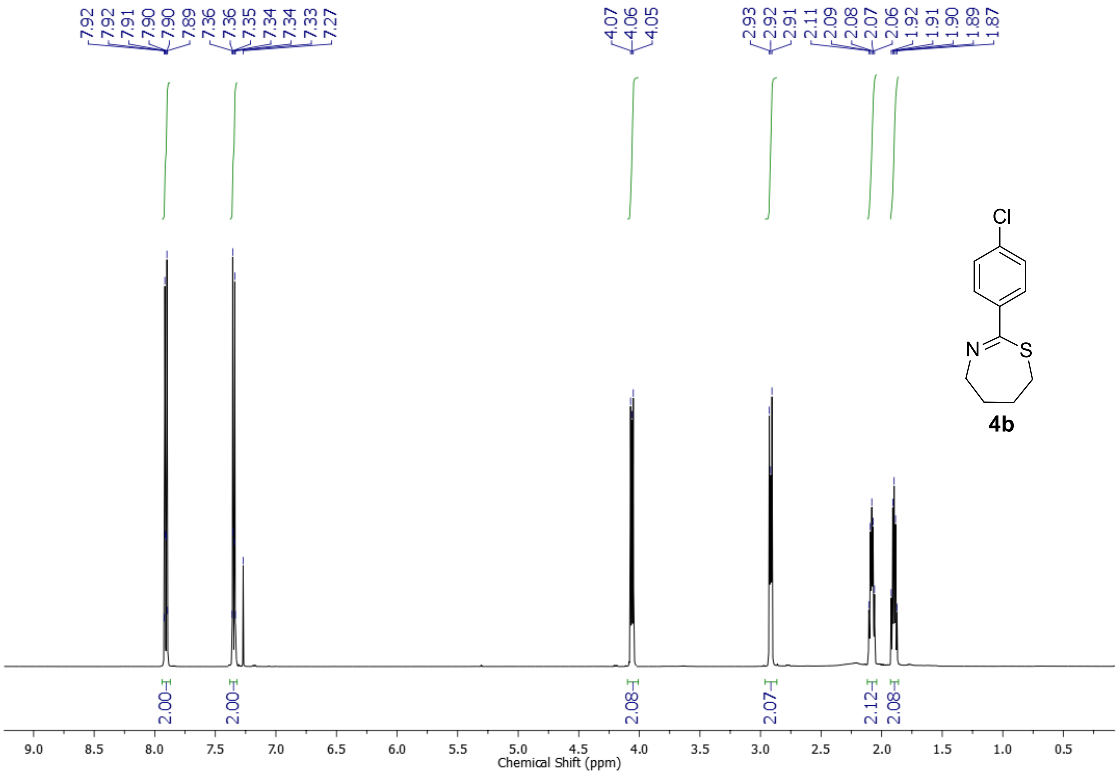

^{13}C NMR (125 MHz, $CDCl_3$) spectrum of compound **4b**

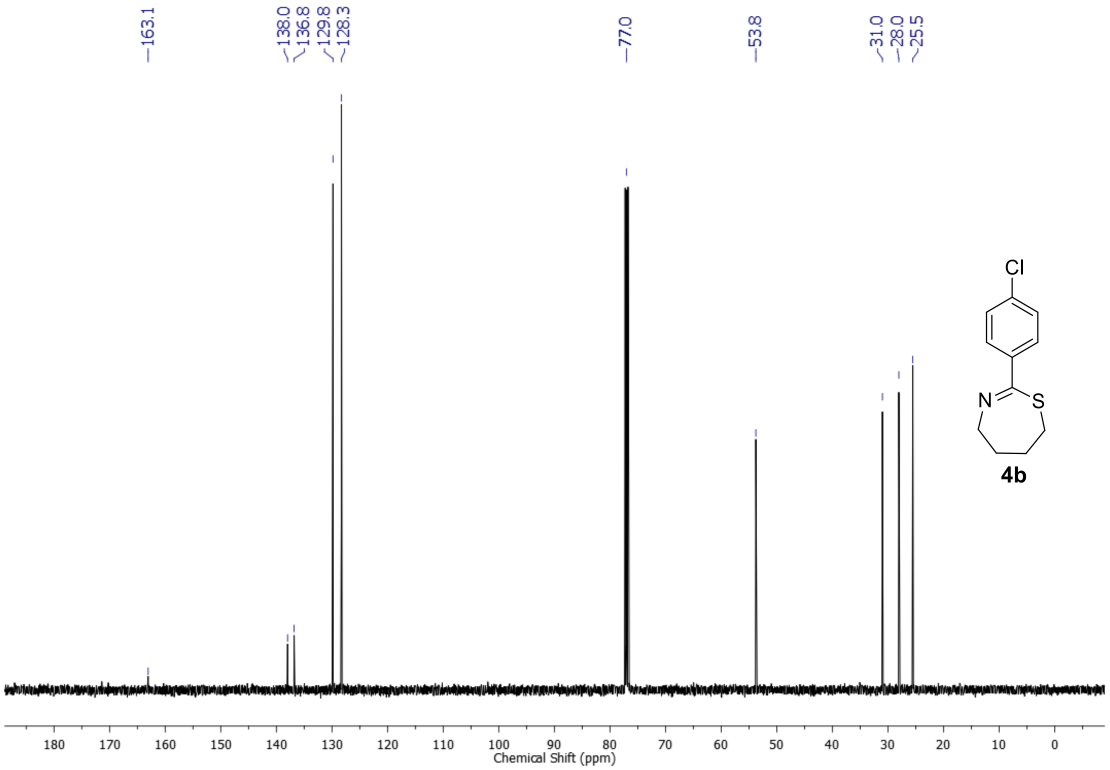

^{1}H NMR (300 MHz, $CDCl_3$) spectrum of compound **4c**

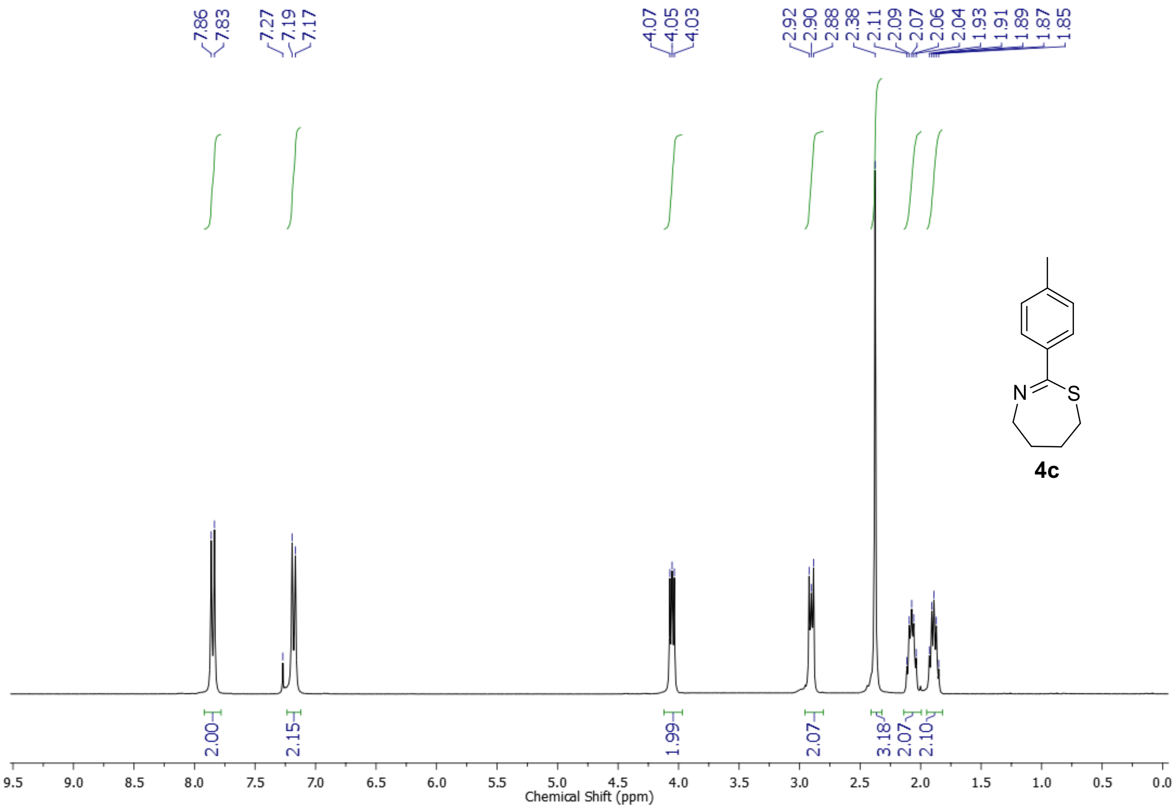

^{13}C NMR (75 MHz, $CDCl_3$) spectrum of compound **4c**

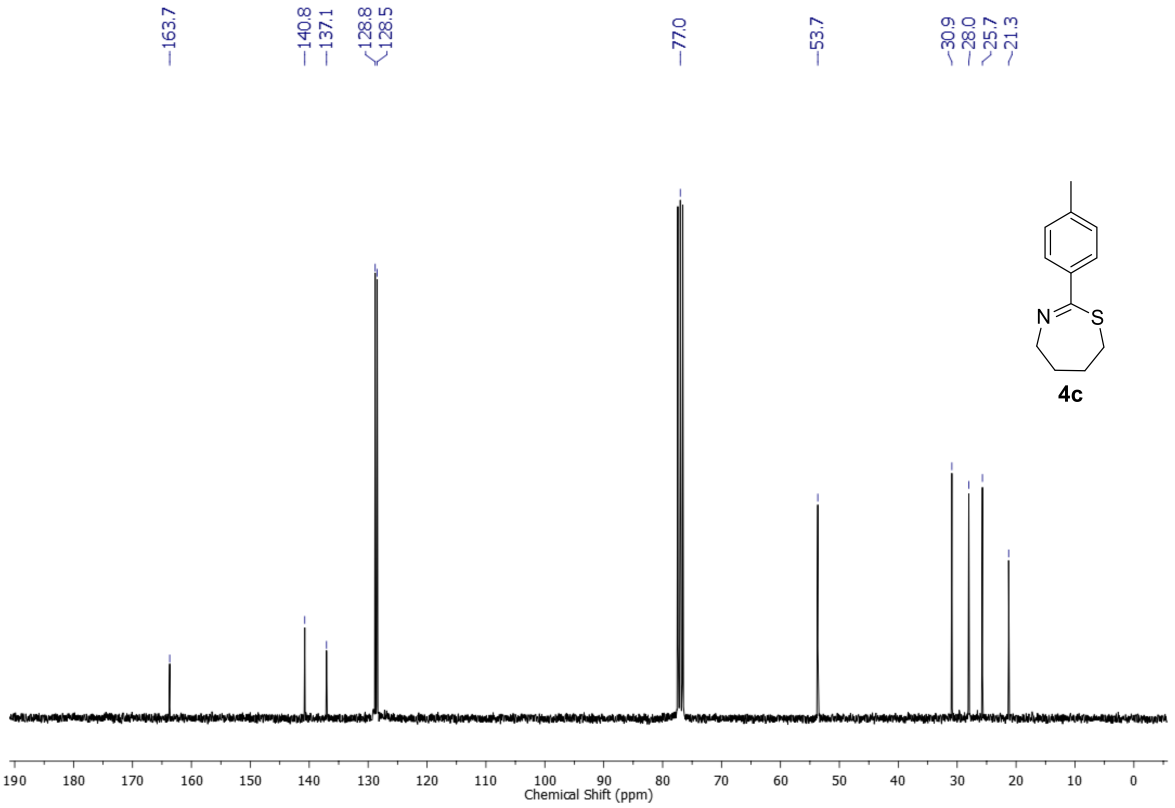

^{1}H NMR (500 MHz, $CDCl_3$) spectrum of compound **4d**

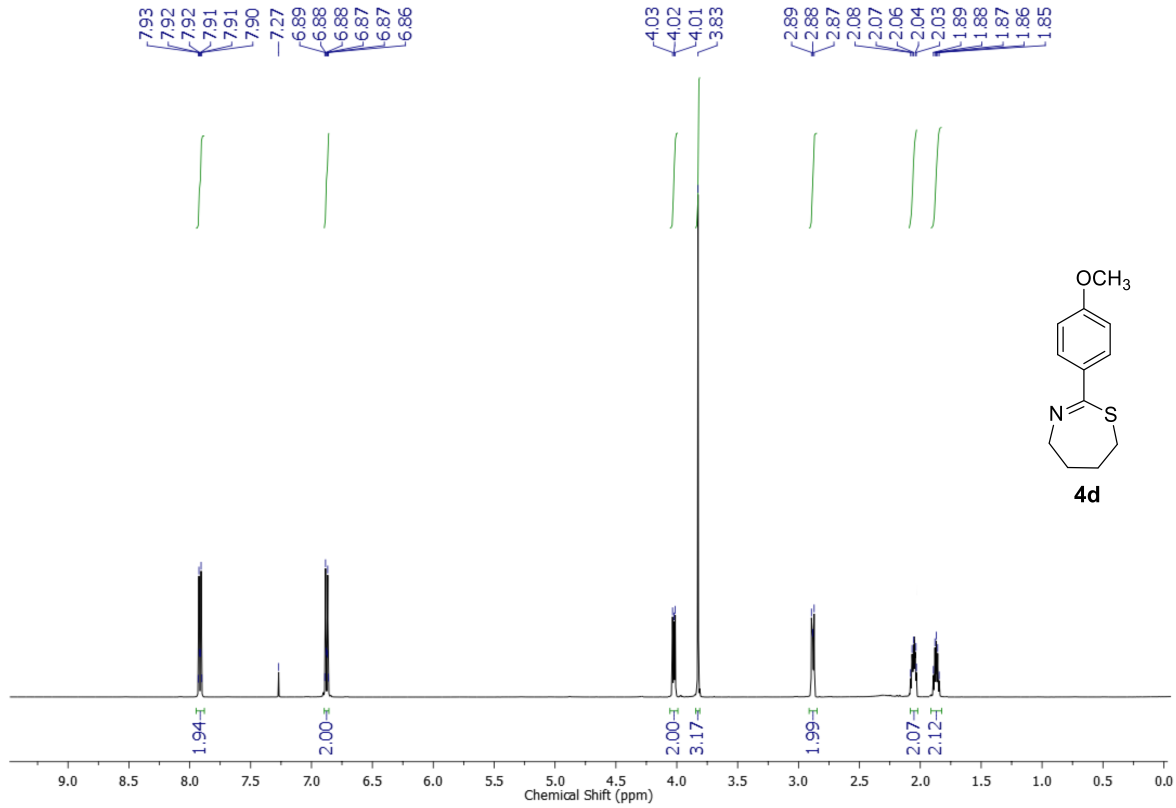

^{13}C NMR (125 MHz, $CDCl_3$) spectrum of compound **4d**

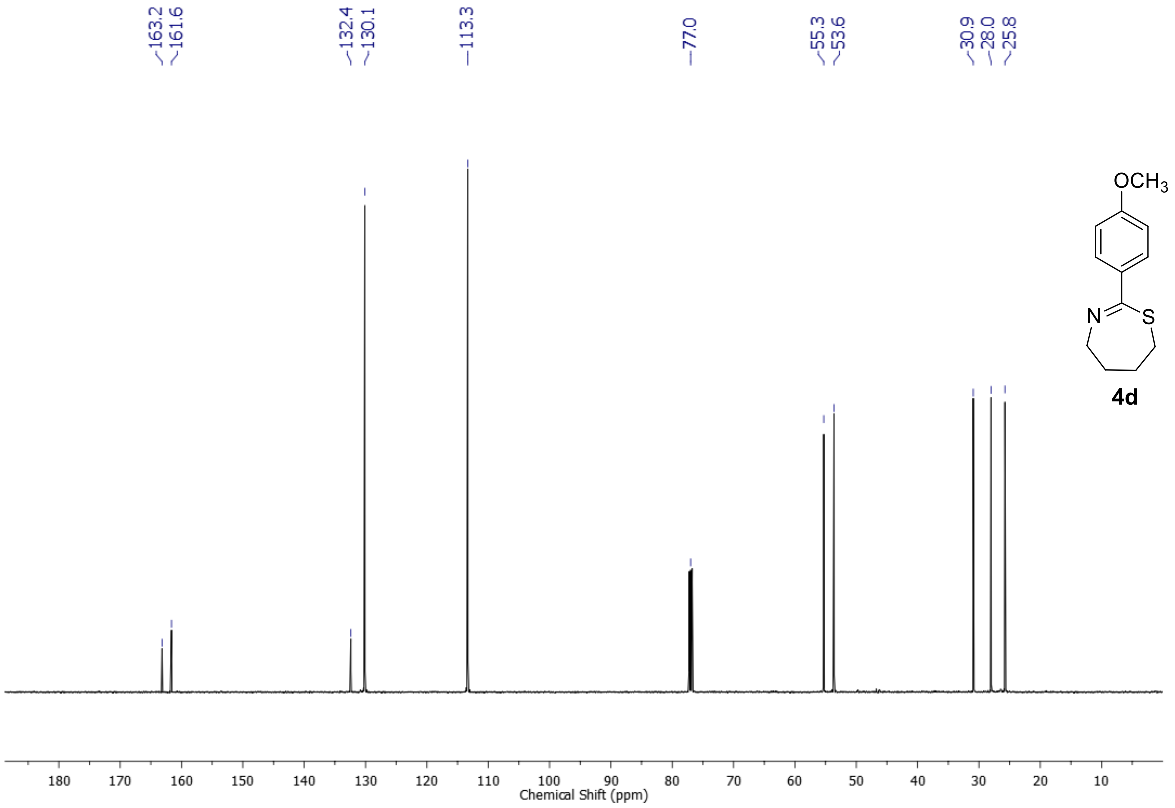

^{1}H NMR (500 MHz, $CDCl_3$) spectrum of compound **4e**

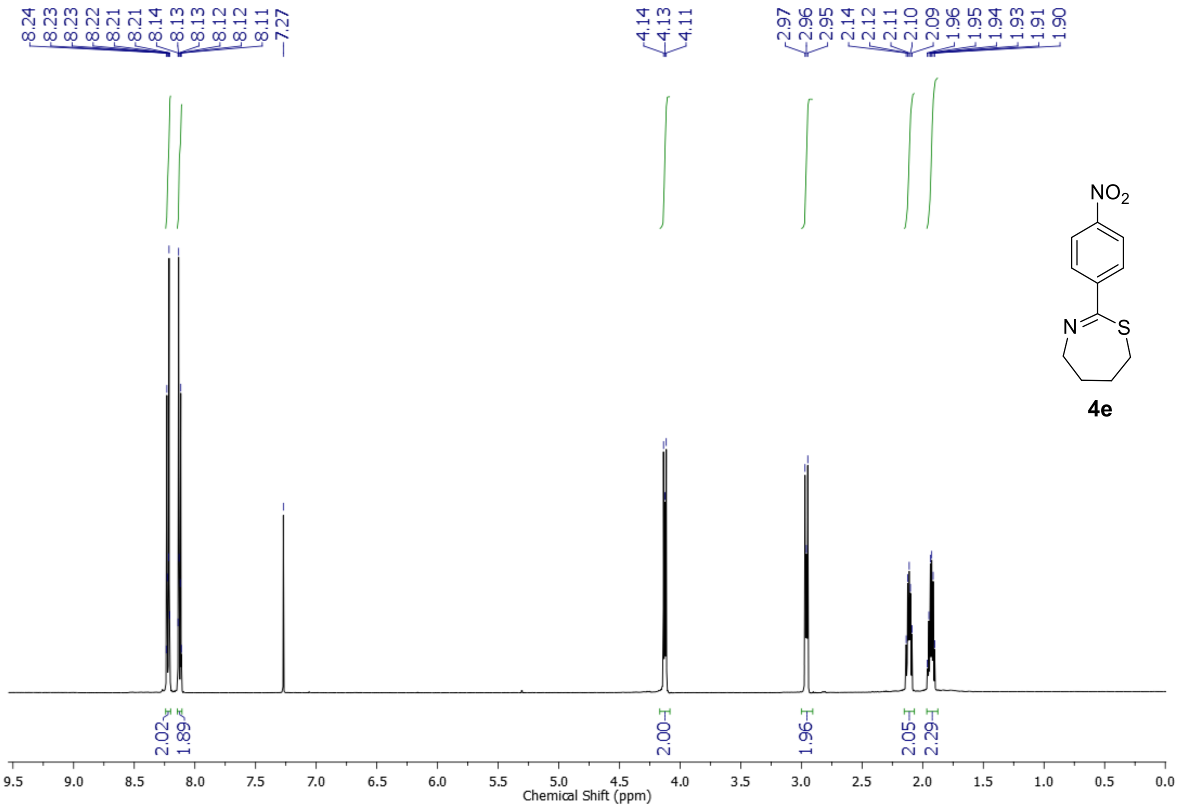

^{13}C NMR (125 MHz, $CDCl_3$) spectrum of compound **4e**

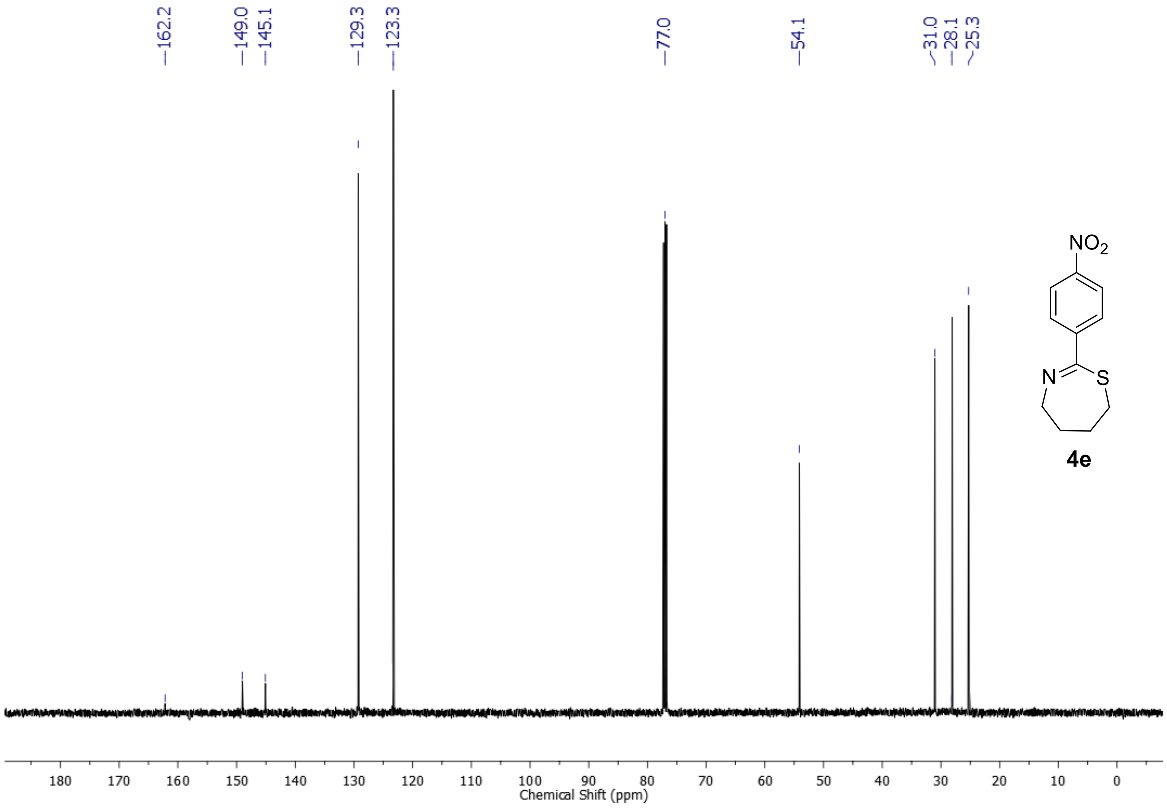

^{1}H NMR (500 MHz, $CDCl_3$) spectrum of compound **4f**

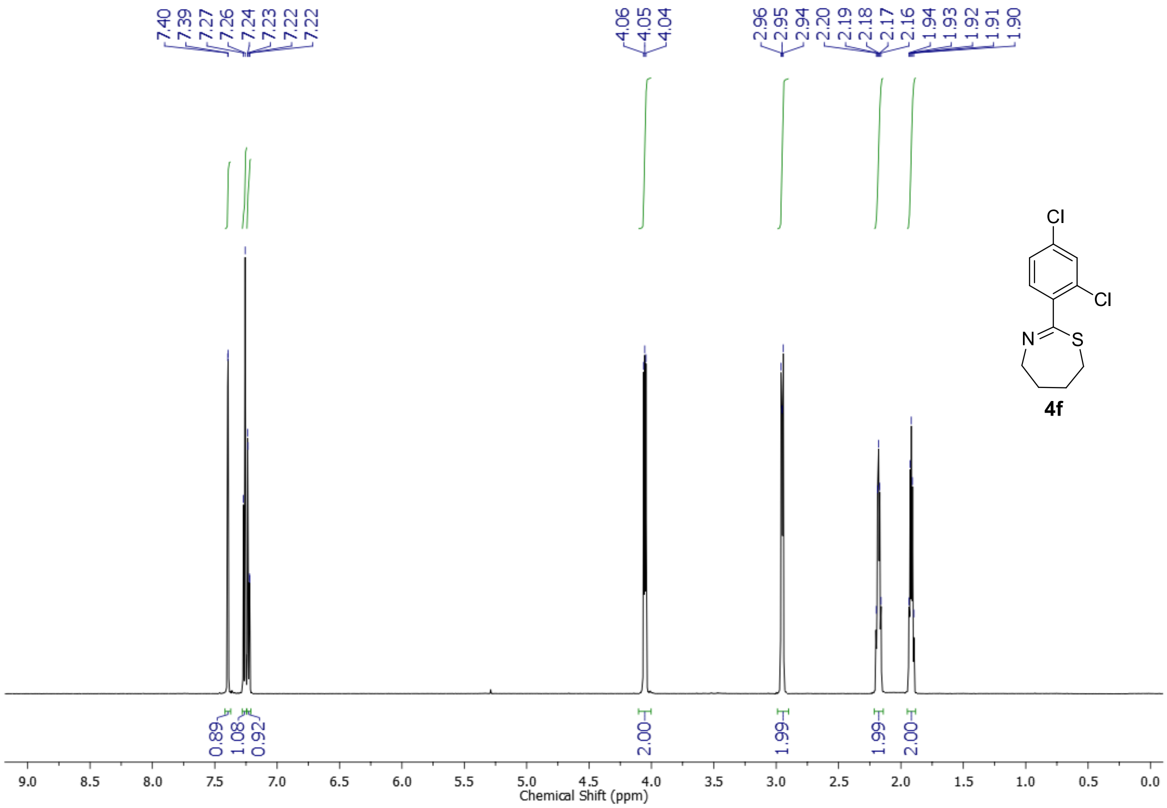

^{13}C NMR (125 MHz, $CDCl_3$) spectrum of compound **4f**

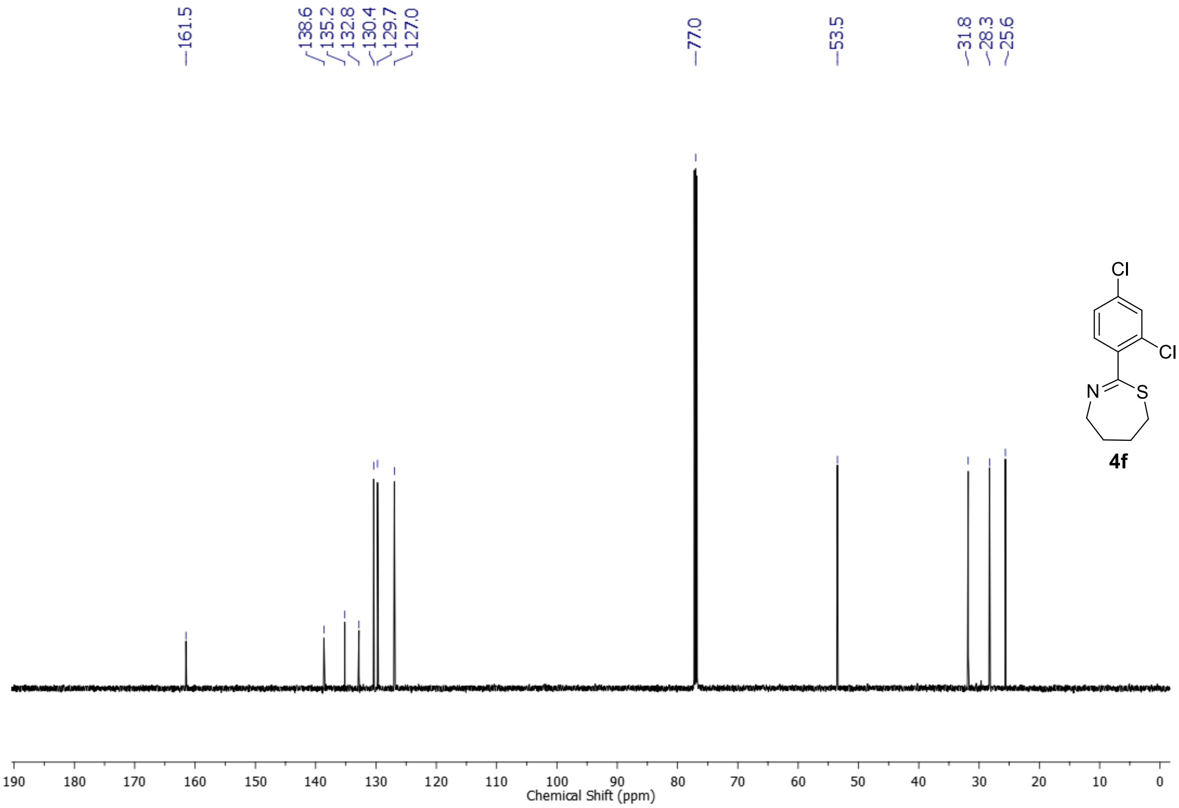

^{1}H NMR (500 MHz, $CDCl_3$) spectrum of compound **4g**

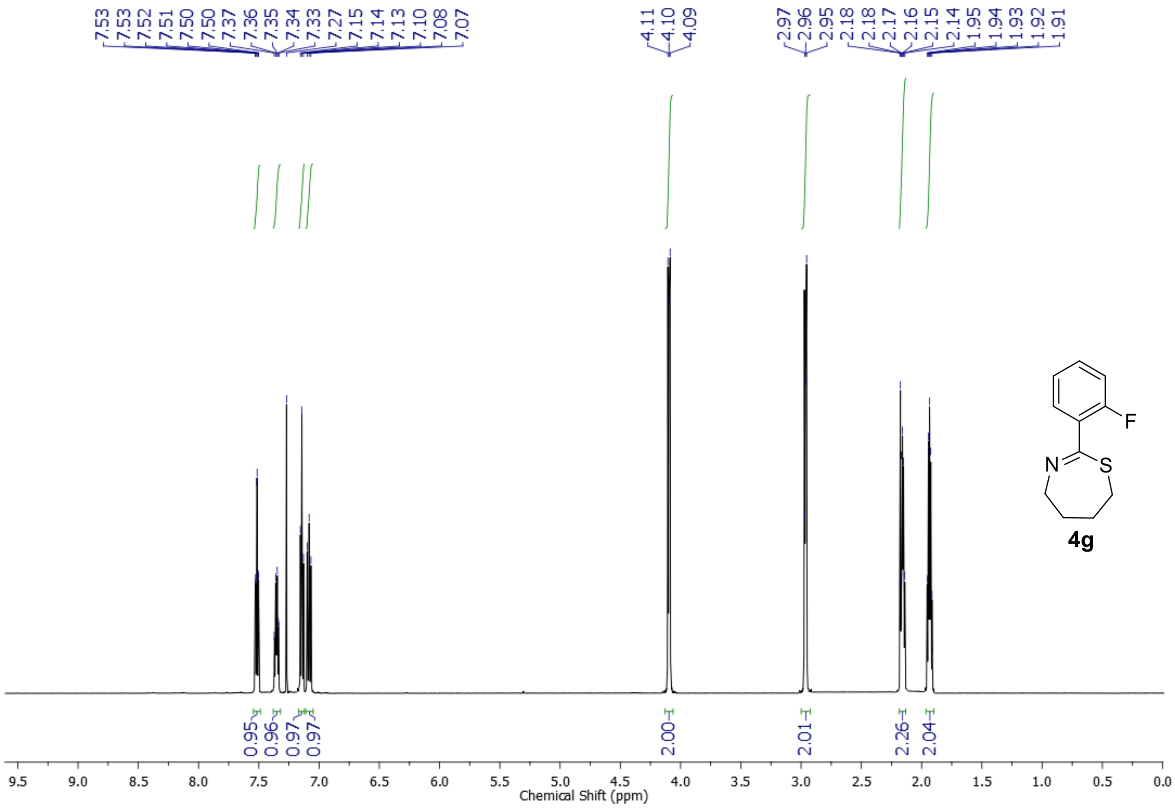

^{13}C NMR (125 MHz, $CDCl_3$) spectrum of compound **4g**

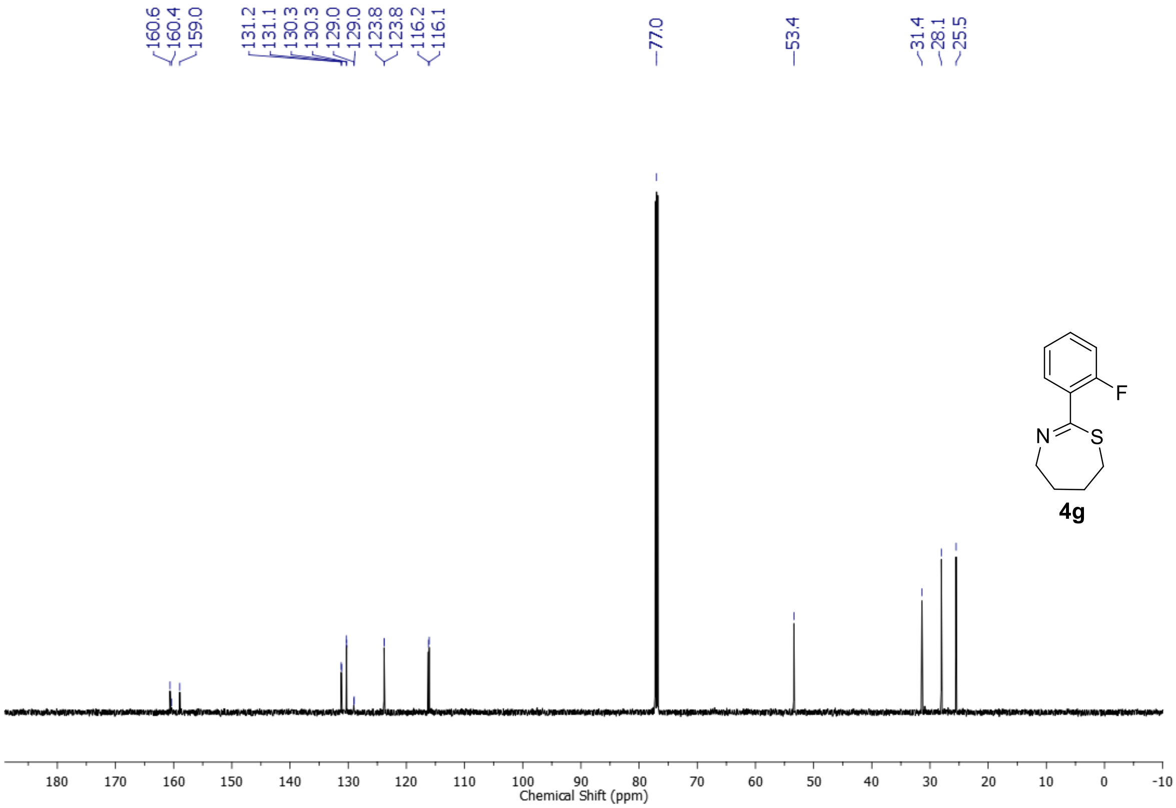

^{1}H NMR (500 MHz, $CDCl_3$) spectrum of compound **4h**

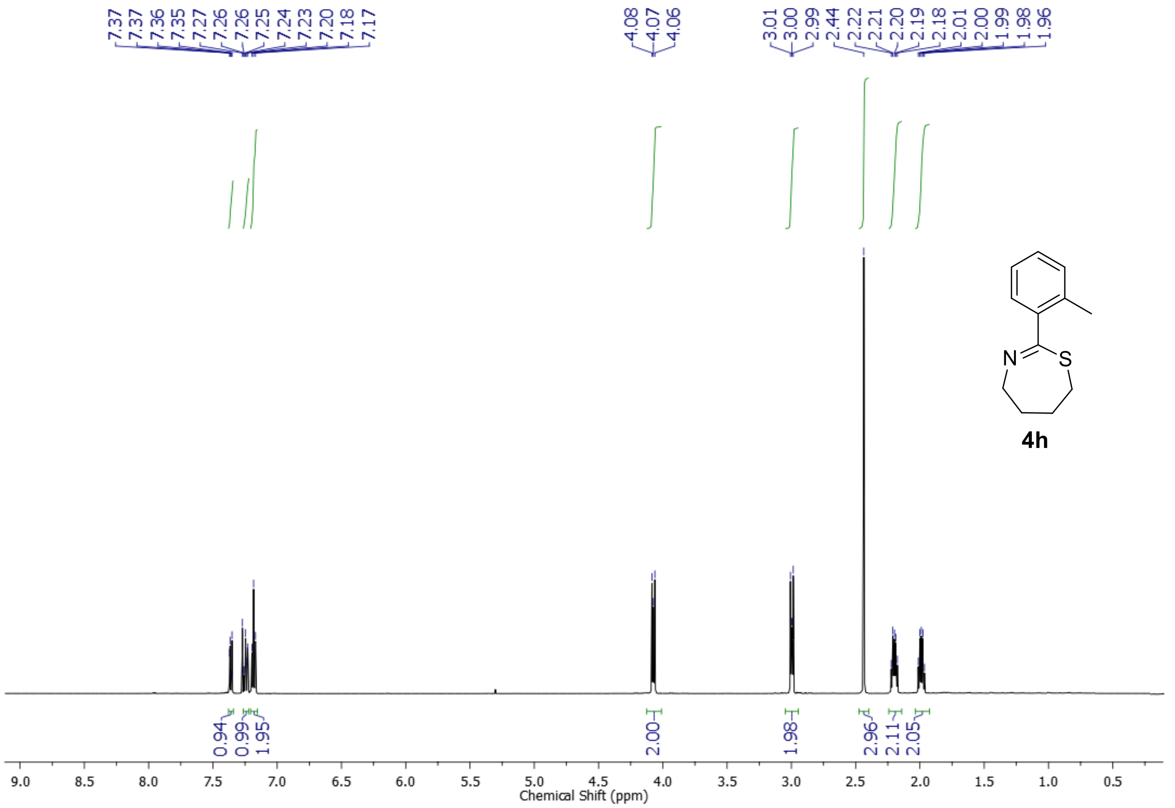

^{13}C NMR (125 MHz, $CDCl_3$) spectrum of compound **4h**

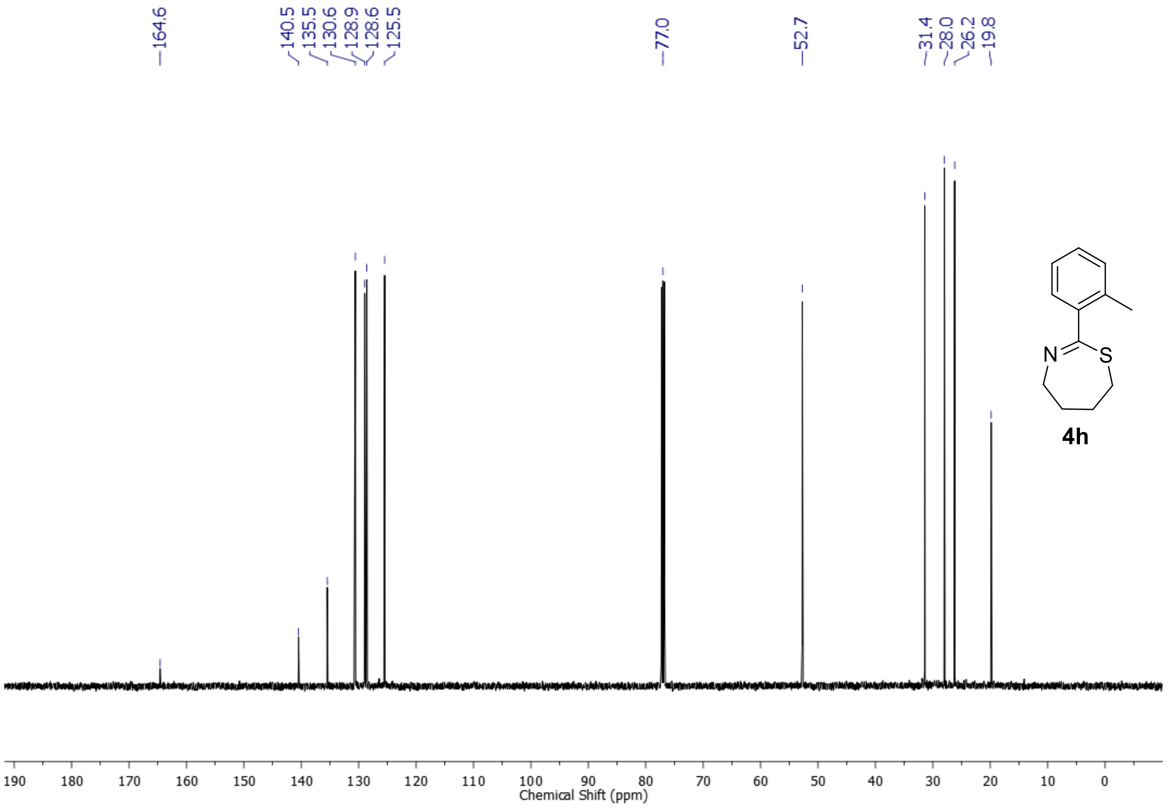

^{1}H NMR (600 MHz, $CDCl_3$) spectrum of compound **4i**

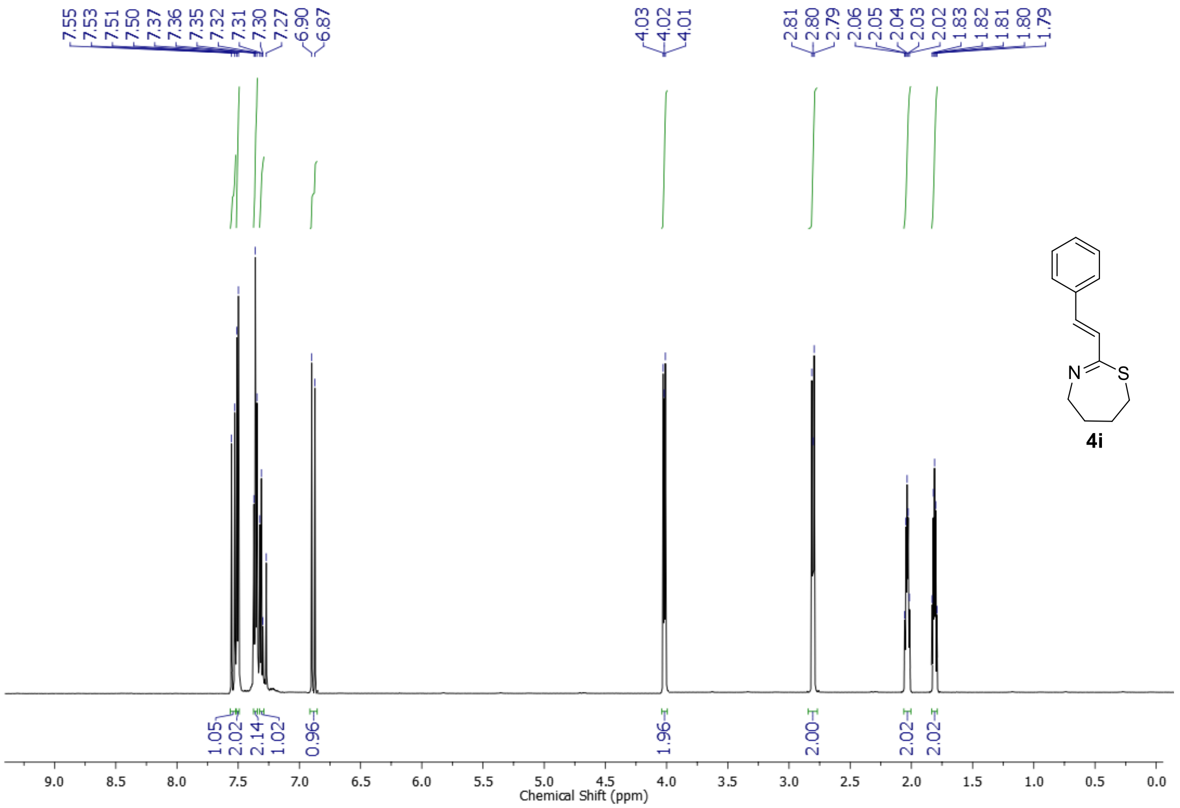

^{13}C NMR (151 MHz, $CDCl_3$) spectrum of compound **4i**

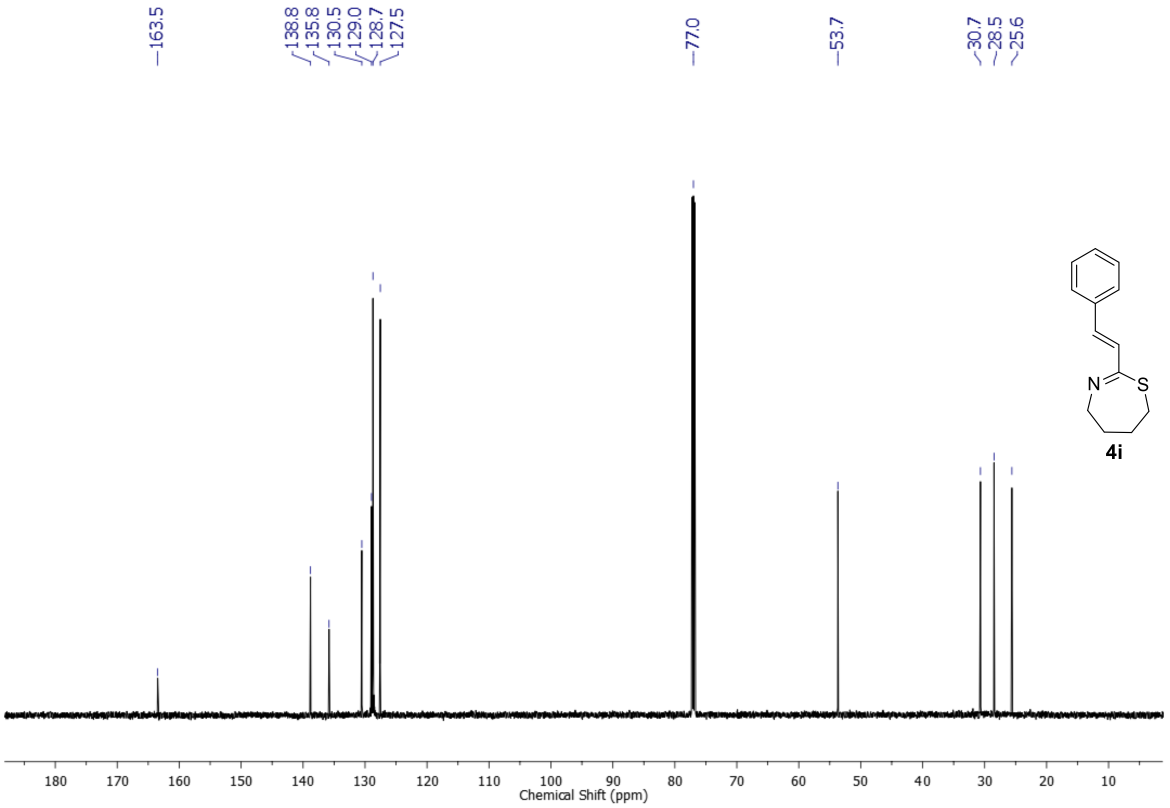

^{1}H NMR (600 MHz, $CDCl_3$) spectrum of compound **4j**

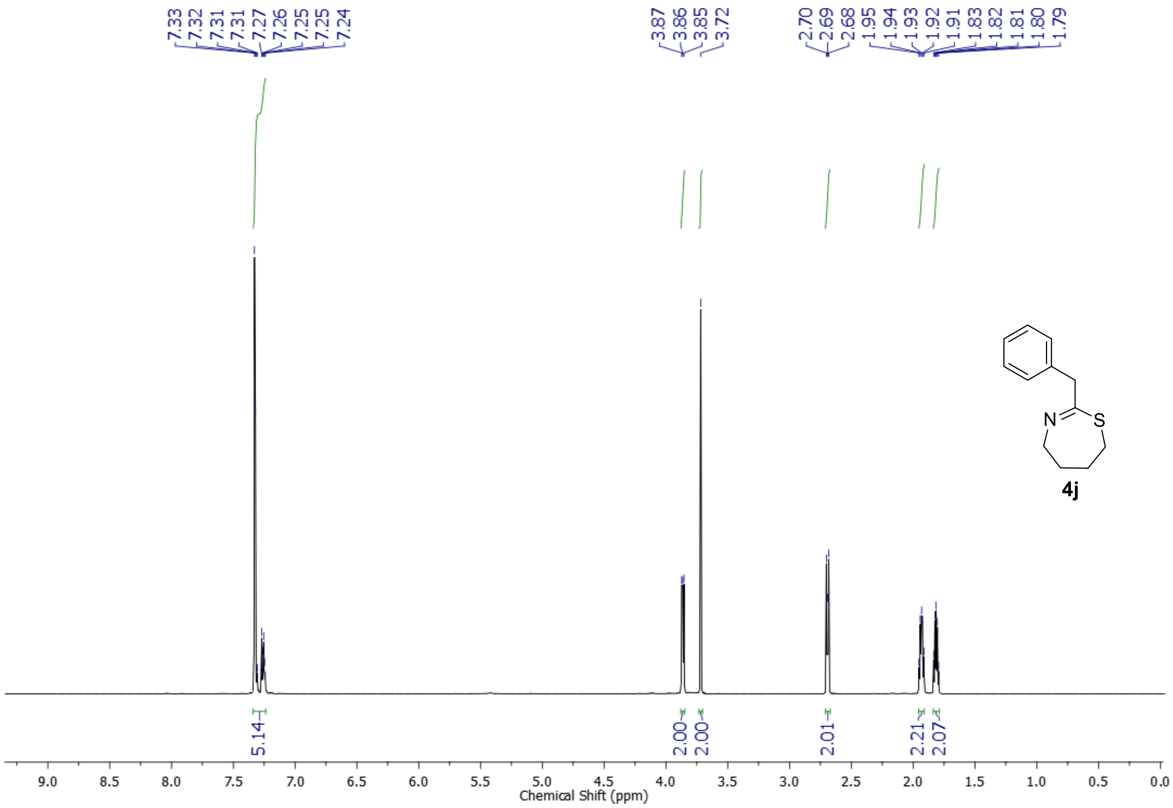

^{13}C NMR (151 MHz, $CDCl_3$) spectrum of compound **4j**

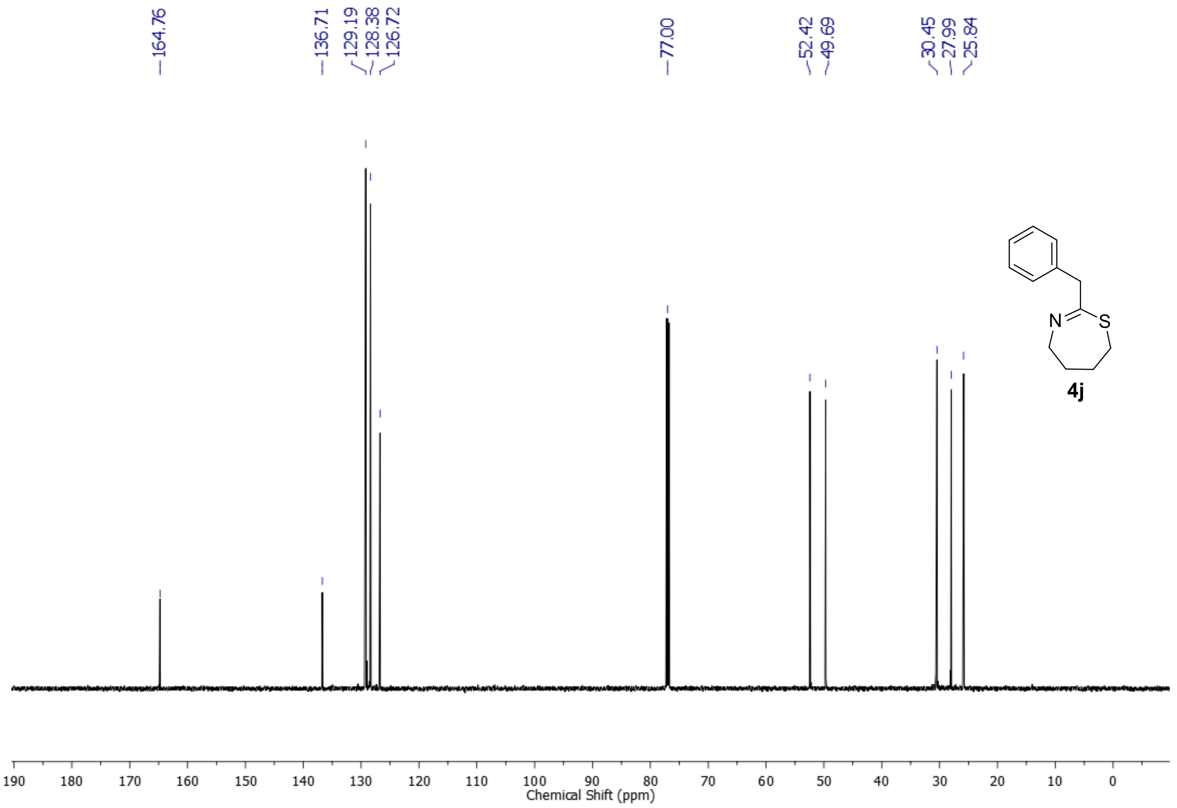

^{1}H NMR (600 MHz, $CDCl_3$) spectrum of compound **4k**

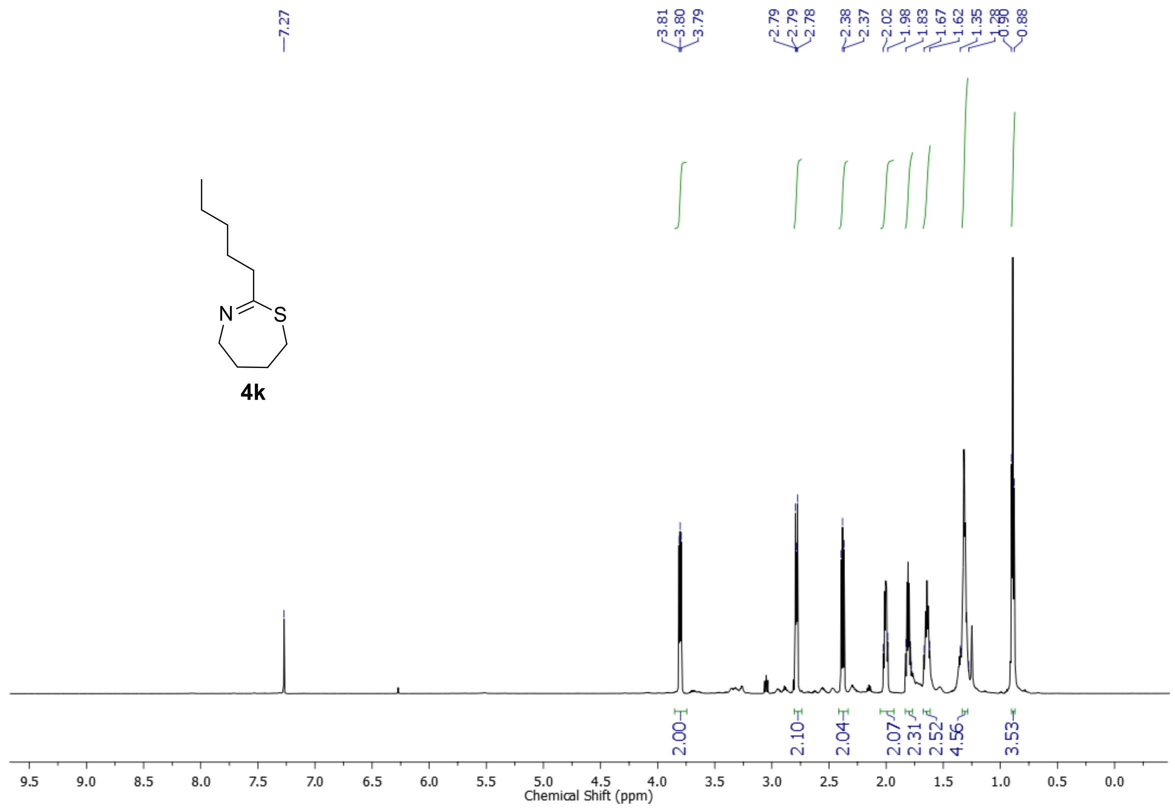

^{13}C NMR (151 MHz, $CDCl_3$) spectrum of compound **4k**

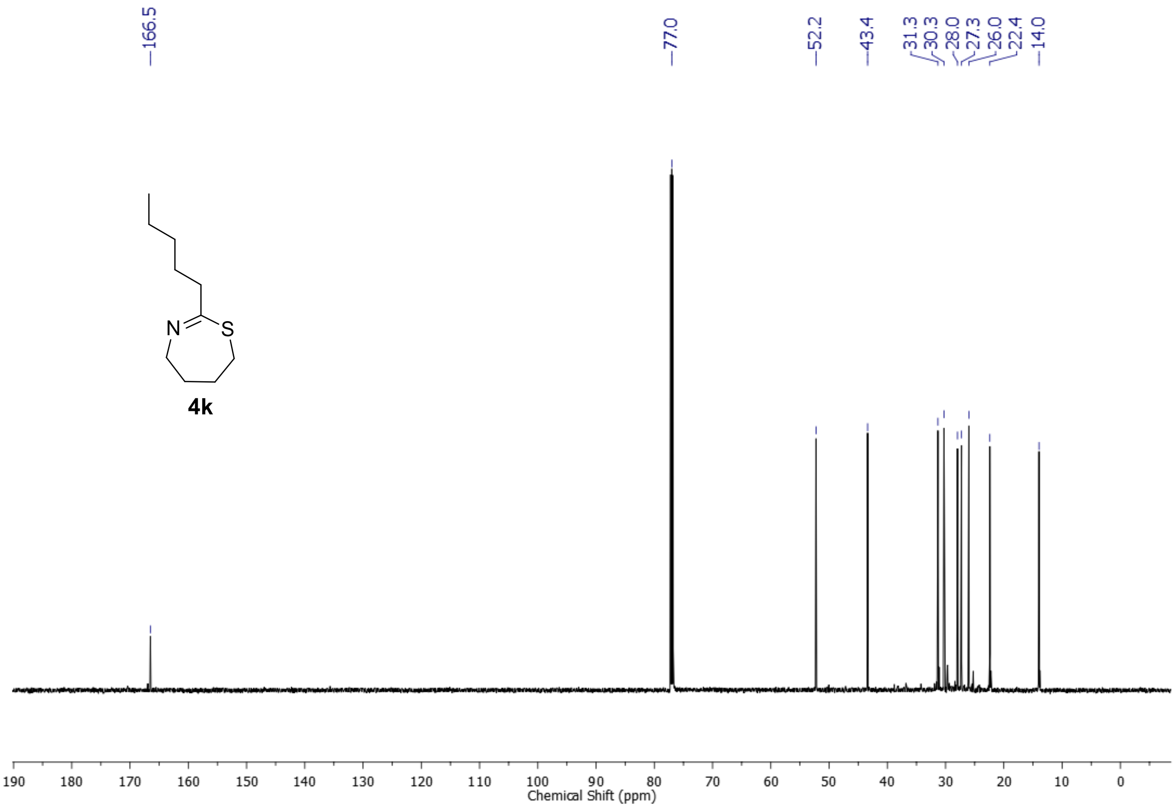

Compound **4k** underwent partial decomposition after purification.

^{1}H NMR (600 MHz, $CDCl_3$) spectrum of compound **4l**

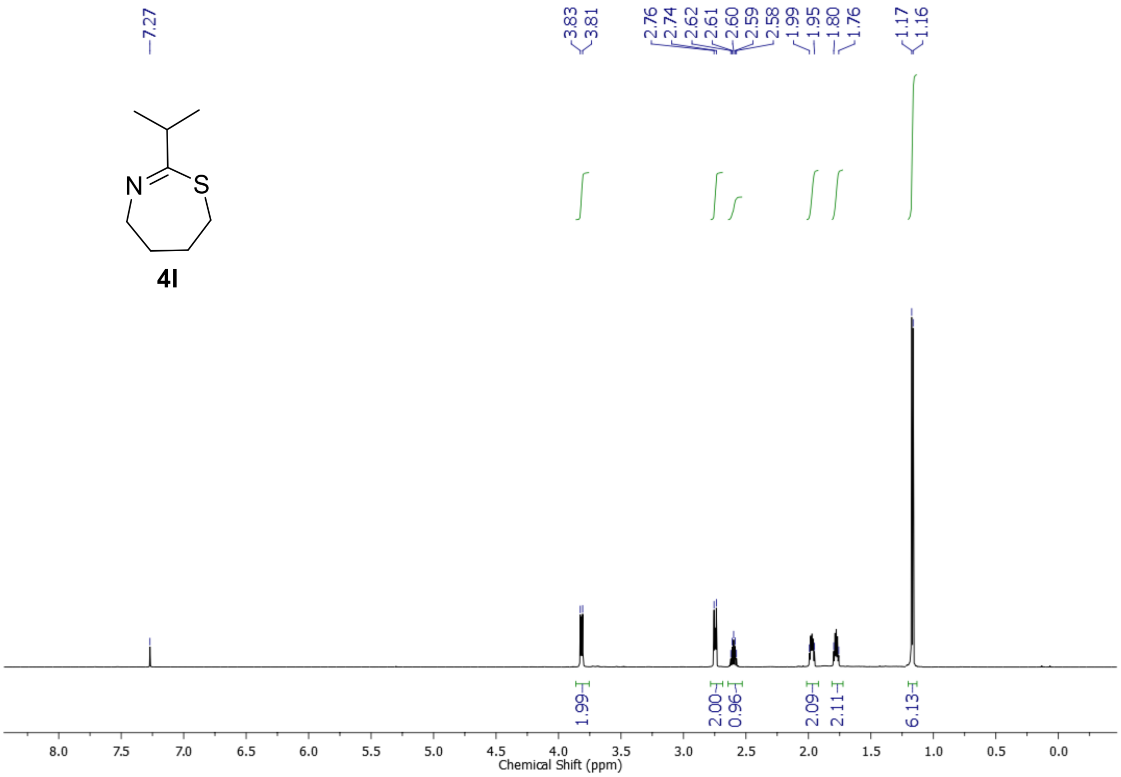

^{13}C NMR (151 MHz, $CDCl_3$) spectrum of compound **4l**

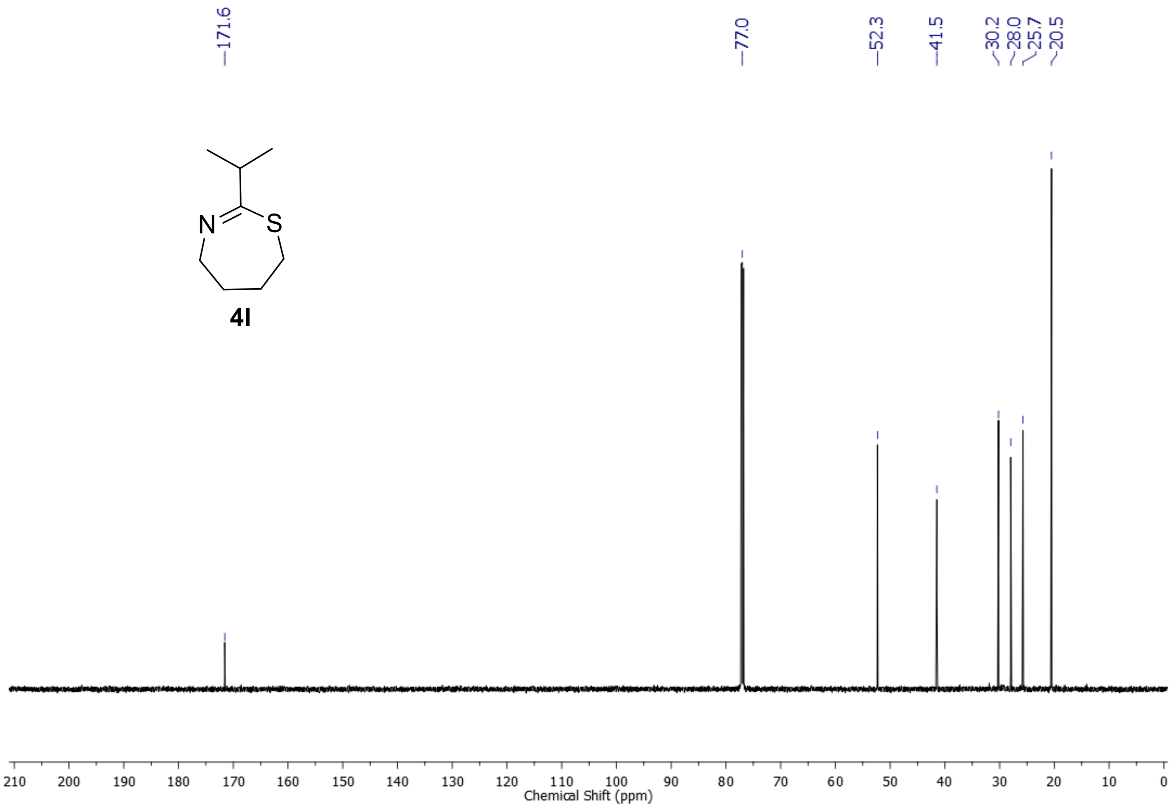

^{1}H NMR (600 MHz, $CDCl_3$) spectrum of compound **4m**

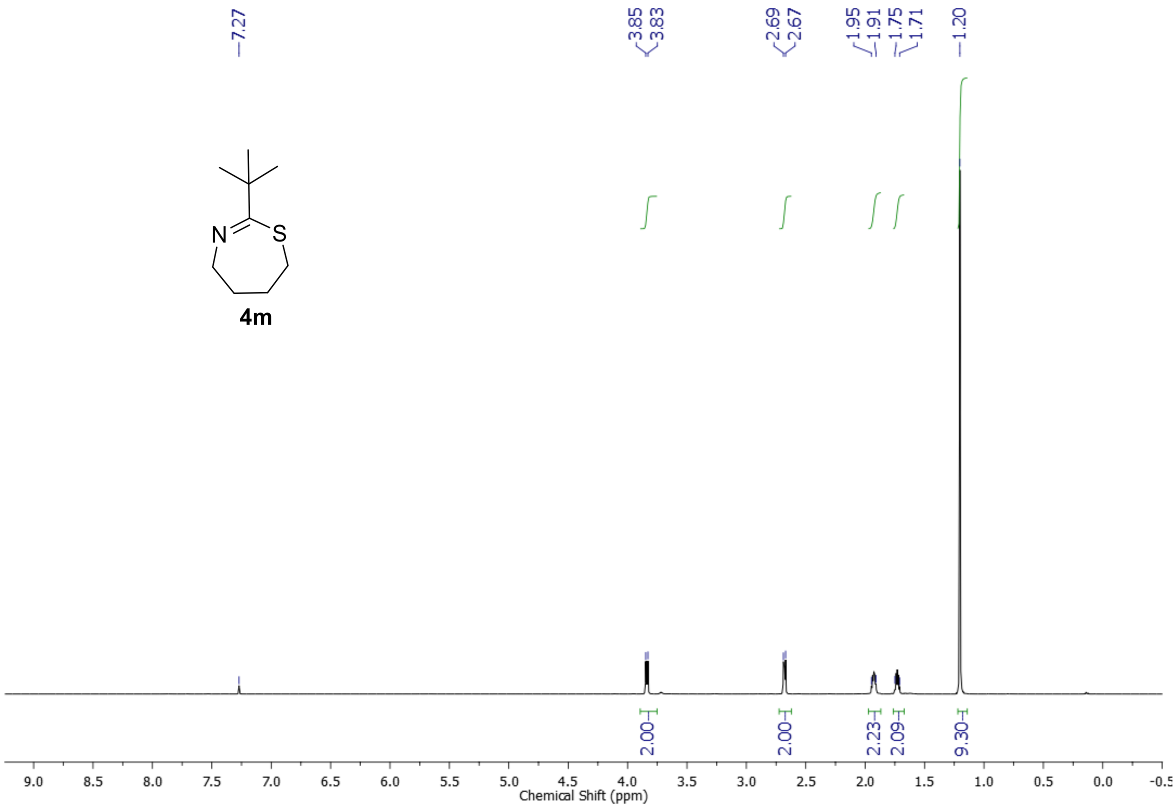

^{13}C NMR (151 MHz, $CDCl_3$) spectrum of compound **4m**

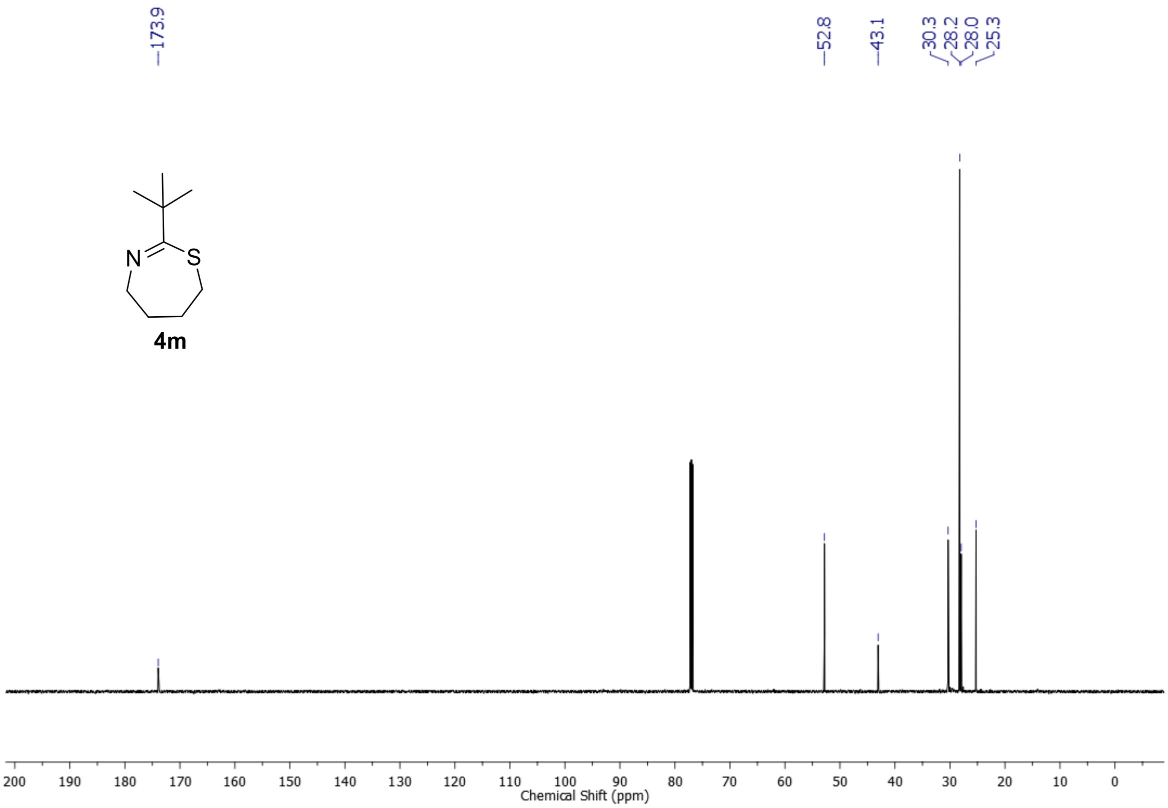

5. References

(1) Wang, J.; Changxiu, L.; Mingzhi, G.; Jiyu, L.; Xianzhong, L.; Jing, M.; Renqi, P. Chinese Patent CN101104589, **2008**.

(2) Inada, H.; Kuwayama, Y.; Noro, M.; Fukagawa, N. Chinese Patent CN103709449, **2004**.

(3) Greger, H.; Hofer, M.; Teichmann, K.; Schinnerl, J.; Pannell, C. M.; Vajrodaya, S.; Hofer, O. *Phytochemistry*, **2008,** *69*, 928.

(4) Nguyen, T. B.; Al-Mourabit., A. *Org. Lett*. **2012**, *14*, 4274.

(5) Nguyen, T. B.; Tran, M. Q.; Ermolenko, L; Al-Mourabit., A. *Org. Lett*. **2014**, *16*, 310.

(6) Wipf, P.; Hayes, G. B. *Tetrahedron* **1998**, *54*, 6987.

(7) Yates, J.; Rosinger, H. P.; Hackmann, J. T. GB Patent 1013441A, **1963**.

(8) Stewart, W. E.; Siddall, T. H. *Chem. Rev.* **1970**, *70*, 517.
